# Brazilian Nutritional Consensus in Hematopoietic Stem Cell Transplantation: Adults

**DOI:** 10.31744/einstein_journal/2020AE4530

**Published:** 2020-01-31

**Authors:** Juliana Bernardo Barban, Belinda Pinto Simões, Bruna Del Guerra de Carvalho Moraes, Cássia Rehem da Anunciação, Cristiane Santos da Rocha, Daniela Cristina Querino Pintor, Daiane Cristina Guerra, Deise Andrade Silva, Edith de Castro Martins Brandão, Fábio Kerbauy, Fernanda Ramos de Oliveira Pires, Graciele Lima Morais, Jayr Schmidt, Juliana Maria Faccioli Sicchieri, Karine Sampaio Nunes Barroso, Luciana Verçoza Viana, Mariana Hollanda Martins da Rocha, Maysa Penteado Guimarães, Natalia Leonetti Couto Lazzari, Nelson Hamerschlak, Plinio Patricia Ramos, Plinio Nascimento Gomes, Priscila da Silva Mendonça, Raquel Candido de Oliveira, Renata Corrêa Scomparim, Ricardo Chiattone, Rosa Wanda Diez-Garcia, Thaís de Campos Cardenas, Thais Manfrinato Miola, Thalita Cristina de Mello Costa, Vanderson Rocha, Andrea Z Pereira

**Affiliations:** 01 Escola Paulista de Medicina Universidade Federal de São Paulo São PauloSP Brazil Escola Paulista de Medicina, Universidade Federal de São Paulo, São Paulo, SP, Brazil.; 02 Hospital das Clínicas Faculdade de Medicina de Ribeirão Preto Universidade de São Paulo Ribeirão PretoSP Brazil Hospital das Clínicas, Faculdade de Medicina de Ribeirão Preto, Universidade de São Paulo, Ribeirão Preto, SP, Brazil.; 03 Hospital das Clínicas Faculdade de Medicina Universidade de São Paulo São PauloSP Brazil Hospital das Clínicas, Faculdade de Medicina, Universidade de São Paulo, São Paulo, SP, Brazil.; 04 Hospital Leforte Liberdade São PauloSP Brazil Hospital Leforte Liberdade, São Paulo, SP, Brazil.; 05 Hospital Santa Marcelina São PauloSP Brazil Hospital Santa Marcelina, São Paulo, SP, Brazil.; 06 Centro de Transplante de Medula Óssea Instituto Nacional de Câncer José Alencar Gomes da Silva Rio de JaneiroRJ Brazil Centro de Transplante de Medula Óssea, Instituto Nacional de Câncer José Alencar Gomes da Silva - INCA, Rio de Janeiro, RJ, Brazil.; 07 IBCC Oncologia São PauloSP Brazil IBCC Oncologia, São Paulo, SP, Brazil.; 08 Universidade Federal de São Paulo São PauloSP Brazil Universidade Federal de São Paulo, São Paulo, SP, Brazil.; 09 A.C.Camargo Cancer Center São PauloSP Brazil A.C.Camargo Cancer Center, São Paulo, SP, Brazil.; 10 Hospital São Rafael SalvadorBA Brazil Hospital São Rafael, Salvador, BA, Brazil.; 11 Universidade Federal do Ceará Hospital Universitário Walter Cantídio FortalezaCE Brazil Hospital Universitário Walter Cantídio, Universidade Federal do Ceará, Fortaleza, CE, Brazil.; 12 Hospital de Clínicas de Porto Alegre Porto AlegreRS Brazil Hospital de Clínicas de Porto Alegre, Porto Alegre, RS, Brazil.; 13 Hospital Samaritano São PauloSP Brazil Hospital Samaritano, São Paulo, SP, Brazil.; 14 Hospital Israelita Albert Einstein São PauloSP Brazil Hospital Israelita Albert Einstein, São Paulo, SP, Brazil.; 15 Universidade Estadual de Campinas CampinasSP Brazil Universidade Estadual de Campinas, Campinas, SP, Brazil.

**Keywords:** Hematopoietic stem cell transplantation, Nutritional status, Nutritional therapy

## Abstract

The nutritional status of patients submitted to hematopoietic stem cell transplant is considered an independent risk factor, which may influence on quality of life and tolerance to the proposed treatment. The impairment of nutritional status during hematopoietic stem cell transplant occurs mainly due to the adverse effects resulting from conditioning to which the patient is subjected. Therefore, adequate nutritional evaluation and follow-up during hematopoietic stem cell transplant are essential. To emphasize the importance of nutritional status and body composition during treatment, as well as the main characteristics related to the nutritional assessment of the patient, the Brazilian Consensus on Nutrition in Hematopoietic Stem Cell Transplant: Adults was prepared, aiming to standardize and update Nutritional Therapy in this area. Dietitians, nutrition physicians and hematologists from 15 Brazilian centers thar are references in hematopoietic stem cell transplant took part.

## INTRODUCTION TO THE TYPES OF HEMATOPOIETIC STEM CELL TRANSPLANTATION

Hematopoietic stem cell transplantation (HSCT) is a well-established treatment modality for a wide range of benign and malignant hematological diseases.^1^

Every year, about 50 thousand individuals undergo HSCT worldwide.^2^ It is estimated that 3,091 HSCT were performed in Brazil, in 2018, based on data from the Brazilian Transplantation Registry, in line with the global trend towards an increase in the number of procedures carried out.^3^ In addition to the progressive increase in the number of transplant centers, this growth is due to the large number of donors registered with the Brazilian Registry of Volunteer Blood Marrow Donors (REDOME - *Registro Brasileiro de Doadores Voluntários de Medula Óssea*) which is now the third largest registry in the world, with more than 4 million people, which provides better chances of finding a suitable donor.^4^

Based on the donor, HSCT can be referred to as autologous, when the hematopoietic stem cells (HSC) used are the patient’s own; allogeneic, when they come from a different donor; and syngeneic, when the donor is an identical twin. In allogeneic transplantation, the donor can be related (HLA matched or unmatched - haploidentical) or unrelated (preferably HLA matched), from the donor registry or the umbilical cord and placental blood bank (UCPB) - HLA matched or partly unmatched. Table 1 describes HSCT modalities. Table 2 lists the main diseases leading to HSCT indication.^5^

**Table 1:** Hematopoietic stem cell transplantation modalities

Types of transplantation	Source of hematopoietic stem cells	Donor
Autologous	Bone marrow	Own patient
	Allogeneic	
	Syngeneic	
Allogeneic	Bone marrow	Related (brother or other relative)
	Peripheral blood	
	Umbilical cord and placental blood	Unrelated (anyone who is not a relative of the patient)
Syngeneic	Bone marrow	Identical twin
	Peripheral blood	

Source: Adapted from Passweg JR, Halter J, Bucher C, Gerull S, Heim D, Rovó A, et al. Hematopoietic stem cell transplantation: a review and recommendations for follow-up care for the general practitioner. Swiss Med Wkly. 2012;142:w13696. Review.^(5)^

**Table 2:** Main indications for hematopoietic stem cell transplantation

Autologous
Malignant disorders
Multiple myeloma
Non-Hodgkin lymphoma
Hodgkin lymphoma
Acute myeloid leukemia
Neuroblastoma
Ewing´s sarcoma
Germ cell tumors
Other unusual cancers of childhood
Non-malignant disorders
Autoimmune diseases
**Allogeneic**
Malignant disorders
Acute myeloid leukemia
Acute lymphoblastic leukemia
Chronic myeloid leukemia (refractory to tyrosine-kinase inhibitors)
Myelodysplastic syndromes (high risk)
Myeloproliferative neoplasm (high risk)
Non-Hodgkin lymphoma
Hodgkin lymphoma
Multiple myeloma
Chronic lymphocytic leukemia
Non-malignant disorders
Acquired
Severe aplastic anemia
Nocturnal paroxysmal hemoglobinuria (not eligible for complement inhibition therapy)
Congenital
Fanconi's anemia and other bone marrow failure syndromes
Thalassemia
Sickle cell disease
Congenital immunodeficiency syndrome
Inborn errors of metabolism

Source: Passweg JR, Halter J, Bucher C, Gerull S, Heim D, Rovó A, et al. Hematopoietic stem cell transplantation: a review and recommendations for follow-up care for the general practitioner. Swiss Med Wkly. 2012;142:w13696. Review.^(5)^

## AUTOLOGOUS HEMATOPOIETIC STEM CELL TRANSPLANTATION

Autologous HSCT is based on the principle of using high-dose chemotherapy drugs followed by salvage HSC, because, otherwise, these doses would not be tolerated by patients due to intense myelotoxicity with very prolonged aplasia. The purpose is to overcome tumor cell resistance to conventional doses of chemotherapy, improving treatment response.^4^ The most frequent indications for autologous HSCT (Table 2) include patients with myeloma, as the current treatment backbone for patients with no major comorbidities; and patients with lymphoma in first remission (such as mantle cell lymphoma) or those with non-Hodgkin or Hodgkin lymphomas relapsing after chemotherapy.

In this treatment modality, the HSC (CD34^+^) are the patient’s own cells, collected by apheresis and cryopreserved to be infused back after high-dose chemotherapy/radiotherapy. Cells are collected after 5 to 6 days of stimulation with granulocyte colony-stimulating factor (GCSF) at 10ug/kg/day infused into a peripheral vein.^5^

The complications of this therapy are related with toxicity of the conditioning regimen, such as mucositis and abnormalities in the liver, kidneys, heart and other organs, in addition to infection-related complications during aplasia.^5^ Although autologous HSCT is a curative treatment modality for several diseases, relapse of the underlying disease is still the major post-transplant complication.

## ALLOGENEIC HEMATOPOIETIC STEM CELL TRANSPLANTATION

Allogeneic HSCT consists in reconstituting the bone marrow by intravenously infusing HSC from a related or unrelated donor after the use of a conditioning regimen. The latter aims not just to eradicate neoplastic cells by direct cytotoxicity, but also to create an immunological space by immunoablation and immunosuppression, allowing for engraftment of allogeneic HSC.^6^

Major histocompatibility complex (MHC) mediated genetic mismatches between donor and recipient trigger the main aloimmune reactions affecting the post-transplant period: rejection, graft *versus* host disease (GVHD) and graft *versus* tumor (GVT) effect. The donor’s T-lymphocytes have been considered the main effectors of these two effects.^7^

The GVT effect has a central role in allogeneic HSCT, since the donor’s alloreactive T-cells are responsible for eliminating residual malignant cells, consequently contributing to a lower risk of relapse of the underlying disease.^7^ Thus, allogeneic HSCT is currently considered a cell therapy with antitumor activity.

## SOURCE OF HEMATOPOIETIC STEM CELLS

For many years, HSC were collected exclusively by multiple bone marrow aspirations through the posterior iliac crest. However, an important milestone in the dissemination of the HSCT technique was the discovery of other HSC sources, such as peripheral cells and UCPB.^5^

## DONOR SELECTION

In allogeneic HSCT, the selection of a donor with the appropriate degree of compatibility is essential for a successful HSCT. The genetic factors with the greatest impact on transplant results include HLA genes.^7^

Of all the possibilities of potential donors for allogeneic HSCT, fully HLA-matched related donors still account for the best overall and progression-free survival rates.^8^ Unfortunately, only 25% to 30% of allogeneic HSCT candidates have a HLA-matched related donor. Therefore, approximately 70% of patients do not have any related donors with no HLA matching. There are three possible HSC sources in this situation: cells from unrelated donors enrolled in bone marrow registries; cells from umbilical cord and placental blood, or those from partially HLA-matched related donors (haploidentical).

## TYPES OF PREPARATION (CONDITIONING) REGIMENS

The preparation or conditioning regimen is intended to reduce and even eradicate an underlying neoplasm, in addition to inducing immunosuppression in the recipient to allow for HSC engraftment. Table 3 lists the conditioning regimens most commonly used in allogeneic HSCT.^5^

**Table 3:** Types of conditioning regimens

Types of conditioning regimens	Considerations
Myeloablative	High-dose chemotherapy/radiotherapy
	Myelotoxic
	High toxicity
Non-myeloablative	Mainly intended for immunoablation/immunosuppression
	Less toxic
Reduced-intensity myeloablative conditioning	Allows for HSCT in older patients or those with comorbidities
	Reduced toxicity, intermediate between MA and NMA

Source: Passweg JR, Halter J, Bucher C, Gerull S, Heim D, Rovó A, et al. Hematopoietic stem cell transplantation: a review and recommendations for follow-up care for the general practitioner. Swiss Med Wkly. 2012;142:w13696. Review.^(5)^

HSCT: hematopoietic stem cell transplantation; MA: myeloablative; NMA: non-myeloablative.

## IMMEDIATE POST-HSCT COMPLICATIONS: TOXICITY, APLASIA AND ACUTE GRAFT-*VERSUS*-HOST DISEASE

After myeloablative conditioning (including all autologous HSCT) and some reduced-intensity therapies, we observe, after HSC infusion, an immediate toxicity phase due to the chemotherapy/radiotherapy administered, which can last for 10 to 15 days. In this phase, patients present nausea, vomiting and diarrhea, which can be associated with mild to severe mucositis, which occurs in 47% to 100% of patients.^9^

The presence of mucositis depends on the conditioning received - the presence of total body irradiation, for example - the age of the patient, the use of methotrexate for GVHD prophylaxis, prior oral hygiene, and some genetic polymorphisms that affect chemotherapy metabolism.^9,10^ In this phase, patients also experience bone marrow toxicity with aplasia and, as a consequence, greater need for transfusion, and higher infection rates, particularly bacterial infections. Due to the high metabolic and inflammatory activity (known as cytokine release syndrome) in this phase, most patients do not eat and require opioids for the pain caused by mucositis, presenting with major weight loss, unless appropriate supplementation is administered. This phase continues until engraftment or neutrophil recovery. However, in this period, there is a risk for acute GVHD, which can manifest in the skin, liver or gastrointestinal tract. In the latter, patients can have nausea, vomiting and diarrhea, sometimes quite voluminous and, in more severe cases, intestinal bleeding and melena. Here, once again, patients have major metabolic consumption, requiring long-term corticosteroids, which is part of GVHD management, and fasting or dietary restriction.

## IMPORTANCE OF NUTRITIONAL STATUS IN HEMATOPOIETIC STEM CELL TRANSPLANTATION

Patients undergoing HSCT are a heteregenous population from the nutritional standpoint, with differences regarding the type of intervention needed, which is why they require distinct strategies. We must also consider the intensity of the conditioning regimen and the presence of GVHD, which have a direct effect on the nutritional status of the patient. Decreased oral intake, post-HSCT complications, and increased nutritional requirements lead to the need for individualized nutritional interventions.^11^

The early identification of patients at nutritional risk minimizes the deleterious effects of malnutrition and/or overweight/obesity. Both are risk factors for complications and increased mortality, either or not associated with relapse in transplanted patients.^12^ However, one of the difficulties which limits the impact of body weight (BW) on post-transplant prognosis is that most data available are limited to the first year after HSCT, in addition to the difference in methods applied and cutoff points used for nutritional status classification.^13^ Initial and serial assessment of nutritional status is important, since it anticipates potential nutritional impacts of conditioning and other treatment-related toxicities, which affect nutrient intake, absorption and utilization.^11^

## OBESITY

Excess weight (overweight and obesity) is an important risk factor for cardiovascular diseases, *diabetes mellitus*, among others. The prevalence of obesity increases every year. According to the World Health Organization (WHO), in 2016, 39% of adult population was overweight and 13% of the world population was obese.^14^It is not different in Brazil, where the agency Telephone-based Survey of Risk and Protective Factors for Chronic Diseases (VIGITEL - *Vigilância de Fatores de Risco e Proteção para Doenças Crônicas por Inquérito Telefônico*)^15^ showed, in 2016, that more than half of the population was overweight and 18.9% of Brazilians were obese. Thus, it is increasingly common to have overweight or bariatric patients requiring bone marrow transplantation.

Several publications have shown obesity as an independent prognostic factor for adverse events, such as increased mortality, risk of infection, incidence of acute grade II-IV GVHD, and toxicity. Tarella et al.,^16^ in a retrospective study with patients with non-Hodgkin lymphoma undergoing autologous bone marrow transplantation, showed worse results, with a 2.9-fold higher risk of death compared to the group of non-obese patients. Meloni et al.,^17^ also showed that obesity is a positive predictive factor for increased treatment-related toxicity and mortality in patients with acute myeloid leukemia (AML) undergoing autologous bone marrow transplantation. The number of nucleated cells used in transplantation, which is smaller in obese patients, may influence transplant outcomes.^18^ Vogl et al.,^19^ did not show any effect of overweight or obesity on progression-free survival, overall survival, progression and mortality related to relapse in patients undergoing autologous transplantation following multiple myeloma. In the same study, obese patients subjected to conditioning with melphalan and total body irradiation had a lower relapse rate; however, the rationale is uncertain.

Nevertheless, in a 2014 meta-analysis published in Bone Marrow Transplantation, the negative impact of obesity on bone marrow transplant outcomes was statistically significant for allogeneic and non-autologous transplants.^20^

As for GVHD, there are studies that support increased incidence of acute grade II-IV GVHD as the body mass index (BMI) increases.^18^

Obesity is also associated with a higher incidence of infectious diseases (bacterial, fungal and viral) due to increased hyperglycemia when compared to patients with a BMI under 30kg/m^2^.^18^

Obese patients may experience changes in drug pharmacokinetics. Some drugs are not fat soluble and do not disseminate well in fat tissue.^17^ The clearance of a drug is the most relevant pharmacokinetic parameter due to its correlation with clinical outcomes. The liver is the main organ responsible for drug metabolism, and hepatic steatosis, very common in obese individuals, may have an impact on drug clearance*.*^21^ No positive relation has been shown between obesity, veno-occlusive disease of the liver, and liver dysfunction in these patients.^18^ The effect of obesity on the kidneys is not very clear yet.^21^

The American Society for Blood and Marrow Transplantation (ASBMT), in a 2014 review, did not find any significantly strong evidence that could specifically guide the prescription of conditioning chemotherapy to obese patients. The drugs are suggested empirically or by extrapolation of data from non-transplanted patients.^22^ The American Society of Clinical Oncology (ASCO) reports that the poorer outcomes of chemotherapy in obese patients have multifactorial causes – however, the unexplained reduction in drug doses partly explains the higher mortality among overweight and obese patients. ASCO recommends that doses should be calculated as per the real weight of patients.^21^

Hyperglycemia, when occurring during neutropenia, is a risk factor for acute GVHD, and associated with higher non-relapse mortality. Obesity is related with hyperglycemia and insulin resistance. Hyperglycemia also increases the incidence of infectious diseases.^18^

Metabolic syndrome is becoming more prevalent in post-transplant patients. Higher levels of C-reactive protein (CRP) and leptin, and lower levels of adiponectins may be indicative of association with inflammation. Leptin, which is proportional to the percentage of fat in the body, affects the function and proliferation of regulatory T-cells (Treg), suppressing their activity and increasing the risk of acute GVHD.^18^ A higher level of visceral fat is found in survivors.

## PROTEIN-CALORIE MALNUTRITION

Even with the current epidemiological transition, there is still a considerable percentage of patients with sarcopenia^23^ and protein-calorie malnutrition, and patients at nutritional risk.^24^

We have known for decades that malnutrition negatively affects the outcomes of HSCT. A retrospective study with 2,238 patients undergoing autologous and allogeneic transplantation found a significant association between weight loss within 150 days of HSCT and a worse prognosis.^28^

A study looking at 544 adult patients undergoing allogeneic transplantation found higher mortality from infection and disease relapse in patients with BMI under 20kg/m^2^.^29^ Weight loss over 10% is also associated with higher post-allogeneic-transplant mortality.^26^

In a stage of protein-calorie malnutrition, involuntary weight loss and decreased levels of plasma proteins, such as albumin, there is a high risk of toxicity from chemotherapy and the other drug classes administered.^30^ Albumin is the most abundant protein in human plasma, and it has multiple roles in body homeostasis, and an essential role as a drug-transport protein. This may have pharmacokinetic implications on clinical therapy, affecting plasma concentrations of chemotherapeutic agents.^31^

Pre-HSCT hypoalbuminemia has a significant impact on these patients’ survival. Patients with albumin levels under 3.2g/dL have significantly lower disease-free survival than those with levels over 3.2g/dL.^32^ Low BW also impairs tolerability to treatment, contributing to higher chemotherapy-mediated toxicity due to related changes in drug pharmacokinetics.^33^

Another major downside of an impaired nutritional status in patients undergoing HSCT is the longer period of aplasia during the transplant, in which low BMI, hypoalbuminemia, and increased urinary nitrogen excretion are associated with a longer time to neutrophil engraftment, leading to increased susceptibility to infections,^34^ in addition to financial losses, since these patients usually require prolonged hospitalization.^30^

In addition to the anticipated harm, protein-calorie malnutrition and low oral intake are associated with increased GVHD rates^35^ − particularly of the lungs, gastrointestinal tract and oral cavity. Vitamins A and D deficiency is also associated with higher incidence and severity of GVHD.^36^

In this sense, it is recommended that the pre-HSCT nutritional assessment be conducted within 30 days before admission for HSCT, aiming to restore or maintain the pre-HSCT nutritional status and correct any nutritional deficiencies, with the view to minimize the deleterious effects of HSCT on nutritional status.

By evaluating the nutritional status, changes in food intake and nutritional semiology, it is possible to define groups of patients at a higher risk, allowing for rapid and effective nutritional intervention to curb protein catabolism.^37^

## NUTRITIONAL ASSESSMENT

Patients undergoing HSCT must be screened immediately upon admission for the procedure, or for any other hospital admission related with complications from the treatment or conditioning regimens administered. They are usually considered at nutritional risk or already malnourished, due to their underlying disease,^38^ the chemotherapy received, and treatment toxicity. The different cytotoxic agents, radiation therapy, and other new drugs used in oncology-hematology treatment affect not only tumor cells, but also healthy cells, particularly those with high replication rates, such as lymphocytes and gastrointestinal tract cells (enterocytes and colonocytes). The effects on these cells lead to major functional changes in the digestive tract and the immune system, resulting in malabsorption, and seriously impairing the patient’s nutritional status.^39,40^ Reduced-intensity conditioning (RIC) regimens are mostly indicated for older patients, or individuals with multiple comorbidities, which would be a contraindication for myeloablative HSCT. Reduced-intensity conditioning reduces side effects, such as mucositis, and the duration of neutropenia.^41,42^Allogeneic HSCT has considerable toxicity and leads to major inflammation, in addition to metabolic changes (such as cachexia), gastrointestinal symptoms and generalized effects, which can result in reduced diet intake and deterioration of the nutritional status. This behavior clearly puts patients at nutritional risk, which, additionally to the treatment, will negatively affect clinical outcomes.^43^

Considering a chronic disease, like cancer, and factors detrimental to the patient’s condition or clinical response to the treatment (such as age and psychosocial factors), there is a great chance of an impaired nutritional status at important stages of the treatment − and particularly during HSCT. Some diagnoses consider weight loss, muscle wasting, and inflammation, and characterize the presence of cachexia into different phases (pre-cachexia or refractory cachexia).

Of the 503 patients assessed in Germany^44^ for the presence of cachexia as per the criteria by Fearon et al.,^45^ (weight loss <5% for the past 6 months, or weight loss of 2 to 5%, combined with BMI <20kg/m^2^, or weight loss of 2 to 5% combined with the presence of sarcopenia), 131 were diagnosed with cachexia, of which 15.2% were hematology patients. The authors showed that patients with cachexia had multiple symptoms, such as anemia and impaired kidney and liver function (cholestasis).

However, in order to standardize the discussion, we will address only the diagnosis of malnutrition or low weight, based on anthropometric measurements.

Nutritional status assessment with a validated tool before, during and after HSCT is still little discussed and documented, but it is certainly needed. There is no consensus in the literature about different assessment methods to be used in each phase, but it is a fact that this assessment must take place according to each institution’s protocol and comprise the main phases of HSCT: admission; onset of preparation chemotherapy; day of the HSC infusion; onset of immunosuppression; hospital discharge; 1 month and 3 months after HSCT; 6 months and 1 year after allogeneic HSCT (in an outpatient setting).

The American Society Parenteral and Enteral Nutrition (ASPEN)^40^and the European Society Parenteral and Enteral Nutrition (ESPEN),^46^ in their consensus statements, recommend nutritional screening and intervention if the patient is incapable of maintaining their nutritional status. The latest consensus for oncology patients, from 2017,^47^ recommends that, for early detection of nutritional disorders, food intake, weight changes and BMI be regularly assessed, starting on cancer diagnosis, and reviewed according to the patient’s clinical stability. It states that, for this purpose, parameters such as BMI, weight loss and food records must be used, or a validated screening tool, like the Nutritional Risk Screening (NRS-2002), Malnutrition Universal Screening Tool (MUST), Malnutrition Screening Tool (MST) or Mini Nutritional Assessment Short Form (MNA-SF). It is worth noting that an abnormal screening result (presence of risk), alone, does not provide enough information for developing a patient’s plan of care. Patients at risk must be followed and evaluated by more specific tools, and only then can an intervention be designed. Therefore, ESPEN strongly recommends that, after identification of the risk, the patient undergo an objective and quantitative evaluation of food intake, impact of symptoms, muscle mass, physical performance and grade of systemic inflammation. It also advises that this evaluation be repeated at frequent intervals (*e.g*. fortnightly, monthly, every 6 months, as appropriate). The tools listed for this step are: Subjective Global Assement (SGA), Patient-Generated Subjective Global Assessment (PG-SGA) or Mini Nutritional Assessment (MNA).

Most concerning to patient care teams and HSCT center managers is that nutritional risk is associated with increased mortality and higher hospital costs.^48^

Thus, when caring for HSCT recipients, it is vital that nutritional screening and assessment be performed upon admission, as well as during outpatient care before admission, while preparing for HSCT. The frequency must be based on the presence or absence of nutritional risk, must not exceed 15 days for outpatients already at nutritional risk, and 30 days for those not yet at risk. Inpatients must be screened up to 48 hours after admission and reassessed after 7 days. This weekly reassessment must be carried out until patient is discharged home.^40,49^

The screening period can change, depending on quality criteria of the nutritional care. Nutritional risk screening carried out within 24 hours of admission is one of the requirements of quality audit processes such as that of the Joint Commission International (JCI).^50^

There is no definition regarding the best tool to be used in reassessing cancer patients. Many are not valid for reevaluations. However, in fact, hematological cancer patients undergoing HSCT must be followed and reevaluated on a regular basis. Nutritional visits allow for early and important interventions at each treatment stage, and changes in the nutritional or functional status must be identified. The tools that allow for calculation of final diagnostic scores can be an alternative to evaluate patients at different time points. Functional or quality of life assessments, in this sense, can be important tools to guide adjustments over the course of treatment (good examples included the PG-SGA and dynamometry for function assessment of muscle strength).

## TOOLS FOR NUTRITIONAL ASSESSMENT

### NRS-2002

The tool most suitable for assessing the nutritional risk of inpatients is the NRS-2002, which takes into account the patient’s age (added to the final screening score), includes all clinical and surgical patients,^51^ and is recommended by the ESPEN for in-hospital screening. It has been validated in a review of 128 randomized controlled nutritional support trials, to determine whether it could distinguish patients with positive clinical outcomes due to nutritional intervention from those that did not benefit from Nutritional Therapy (NT).^52^ NRS-2002 is the first nutritional screening tool developed on the ground of evidence-based medicine.^51,53^

Also, it has shown a high predictive value and low interobserver variation (k=0.67).^53^ Nutritional risk is assessed by the combination of current nutritional status and disease severity, with the first including as variables the BMI, recent weight loss and diet intake in the week before hospital admission.

In a national study of HSCT centers in Switzerland^24^ with a total of 621 transplants carried out in 2014 (226 allogeneic and 395 autologous), all centers involved in allogeneic HSCT (n=3) were conducting nutritional screening mostly based on the NRS-2002. Only one center was using a screening method developed in-house, incorporating indirect calorimetry (IC) parameters and bioelectrical impedance. As for centers conducting autologous HSCT (n=7), only half had nutritional screening protocols. Three centers would call on a dietitian only if malnutrition was suspected during patient hospitalization.

Liu et al.,^54^ investigated in 99 Chinese patients with leukemia, whether NRS-2002 was capable of properly assessing risk before and after HSCT, and if there were any age- or sex-related differences. The mean age was 32.4 years and the mean time in a laminar flow room was 30 days. Two dietitians were in charge of applying the tool upon admission (before HSCT) and the day the HSCT was completed (after). Out of patients screened, 22.2% were at nutritional risk. At the same time, 15.4% of patients with BMI ≥18.5kg/m^2^ were at nutritional risk, showing that the BMI alone would not have accurately identified the presence or absence of nutritional risk. Patients were grouped by sex, age and other conditions, and the presence of risk was compared. The authors showed there is no significant difference in the presence of risk by sex; however, they found a difference for age, matching degree (patients under 30 years and not fully-matched have higher nutritional risk), weight loss and decreased food intake or BMI (p<0.01). All patients were identified as at nutritional risk after HSCT, since the transplant alone, according to the authors, already confers score 3 in disease severity (*i.e*. nutritional risk), showing that the NRS-2002 is not an appropriate tool at this stage. However, the authors pointed out that 63,6% of patients had a score of 6 (the maximum score, considering there were no patients over 70 years in the sample), which means that, although the NRS-2002 cannot distinguish risk after HSCT, patients had higher scores than before the procedure. In addition, 77.8% of patients lost weight after HSCT, of which 63.6% lost >5% at one month.^54^

### Subjective Global Assement and Patient-Generated Subjective Global Assessment

The SGA is an instrument commonly used to assess the nutritional status of inpatients. It is a clinical method developed by Detsky et al.,^55^ capable of assessing nutritional risk and, most importantly, identify patients in need for more specific NT.^55^

The SGA, when applied sequentially after the NRS-2002, increases the sensitivity and specificity of both tests in predicting adverse clinical outcomes. Thus, NT can be targeted for patients in true nutritional debt.^56^

The SGA showed an association with scores Acute Physiology and Chronic Health Evaluation II (APACHE II) and Simplified Acute Physiology Score II (SAPS II), as well as with mortality in a descriptive cohort of 124 clinical and surgical critically-ill patients.^57^

The limitation of the SGA is that, as the name says, it is subjective, and depends on the experience of the observer.

In 1995, an SGA adapted for cancer patients was validated: the PG-SGA. It is a self-applicable questionnaire with two parts. In the first part, patients themselves answer the questions, describing any changes in weight, food intake, cancer-related symptoms, and changes in functional capacity. In the second part, answered by the professional applying the questionnaire, questions are based on diagnosis-related factors that may increase metabolic demand, such as stress, fever, depression, fatigue, tumor staging or cancer treatment, and physical examination.^58^A disadvantage of this method is the fact that some patients may have difficulties answering questions related to weight loss in previous months, as well as to describe food intake in the previous month, which may, in some instances, result in a different nutritional status classification.^59^

Patient-Generated Subjective Global Assessment has the advantage of letting patients have an active role, and it optimizes the time spent by the professional to complete the evaluation. It is appropriate to identify cancer patients who would benefit from preventive nutritional intervention during cancer treatment.^60,61^ The limitations are related with patients’ lack of understanding of the questions relative to weight loss, and how to describe their food intake.^60^The PG-SGA has high sensitivity and specificity, at 80% and 89%, respectively.^62^

Barritta de Defranchi et al.,^63^ in a prospective, longitudinal cohort study, described and compared the nutritional status of 123 patients undergoing HSCT, admitted to a teaching hospital in Buenos Aires, at three different time points: within 24 hours from admission for transplant (usually 3 to 7 days before transplant for chemotherapy), upon discharge, and at the follow-up visit (10 days after discharge). The nutritional status was assessed by the BMI and the PG-SGA. Of the total patients, 36 had missing data in some of the study evaluation phases for different reasons, such as readmission, transfer to the, and even death. The mean age was 50.5 years. Multiple myeloma was the most frequent diagnosis (44.8%). Most subjects (80.5%) had been subjected to autologous HSCT. The authors found a significant difference between nutritional status on admission, discharge and follow-up: the average PG-SGA scores were 3.39 on admission, 12.81 on discharge, and 6.71 on outpatient follow-up (p<0.001). Most inpatients (94.3%) were well nourished upon admission. Considering the BMI, there were no low-weight patients in the sample, and most were overweight (34.1%) or obese (33.3%). Upon discharge, 59.7% were malnourished (PG-SGA B and C). Patients with a length of stay longer than 21 days scored 2.9 points higher in the PG-SGA than patients who stayed for less than 21 days (p=0.034). Patients under 60 years had worse PG-SGA scores (p=0.0007). Patients undergoing allogeneic HSCT had a higher score on discharge (*i.e*., poorer outcome) than those undergoing autologous HSCT (p=0.0152). On outpatient follow-up, the PG-SGA score was even higher than the score on admission (3.66 points higher). A large part of the patients (74.7%) has a score ≥4, requiring immediate nutritional intervention. Body weight increased from discharge to the follow-up visit (mean of 74.5kg to 75.4kg) but was still lower than on admission (mean weight 78kg).

### Malnutrition Universal Screening Tool

The malnutrition universal screening tool is mostly used in protein-calorie malnutrition, which includes three clinical parameters (BMI, involuntary weight loss in the last 3 to 6 months, acute disease or fasting exceeding 5 days) and assigns to each item a score of zero, 1 or 2, as per the following description:

- BMI >20kg/m^2^, score 0; 18.5-20kg/m^2^, score 1; under 18.5kg/m^2^, score 2;
- Weight loss under 5%, score 0; 5% to 10%, score 1; over 10%, score 2;
- Acute disease or fasting exceeding 5 days, if absent, score 0; if present, score 2.

After the first three steps, all scores are added up to calculate a general risk for malnutrition. A score over 2 rates the patient as at high risk for malnutrition; a score of 1, medium risk for malnutrition; and score 0, low risk for malnutrition. For each nutritional risk category, guidelines are offered to help healthcare professionals act based on the results proposed.^64,65^

## ANTHROPOMETRY

Cancer-associated malnutrition involves a prior and ongoing history of nutritional *deficits*, and leads to changes in body composition, including loss of body fat and lean mass, resulting in weight loss and changes in other anthropometric parameters.^66^

Anthropometric measures are of great importance for establishing determinants of malnutrition and overweight, among others, as an instrument of nutritional surveillance.^67^

The anthropometric measures most commonly used in clinical practice to assess the nutritional status of cancer patients include BW, height, BMI, triceps skinfold, arm circumference (AC) and arm muscle circumference (AMC).^68^ However, in clinical practice, due to some mobility restrictions, presence of accesses, edema or immobility, the use of said parameters is not always possible. The inclusion into protocols should be evaluated with caution, even for comparing the diagnosis found to other tools applied (such as subjective assessments). An interesting option is to use an anthropometric indicator for comparing changes over time.

Anthropometry is the most commonly used method because it is non-invasive, easy to execute, fast, low-cost, feasible at the bedside, and yields reliable results if performed by trained professionals. The downside is that it is not capable of detecting recent disturbances to the nutritional status and identifying specific nutritional deficiencies. Of 226 adult outpatients with cancer followed-up in a Spanish study, 64% were malnourished (as assessed by the PG-SGA), and the BMI was not useful for NT-related decision-making, since half of the population (56.5%) had appropriate BMI; however, they observed that, as the BMI decreased, nutritional difficulties increased.^69^

## WEIGHT AND BODY MASS INDEX

The weight is the sum of body compartments and reflects the protein-calorie balance of an individual. To measure weight, a weighing scale (platform or electronic) with 100g precision is needed. It must be installed on a level floor; the individual should be standing on the center of the scale, wearing as little as possible, barefoot, standing straight, with their feet together and arms extended alongside their body.^70^

The weight must be corrected for excess fluids (*e.g*. pleural edema, ascites and/or edema of any nature).^47^

Japanese researchers evaluated 48 patients with lymphoma or myeloma undergoing autologous HSCT at a teaching hospital, in respect to the presence of dysgeusia as assessed via interview (divided into patients who developed dysgeusia during the treatment – 42% – and those who did not – 58%). The use of cryotherapy with ice chips before melphalan (120 minutes in total for each regimen) was significantly lower in the group that developed dysgeusia. Univariate and multivariate logistic regression analyses showed that the chemotherapy regimen used, and the presence of oral mucositis were independent risk factors for dysgeusia, while cryotherapy was an independent suppressor. There were no differences in weight loss between the groups; however, both lost approximately 4kg after autologous HSCT.^71^

Aoyama et al.,^72^ studied 51 patients (mean age 51 years) undergoing allogeneic HSCT in a stem cell transplant department in Japan, in respect to weight loss, with some controlled variables in the NT indication flow chart at the study site. The items were assessed 1 to 2 days before the onset of pre-treatment and at the end of parenteral nutrition therapy (PNT; which can be indicated as a supplement), up to Day 100. Patients were divided into two groups: those who lost ≥7.5% of their weight in the past 3 months, and those who lost <7.5% in the same period. The authors found a strong, significant correlation (r=0.89; p<0.0001) between weight variations and changes in body composition (assessed by bioelectrical impedance). However, a weak correlation was found between weight variations and changes in fat mass (r=0.42; p=0.002). The mean BMI before the onset of pre-treatment was 22.1kg/m^2^, and weight loss was 4.5%. Acute GVHD was more frequent and severe in patients with weight loss ≥7.5%, suggesting that the nutritional status likely affects post-HSCT clinical outcomes. This group also showed higher scores for nutrition-related adverse events, and for a longer duration. The authors pointed out in the study that nutrition-related adverse events (such as nausea, mucositis, stomatitis, abnormal taste, anorexia and vomiting) were associated with increases in performance status, and it would be interesting if nutritional interventions would consider the severity of said adverse events.

Yang et al.,^73^ in a retrospective cohort study, investigated 267 Chinese patients (66.7% aged ≤40 years) diagnosed with AML or acute lymphoid leukemia (ALL), receiving total body irradiation or busulfan + cyclophosphamide as conditioning regimens. Patients were periodically monitored after HSCT until death or the last follow-up – 13 years of follow-up (mean group follow-up 19.7 months). Body mass index was sorted into patients with a lower BMI (low and normal weight as per the Chinese evaluation criteria, *i.e*. Body mass index <23kg/m^2^) and higher BMI (overweight and obesity). Of the total, 9.7% had low weight; 51.3%, normal weight; 16.9%, overweight; and 22.1%, obesity. There were no differences between BMI groups except for age (> and ≤40 years). A total of 93 patients (34.8%) died during the study follow-up. The overall survival was lower in the lower-BMI group, compared with the higher-BMI group (BMI ≥23kg/m^2^), with p=0.041. Within 3 years of HSCT, the overall survival was 55.7% for the lower-BMI group and 72.3% for the higher-BMI group. Compared with low-weight patients, normal-weight, overweight and obese patients had a lower odds ratio (OR), with a significant trend towards a higher overall survival as the BMI increased (p=0.019), after adjusting for age, sex, diagnosis, disease staging, type of graft, conditioning regimen, GVHD prophylaxis regimen, and time from diagnosis to transplant. Also, patients with a higher BMI survived longer, with a significant decrease of about 40% in the OR, compared with the low-BMI group. Authors believe that the difference in survival found between BMI groups (lower and higher) must be attributed to differences in the metabolism of chemotherapeutic drugs used for conditioning. Overweight and obese patients possibly have higher cumulative doses of the drugs or longer exposure times, which may lead to better outcomes.

Jaime-Pérez et al.,^74^ looked at 77 patients with malignant or benign hematological diseases undergoing 6/6 HLA-compatible HSCT (64 allogeneic and 13 autologous), in an outpatient management model, treated at a teaching hospital in Mexico. The conditioning program consisted of a RIC regimen with ideal-body-weight-based dosing. There was no difference in mortality between patients with BMI <18 and ≥18kg/m^2^). Body mass index changes were recorded at each visit or at least every month. The data were reviewed in the first 6 months after HSCT and, then, every 3 months, until 1 year. Based on the BMI, 18.2% had low weight; 24.7% had normal weight; 33.7% were overweight and 23.4%, obese. There was a significant difference in patient weight before (mean weight 68.3kg) and after HSCT (mean weight 56.3kg), with p=0.014, but not in fat percentage or BMI (p=0.458 and p=0.067, respectively). There was no correlation between BMI > or <30 and the time to bone marrow engraftment (p=0.404) or duration of neutropenia – neutrophils <500x10^9^/L (p=0.014). In the Kaplan-Meier curve for overall survival, there was no difference between the BMI and survival subgroups (p=0.059), however, when survival was compared between obese patients (n=18) and the rest of the group, a longer survival was seen for obese patients (p=0.017), for both autologous and allogeneic HSCT. There was no correlation between BMI and onset of GVHD in the allogeneic HSCT group investigated.

Espinoza et al.,^75^ investigated 18 and 32 patients in Chile undergoing autologous and allogeneic HSCT, respectively, with a mean age of 41 years, of which 84% received myeloablative conditioning and 16% RIC. Nutritional assessment was conducted at the time of the HSCT, 10 days after, and upon discharge. The mean length of stay was 32 days and the mean follow-up was 41 months. On admission, there was no significant difference in nutritional parameters between the autologous and the allogeneic groups, and a significant difference in BMI after HSCT (27.3kg/m^2^ to 26,3kg/m^2^) was found only in the autologous group. Considering both types, the BMI decreased post-HSCT, compared to the pre-HSCT BMI (26.9kg/m^2^ to 26,1kg/m^2^), with p<0.01.

## DYNAMOMETRY

The muscle capacity of malnourished individuals is significantly reduced, since protein-calorie malnutrition directly affects the loss of all skeletal muscle fibers and, as a consequence, decreases muscle strength.^76^ Hand dynamometry, or hand grip strength, is a method used for nutritional assessment of patients, as a prognostic marker, and also in cancer patients. It is easy to apply, simple, fast and low-cost, and it predicts muscle function status.^77^ Dynamometry is indicated as an auxiliary tool in nutritional screening.^49^

The devices used for this grip strength measurement can be classified into four categories, hydraulic, pneumatic, mechanical and strain gauges (or load cells).^78^

Pastore et al.,^79^ assessed the impact of nutritional status and muscle strength on the quality of life of 77 patients with gastrointestinal and lung cancer, referred for chemotherapy for the first time, in southern Brazil. Patients has their nutritional status assessed by BMI and SGA, and muscle function by dynamometry (Jamar mechanical dynamometer). Three measures were obtained for each hand (dominant and non-dominant), and the highest was used. The general health status and quality of life domain was assessed with the European Organization for Research and Treatment of Cancer – Quality of Life Questionnaire Core-30 (EORTC QLQ-C30). Based on the BMI, 60% of sample had normal weight, however, the SGA found only 13% of well nourished patients. The mean quality of life score was 68±21.3. The mean grip strength in the non-dominant hand was 25.7±10.1Kgf, with no association with the quality of life (p=0.3). A poorer nutritional status was associated with lower quality of life scores, by both BMI (p=0.04) and SGA (p=0.007). There was a high prevalence of malnutrition, as per the SGA, and the nutritional status significantly affected the general health status and quality of life, however there was no correlation between nutritional status and muscle strength.

A study conducted in Switzerland with 37 patients with unresectable lung cancer found that hand dynamometry can be useful to assess functional and nutritional status.^80^ It can be included in the evaluation of cancer patients along with other nutritional assessment tools. Nutritional status was obtained by the PG-SGA, and muscle function was assessed with a Jamar hand dynamometer in the non-dominant hand. Three measurements were obtained, and the highest measure was used. In the PG-SGA, 73% (n=27) of patients were moderately malnourished, and 8% (n=3) were severely malnourished. In total, 81% (n=30) were malnourished. The hand grip strength was below the 50^th^ percentile in 57% of patients (n=21). There was a significant association between nutritional status and hand grip strength (p=0.026).

Tanaka et al.,^81^ investigated 34 patients with hematological disease undergoing allogeneic HSCT at a hospital, in Japan. The chemotherapy regimen was myeloablative for 14 patients and non-myeloablative for 20. A rehabilitation program was carried out five times a week, 7 days before the transplant, consisting of strength training and aerobic exercises, 20 minutes per day. The patient was followed-up until continuous infusion of immunosuppressants was discontinued (approximately 30 days after the transplant). In addition to tests assessing physical function (including hand grip), nutritional status was assessed at five different timepoints: upon admission, at the start of the preparation chemotherapy regimen, on the day of the transplant, when immunosuppression was started, and on discharge. Nutritional status was assessed based on calorie intake, weight changes, albumin, CRP and circumferences (for muscle measures). Sarcopenia was defined as per the criteria of the European Working Group on Sarcopenia in Older People (EWGSOP).^82^ The weight was significantly lower after HSCT than before (58.6±15.6kg and 56.0±14.5kg), p=0.001. Hand grip strength and upper-and lower-limb circumference decreased, bilaterally and significantly, after HSCT. Muscle strength significantly decreased by 6%, one month after HSCT, but recovered 3 months later. However, for patients with acute GVHD requiring corticosteroids, muscle loss was about 16% within 3 months after HSCT. The univariate analysis showed that oral energy intake after HSCT was a significant factor associated with weight loss. The authors concluded weight was a sensitive sarcopenia indicator; however, circumference was not the best method to assess muscle strength. In that study, the circumference varied significantly after HSCT, but the reduction was small. Nevertheless, patients with sarcopenia before HSCT are more susceptible to adverse events than patients without sarcopenia − and this explains the importance of this assessment.

## COMPARISONS

Liu et al.,^25^ assessed the nutritional status of 170 patients with hematological disease in Beijing, subjected to allogeneic HSCT, using anthropometric parameters and four tools: NRS-2002, MNA, SGA and MUST. The group included 116 men and 54 women, mean age 30 years (12±56), 65 cases of AML, 63 cases of ALL, 14 cases of chronic myeloid leukemia (CML), 3 lymphomas, 3 aplastic anemias and 22 myelodysplastic syndromes. Patients were assessed before undergoing HSCT and entering laminar flow rooms, and within 48 hours after leaving the room (mean length of stay was 30 days). Only 109 patients were assessed on the same day they left the room. Assessments were carried out by two dietitians. The treatment duration was 30 (±2.1) days. After HSCT, patients had a significant decrease in weight, hip circumference, waist-hip ratio, calf circumference, and AC, when compared to pre-HSCT measures. Before HSCT, the NRS-2002 identified that 21.2% of patients were at nutritional risk, compared to 100% after HSCT. The MUST indicated that before HSCT, 11.8% of patients had high nutritional risk, compared to 59.6% after HSCT. The MNA assessed that 0.06% of patients were malnourished before HSCT, compared with 19.3% after HSCT. The SGA identified that, before HSCT, 1.76% of patients had mild to severe malnutrition, a rate that increased to 83.3% after HSCT. The authors concluded that nutritional risk and malnutrition significantly increase after HSCT. A comparative analysis of nutritional screening after HSCT using the MUST yielded significantly different results than those obtained with the NRS-2002. The SGA indicated that 83.3% of patients had moderate to severe malnutrition, while the MNA identified 19.3% of patients as malnourished; this was probably due to the subjectivity of the SGA, which increased the likelihood of malnutrition when used for assessment. Irrespective of the assessment method, the evidence showed patients with hematological diseases suffer from a significantly deteriorated nutritional status after transplantation, and are at a relatively higher risk.

In a prospective study with 108 patients with leukemia after HSCT, Wang et al.,^83^ assessed the nutritional status using different assessment methods (NRS-2002, MNA, MUST and SGA). The study included 77 men and 31 women, with a mean age of 8 (±56) years, 61 cases of acute lymphoid leukemia (ALL), 15 cases of AML, 14 cases of CML, and 19 cases of myelodysplastic syndrome (MDS). A total of 108 patients completed the SGA and 99 patients completed the NRS-2002, MNA and MUST. During the treatment, 85.2% of patients lost weight, with 50% losing more than 5%, and 42.6% significantly reducing their food intake. To assess the nutritional risk, the positive risk rates using the NRS-2002, the MNA and the MUST were 100%, 74.7% and 63.6%, respectively. There was a significant difference (p<0.05) between the NRS-2002, MNA and MUST. In the malnutrition assessment, the positive rate of the SGA (83.3%) was significantly higher than that of the MNA (17.2%), with p<0.05, and the rate of nutritional risk among leukemia patients aged ≤30 years was higher than that of patients >30 years (p<0.05). The authors suggested the NRS-2002 may be used to assess nutritional deficiencies, but its specificity is not high. The MNA is applicable for nutritional risk screening in the elderly, however it is not suitable for nutritional assessment. Thus, the combination of different screening tools and clinical laboratory indicators would be most recommendable for a precise and comprehensive assessment of nutritional status, providing better diagnostic accuracy.

## SUMMARY TABLE

Table 4 presents suggested approached for nutritional screening and assessment of adult hematological cancer patients scheduled for HSCT at different treatment stages.

**Table 4:** Summarized nutritional screening and assessment of adult hematological cancer patients at different stages of hematopoietic stem cell transplantation (HSCT)

Phase	Assessment
Outpatient	All patients, irrespective of the type of HSCT, must be assessed. Frequency: with nutritional risk up to 15 days, and without nutritional risk, up to 30 days. NRS-2002, PG-SGA or SGA Unintentional weight loss
Admission for transplant	Nutritional screening within 48 hours NRS-2002, PG-SGA or SGA Dynamometry Diet history Patients must be assessed on a weekly basis, during the entire duration of hospitalization
Onset of conditioning	Food intake <75% of nutritional requirements in past 2 weeks Percentage of weight loss
Day of transplantation	Gastrointestinal nutrition-impact symptoms for more than 3 days or every other day in past week Percentage of weight loss Dynamometry
Bone marrow engraftment	Gastrointestinal symptoms such as esophagitis, mucositis, diarrhea, dysgeusia and xerostomia Percentage of weight loss Dynamometry
1 month after HSCT	Gastrointestinal nutrition-impact symptoms PG-SGA Percentage of weight loss Dynamometry
3 months, 6 months and 1 year after HSCT Outpatient	Gastrointestinal nutrition-impact symptoms PG-SGA Percentage of weight loss Dynamometry

Source: Adapted from Ministério da Saúde. Instituto Nacional de Câncer José Alencar Gomes da Silva (INCA). Consenso Nacional de Nutrição Oncológica. 2ª ed [Internet]. Rio de Janeiro (RJ): INCA; 2015. 186 p [citado 2019 Maio 10]. Disponível em: http://www1.inca.gov.br/inca/Arquivos/Consenso_Nutricao_vol_II_2_ed_2016.pdf^(49)^

NRS-2002: Nutritional Risk Screening; PG-SGA: Patient-Generated Subjective Global Assessment; SGA: Subjective Global Assessment.

HSCT is a highly complex therapy, with major nutritional risks. Thus, it is of utmost importance that nutritional assessment and nutritional interventions be carried out properly, contributing to better clinical outcomes.

## BODY COMPOSITION ASSESSMENT

The body composition assessment, including assessment of muscle mass and peripheral and visceral fat, has shown considerable association with the morbidity and mortality of different diseases, such as cancer.^84-87^

In HSCT, body composition has been studied, and has important correlations with complications and survival.^88-90^ Changes in nutritional status during HSCT serve as a prognostic indicator for these patients.^91-93^

Although most patients are not malnourished at the start of HSCT, low-weight and obese patients have a great risk of early death after HSCT; also, the deterioration in nutritional status during HSCT is also a negative prognostic indicator for these patients.^91^

Both protein-calorie malnutrition and obesity increase the morbidity and mortality risk, days of hospitalization, duration of immunosuppression, and the chance of developing GVHD.^18,91,92,94,95^

The prevalence of obesity in HSCT varies between 10 and 34%,^88,89,93,96^ and it is associated with a higher incidence of GVHD, infections and mortality.^89,96^ A recent study with patients undergoing allogeneic HSCT showed an inverse association between areas of visceral and peripheral fat and disease-free survival.^90^

Also the decreased muscle mass associated, among other factors, to the use of corticosteroids, is correlated with a poorer prognosis in the different types of HSCT.^88^ In allogeneic HSCT, this decrease is associated with a higher prevalence of chronic GVHD and low performance.^88,94,95^

In most studies, body composition assessment in HSCT has been carried out by computed tomography (CT) scan, bone densitometry by whole-body dual-energy X-ray absorptiometry (DXA), and bioelectric impedance (BIA).^88,90,97,98^However, the use of both CT scan and DXA, in this assessment, is associated with ionizing radiation and high cost, not allowing for appropriate patient follow-up,^90,99^and presenting limitations for its performance in obese patients.^99^

In the case of BIA, although free from ionizing radiation, low-cost and allowing for appropriate follow-up, the test has limitations in its use for patients with edema and obese patients, affecting the quality of results.^99^ In HSCT, this method is used to assess body composition, due to its practicality; however, there are limitations, such as hyper-hydration in these patients, which can lead to overestimation of the lean mass.^88,97^

Dualenergy X-ray absorptiometry is not offered in most Brazilian services, due to its high cost and/or unavailability. However, in pediatric patients undergoing allogeneic HSCT, most studies use DXA.^100,101^ We did not find any studies in adults.

Ultrasound (US) is a simple, low-cost, practical and portable method, has emerged as a possible tool for assessment of body composition in hospitalized and obese patients.^99,102,103^ This method has been applied in clinical studies and allows for measurement of muscle thickness, with good correlation with gold-standard methods, such as CT scan or magnetic resonance (MRI).^103-105^ Ultrasound in the elderly allows for assessment of sarcopenia, with results comparable to those of DXA.^106^ Also, it is possible to study the association between quality of muscle mass and strength, assessing the echogeneity, which is based on pixels on the images.^107-110^ There are no studies using this method in HSCT.

One of the routine tests conducted in cancer patients, particularly those eligible for HSCT, is chest computed tomography (CT scan), which has the advantage of providing a precise quantification of lean mass and an exact differentiation between muscle and fat. The review of images, using specific tomographic views, has a good correlation with fat mass and lean mass throughout the body. Between these points, we have the third lumbar vertebra and the fourth thoracic vertebra.^111-114^

In addition to allowing for visualization of fat and lean mass, CT scan also assesses radiodensity, the average radiation attenuation in Hounsfield units which, when low, in some studies, seems to lead to a better prognosis than sarcopenia in hematological tumors.^115,116^

There are few studies in HSCT; however, one study found a larger amount of visceral and subcutaneous fat associated with a shorter disease-free survival.^90^ Since it is included in most pre-HSCT assessment protocols, this could be a promising test for assessment of body composition.

Assessment of body composition should be part of the routine nutritional assessment of patients undergoing HSCT. Each service should select the most appropriate and cost-effective method for their patients.

## BIOCHEMISTRY

The nutritional status diagnosis is essentially clinical. Laboratory; tests are used as an ancillary tool and should not replace clinical evaluation.^117^In patients undergoing HSCT, classic markers of nutritional status, such as lymphocyte count, anergy skin testing, and the presence of nutritional deficiency anemia, are impaired. Also, the interpretation of serum protein levels is also difficult due to the degree of inflammation and potential associated infections, particularly in inpatients.^118^ It is worth noting the importance of sampling nutritional markers before the transplant, in an attempt to reduce the interference of these factors in the previous nutritional assessment.

## PROTEINS USED FOR NUTRITIONAL STATUS ASSESSMENT

In the absence of inflammation (CRP <10mg/L), albumin levels <3.0g/dL indicate a potentially malnourished patient with a poorer prognosis. Although albumin does not adequately correlate with malnutrition in the presence of inflammation, it is still a strong indicator of risk for morbidity and mortality in these cases. Albumin has a half-life of approximately 20 days, which limits its use as an immediate marker of improved nutritional status. Transthyretin, also called prealbumin, has a short half-life (2 days), and is sensitive to any changes in protein synthesis and catabolism. It can be used for PNT monitoring. Retinol-binding protein has the shortest half-life (12 hours) and is a fine marker of protein depletion.^117^

It is noteworthy that screening of said proteins must be carried out concomitantly with sampling of acute-phase proteins. The CRP tests aims to quantify inflammation, helping in the interpretation of the tests at issue.^117^

## INDIRECT ASSESSMENT OF CATABOLISM AND MUSCLE STATUS

Creatinine is formed from creatine, a compound found almost exclusively in muscle tissue. In patients with marked cachexia, creatinine levels may be a marker of muscle loss. In these patients, increased serum creatinine may indicate loss of kidney function, even with creatinine levels within normal ranges. The creatinine height index (CHI), based on creatinine levels in urine, may be used to estimate muscle mass, with an index >60% suggesting severe muscle depletion:^117^

CHI = (24h urine creatinine / 24h urine creatinine estimated for ideal body weight) x 100

Reference values for estimated urine creatinine are^117^23mg/kg ideal weight for men and 18mg/kg ideal weight for women.

The 24-hour urinary urea levels are used to calculate the nitrogen balance, which guides protein replacement therapy, and represents the difference between the nitrogen administered (protein supply) and excreted (skin, feces, urine). Values ≥-5g in 24 hours are considered adequate. Although an interesting tool to assess NT, it has limitations in patients with loss of kidney function and large extrarenal losses, such as diarrhea in patients with intestinal GVHD.^117^

Nitrogen balance = AN (EN) AN = ingested proteins (g in 24 hours) / 6.25EN = UU × 0.5 + (1.2 × (UU × 0.5) + 4

Where:

AN: administered nitrogen

EN: excreted nitrogen

UU: 24-hour urinary urea in g.

## OTHER TESTS

Micronutrient and trace element testing is not a routine in nutritional status assessment. However, severely malnourished patients may have complications associated with their nutritional deficiency. A high level of suspicion for these deficiencies is needed, since clinical signs are discreet, which makes diagnosis difficult, and testing must be guided by clinical suspicion.^117^

Although not traditionally a part of nutritional status assessment, electrolyte testing has a major role in patients undergoing HSCT receiving PNT or at risk for refeeding syndrome. Sodium, potassium, calcium, phosphorus, chloride and magnesium are greatly affected by chemotherapeutic drugs given to these patients, which influences PNT prescription. Marked weight loss or prolonged fasting (>10 days) are risk factors for refeeding syndrome, the hallmark of which is hypophosphatemia. Electrolyte replacement in these patients must be given before feeding is resumed.^117^

Assessment of liver function and glucose metabolism^119^ (preferably via glycated hemoglobin - HbA1c), though not directly related with nutritional status, is essential in these patients. Patients undergoing HSCT have higher risk for metabolic syndrome and diabetes, and the presence of hyperglycemia has direct implications on nutrition prescription and the risk of post-transplant infection. The hepatotoxicity of the drugs used, the use of PNT, and weight gain post-treatment, in addition to prior changes in liver function, are relevant during patient follow-up.

## PHYSICAL EXAMINATION WITH SPECIAL FOCUS ON NUTRITIONAL STATUS

Hematological diseases are frequently associated with changes in the nutritional status. Particularly in patients undergoing HSCT, a physical examination with special focus on nutritional status is essential during the entire process (pre-transplant stage, immediate post-transplant, and late post-transplant periods). Although patient history and physical examination are considered cornerstones for appropriate clinical diagnosis, this aspect is sometimes neglected in daily practice.^120^ Proper conduction of the physical examination by objective assessment is capable of detecting malnutrition or nutritional risk, and assisting^121^ in defining the best type of nutritional support for each patient. When speaking about malnutrition, the term includes both obesity and protein-calorie malnutrition − which are both associated with increased risk in patients undergoing HSCT.^119^

Physical examination is an efficient, low-cost and relatively simple method.^122^ It must be done from head to toe. For a complete physical examination, one must use inspection, palpation, percussion and auscultation techniques. On inspection, the patient must be fully examined, checking for skeletal muscle symmetry,^120,121^ and making sure to ask patients or relatives about any apparent changes in physical conditions and functional alterations observed in recent months, or after the onset of treatment.^121^In patients with mucositis, for instance, examination of the oral cavity may be a determinant of the type of nutritional support. Its clinical manifestations range from soft tissue erythema to the presence of pseudomembranes and erosive lesions, which may cause secondary infection and sepsis. Poor oral hygiene, periodontal disease and tooth decay are risk factors for chemotherapy-mediated oral toxicity.^123^

The appropriate techniques for the physical examination of areas allowing for classification of patients as well-nourished, moderate and severe loss of lean mass and subcutaneous fat, are described in tables 5 and 6. Evaluators must look out for signs of recent weight loss, fluid retention, loss of lean and fat mass, and specific signs of macro and micronutrient deficiencies.^124^

**Table 5:** Lean mass assessment and nutritional status classification as per the physical examination

Areas examined	Appropriate technique	Well-nourished	Moderate loss	Severe loss
Temporalis muscle	Standing in front of the patient, observe the region, then ask patient to turn head side to side and observe the lateral region	Can see/feel well defined muscle Slightly bulged or flat	Slight depression	Hollowing, deep depression
Clavicle bone, pectoris major, deltoid and trapezius muscles	With the patient erect, observe the frontal and dorsal regions Look for prominent bones	Men: bones are not visible Women: bones visible, but not protruding	Men: bones visible Women: bones visible with slight protrusion	Prominent bones
Clavicle bone, acromion process and deltoid muscle	Patient may be sitting or standing, with arms extended alongside the body	Rounded, curves at shoulders and neck	Acromion process may slightly protrude. Sharp shoulders	Shoulder to arm joint square, angular Prominent bones, acromion protrusion very prominent
Scapular bone region, dorsal trapezius and lumbar region	Patient may be standing or sitting, hands straight out, pushing against a solid object	Bones not prominent, no significant depressions	Mild depression, bones may show lightly	Prominent, visible bones, easy to see depression between shoulders, ribs, scapulae and the vertebral spine
Dorsal hand, interosseus muscles	Look at thumb side of hand, ask patient to bring together tip of forefinger and tip of thumb	Muscle bulges, could be flat in some well-nourished adults	Slightly depressed	Significantly depressed
Anterior thigh region, quadriceps muscle	Patient must be sitting and propping leg up on the bed/chair in a 90º angle	Well developed muscle	Mild depression on inner thigh	Clear depression/line on thigh
Patellar region, quadriceps muscle	Patient must be sitting and propping leg up on the bed/chair in a 90º angle	Muscle protrudes, bone not prominent	Slightly protruded bones, less muscle definition around the knee	Prominent bones, little sign of muscle around the knee
Posterior calf region	Patient must be sitting and propping leg up or bending knee, or sitting with legs hanging off edge of bed	Well developed muscle	Muscle not well developed, reasonably shaped, slightly firm on palpation	Minimal to none muscle definition, no firmness on palpation

Source: Adapted from Fischer M, JeVenn A, Hipskind P. Evaluation of muscle and fat loss as diagnostic criteria for malnutrition. Nutr Clin Pract. 2015;30(2):239-48;^(121)^ Academy of Nutrition and Dietetics. Physical Exam – Parameters Useful in the Assessment of Nutritional Status [Internet]. Meridian ID: Academy of Nutrition and Dietetics. Malnutrition Coding in Biesemeier. Nutrition Care Manual. 2013 [cited 2017 Jan 1]. p. 1–3. Available from: http://www.idhca.org/wp-content/uploads/2018/07/SCOLLARD_NFPE-Idaho-Physical_Exam_MN.pdf^(128)^

**Table 6:** Subcutaneous fat assessment and nutritional status classification as per the physical examination

Areas examined	Technique	Well-nourished	Moderate loss	Severe loss
Orbital region	Standing in front of the patient, touch above cheekbone Fluid retention may mask loss	Slightly bulged fat pads	Slightly dark circles, hollow look	Dark, deep circles
Upper arm and triceps	Arm bent in a 90º angle, use your forefinger and thumb to pinch skin and fat	Ample fat tissue between folds of skin	Regular fat tissue between folds of skin	Minimal fat tissue between folds of skin Fingers touch
Thoracic and lumbar region, ribs and iliac crest	Patient may be standing if possible, hands straight out, pushing against a solid object	Chest is full, ribs do not show, slight to no protrusion of the iliac crest	Ribs apparent, depression between them less pronounced, very prominent iliac crest	Depression between ribs very apparent, iliac crest very prominent

Source: Adapted from Fischer M, JeVenn A, Hipskind P. Evaluation of muscle and fat loss as diagnostic criteria for malnutrition. Nutr Clin Pract. 2015;30(2):239-48;^(121)^ Academy of Nutrition and Dietetics. Physical Exam – Parameters Useful in the Assessment of Nutritional Status [Internet]. Meridian ID: Academy of Nutrition and Dietetics. Malnutrition Coding in Biesemeier. Nutrition Care Manual. 2013 [cited 2017 Jan 1]. p. 1–3. Available from: http://www.idhca.org/wp-content/uploads/2018/07/SCOLLARD_NFPE-Idaho-Physical_Exam_MN.pdf^(128)^

The loss of muscle and subcutaneous fat during treatment are often associated with worse food acceptance, and seen as a natural response to the stress caused by the disease.^121^ However, in obese patients or patients with marked edema, fat and muscle loss may be not so simple to assess, which corroborates the importance of a weight loss history.^120^ Loss of subcutaneous fat is observed in areas where fat tissue is normally present. Patients with inflammation, in particular, such as patients subjected to HSCT, loss of subcutaneous fat may be more indolent, even with major catabolism, and become disproportional to the loss of muscle mass.^118^

Muscle assessment must include volume, tonus and functional capacity. In general, superior muscles are more prone to nutrition-related wasting; muscle wasting from inactivity, however, is more associated with inferior muscles.^120^ Muscle mass assessment can be performed through grasping and pinching of the areas observed: starting at the superior body, examining the temporal and orbital regions; the frontal clavicular bone and pectoris major, advancing to the deltoid and frontal/dorsal trapezius, shoulders, acromion process and scapulae, the upper arm and triceps, followed by the thoracic and lumbar regions, the ribs and iliac crest, as well as dorsal hands and interosseous muscles; in the inferior body, the quadriceps, patella and posterior calf should be examined.^121^ Calf circumference is a simple alternative to assess muscle mass, especially in elderly patients. Values under 31cm^3^ are considered abnormal.

Combined evaluation with muscle function is important, due to the high prevalence of sarcopenia in this population.^125^ Special highlight should be given to hand grip strength assessment using a dynamometer, which can be carried out at the bedside and correlated with prognosis in cancer patients.^126^ There are specific tables with cutoff points for sex and age; however, values under 30kg for men and 20kg for women are classified as decreased muscle strength.^127^

Weight gain in these patients, particularly in the immediate post-transplant phase, may be related with fluid build-up, which can range from subcutaneous edema to anasarca. To assess the presence of subcutaneous edema, the sacral region must be examined in bedridden patients and the ankle region in outpatients.^120^ The presence of ascites, pleural or pericardial effusion and anasarca must be recorded in patient progression notes.

## FOOD ACCEPTANCE DURING HEMATOPOIETIC STEM CELL TRANSPLANTATION

Hematopoietic stem cell transplantation-associated clinical changes lead to changes in food intake and eating experience. Taste is a sensory modality involving cognitive and emotional aspects deeply rooted in identity, references associated to pleasure and to a wide range of experiences throughout life.^129^ Conditioning chemotherapy for HSCT is a procedure that can modify the eating experience.^130^

Dietary care must be started pre-transplant and continue during all HSCT phases and modalities, not only to ensure the right supply of nutrients and calories, but also to improve coping with the disease. Dietary restrictions, cultural and religious particularities and diet types (vegetarian, omnivorous, vegan, kosher, among others) must be probed to be considered during hospitalization.

When eating, patients express the difficulties experienced and emotional oscillations occurring since the diagnosis. The conditioning period is permeated by a series of challenges, and the day-to-day is full of symptoms, such as pain, nausea and vomiting, associated with suffering and anguish, which impact different spheres of life and relation with food.^130^ Hospital diets usually have little flexibility to overcome difficulties, *i.e*. strict meal service times and monotonous choices.^131^ Dietitians must understand these issues in an integrated manner and manage nutritional care in a way to overcome these difficulties.

Nutrition management must be clarified and negotiated with patient and caregivers with direct influence on dietary care.^132^ Patient compliance improves as caregivers get more involved and commited to their nutrition. The toxicity inherent to conditioning limits the intake of calories and nutrients, which deserves attention of the team, since it is directly related with treatment outcomes.^133^

## DIETARY MANAGEMENT IN HEMATOPOIETIC STEM CELL TRANSPLANTATION

Dietary management aims to preserve or restore nutritional status, contributing to a better prognosis and reducing toxic effects related to the treatment; attenuate and overcome digestive symptoms; and improve patient experience during hospitalization and treatment. Estimating nutritional requirements at the start of treatment, monitoring food intake, detecting difficulties and promptly managing them are vital roles of nutritional care. The literature shows that food intake during conditioning corresponds to approximately half of the prescribed diet.^131^ Difficulties adapting to the hospital environment, presentation and attractiveness of dishes, and meal times are some of the factors related with low intake.^131,133^

Several complications that occur during hospitalization, especially those deriving from drug toxicity, require specific dietary procedures. Symptoms like nausea, vomiting, hyporexia, diarrhea and mucositis can be circumvented with changes in food offer, diet temperature and consistency management, introduction of enzymes (lactase, alpha galactosidase, among others) into foods during episodes of diarrhea due to enzyme deficiency, use of special supplemental foods and nutrients, among others.^94^

## TASTE

Taste is a complex process of multisensory integration,^134^ that allows for identification of nutrients that should be consumed, and toxins and non-digestable materials that should be avoided.^129,135^ Taste allows us to recognize and distinguish sweetness, saltiness, sourness, bitterness and umami, which are the basic tastes or flavors.

In the oral cavity, taste is created by chemical substances diluted in saliva, which are identified by specific receptors on taste buds.^129^ Recognizing flavors in our mouth also depends on retronasal odor perception. When we exhale, we blow small air streams from the food to the upper back of the mouth, through the nose passage, as we chew or swallow.^135^

Sensory changes are frequent with HSCT and orchestrated by interconnected, internal and external factors, which hinder understanding of the mechanisms involved.

Dysosmia, or impaired odor perception, affects the consumption and preparation of foods.^136^ These conditions may develop and persist even months after conditioning, due to toxicity of the drugs used in treatment.^47^Changes in flavor and odor perception in patients undergoing chemotherapy are present in approximately 70% of cases^47,136^ and may result in significantly decreased food intake and, consequently, decreased calorie consumption. A study has shown that patients with abnormal taste perception eat approximately 430kcal/day less than patients with normal taste perception.^35,136^

In HSCT inpatient units, patients often complain about the smell of foods and how they make them feel nauseated. Unattractive meals can worsen hyporexia. Table 7 shows examples of situations that impact food intake.

**Table 7:** Precautions to be taken in hospital routine to improve eating experience

Ambience	Adequate furniture, attention to routine procedures (smell of cleaning products and at what time of day they are used, closed bathroom doors etc.) during mealtime; keep the room well-ventilated and clean.
Respect of individual characteristics	Ensure patients do not receive foods to which they are averse or cannot eat (refusal, religion, or other reasons). Include the food items negotiated with patients and requested in the routine
Support and accommodation for meals	Ergonomic cutlery and tray table, assisted diet whenever needed, cleanliness and tidiness of the room
Odors and presentation of meals	Avoid strong-smelling foods, use alternatives such as cloche trays with holes to let odors out, or minimize odors by previously opening the tray; avoid plates with divisions and very large portions; avoid unattractive supplement and diet packagings, and exert caution when using fragile disposables during meals
Food restrictions	Avoid unnecessary diet restrictions (such as low-sodium or protein restriction diets, and other restrictions that must be weighed and evaluated)
Use of condiments	OIive oil, butter, dried herbs, lime, among others, can be tested with the patient and then used
Nutritional education and support from the staff	Awareness-raising and explaining the importance and characteristics of the diet to patients and companions can improve adherence and help overcome difficulties; changes made in the diet must be negotiated with patients; the staff must share information to avoid giving different guidance to patients

Source: Calleja Fernández A, Pintor de la Maza B, Vidal Casariego A, Villar Taibo R, López Gómez JJ, Cano Rodríguez I, et al. Food intake and nutritional status influence outcomes in hospitalized hematology-oncology patients. Nutr Hosp. 2015;31(6):2598-605;^(131)^ Gustafsson IB, Öström Å, Johansson J, Mossberg L. The Five Aspects Meal Model: a tool for developing meal services in restaurants. J Foodserv. 2006;17(2):84-93.^(137)^

### Umami taste

Yet to be better explored, the umami taste, which derives from amino acid monosodium glutamate, is an alternative to stimulate taste perception.^135^ It has gained attention because it is one of the last tastes or flavors to be discovered, and is therefore known as the fifth taste, in addition to the other four: sweet, sour, salty and bitter.^138^ Because it activates specific receptors for glutamate and its byproducts, taste-m-GLUR4 (a metabotropic receptor of glutamate) seems to be one of the most preserved tastes during gustatory changes inherent to prolonged exposures to chemotherapy toxicity.^135^

Umami can be composed of, in addition to glutamate, other salts such as disodium guanilate and inosinate, naturally present in foods like cheese, tomato and fish.^138^ It affects the salivary flow, promoting better dilution of the chemical compounds from foods in the oral cavity. In Brazil, a study showed that it is identified, even at low thresholds, by children diagnosed with leukemia and lymphomas undergoing conditioning chemotherapy,^135^ which makes it a possible alternative that should get more attention from those in the field of dietary management. The dose compliant with the NOAEL (Non Observed Adversed Effect Level) was established at 1,600mg/kg of body weight for oral administration − which is quite high, even for big consumers.^139^ The use of monosodium glutamate to season food can be an alternative to be tested in patients with impaired taste perception.

### Sweet, salty, sour and bitter tastes

Altered perception of sweet, salty, bitter or sour tastes is described in a review with 446 subjects on different chemotherapy regimens.^140^However, the diversity of methods, outcomes and chemotherapy protocols does not allow for identification of a taste perception change profile. Studies in patients undergoing allogeneic transplants showed that they have difficulties perceiving the intensity of high and low concentrations of sweetness, and the changes in taste persist for up to 3 years after HSCT, which could be considered a permanent change and a late complication of HSCT.^141^ This has a major impact on dietary management and requires professionals to monitor these changes and find food alternatives to attenuate patients’ disappointment when they cannot recognize the taste of food. There are two possible dietary approaches: resorting to known and appreciated foods and preparations, to encourage consumption of foods that bring positive past references to the patient; and investing in new preparations and foods to avoid the disappointment that comes with an impaired taste perception, which helps improve the eating experience.

## NUTRITIONAL REQUIREMENTS IN HEMATOPOIETIC STEM CELL TRANSPLANTATION

After the nutritional diagnosis, the next step is to calculate nutritional requirements.^49,142^

The energy requirement of patients can be calculated with predictive equations or IC. According to the ASPEN, IC is the recommended method to determine energy requirements in critically-ill cancer patients. When it is not available, predictive equations, such as those of Harris Benedict, Scholfield, and others, can be used. Another fast and widely applicable method to calculate energy requirements is a simple formula that uses calories perkg/current weight or adjusted weight.^40,143^

The nutritional status of HSCT candidates is on its own considered a risk factor, since these patients are already at nutritional risk, due to drug toxicity or to their underlying disease.^49,144^

During HSCT, two distinct situations occur simultaneously: a decreased oral intake of foods and increased metabolic requirements, which affect the patient’s nutritional status.^145,146^

The maintenance of a good nutritional status is of utmost importance throughout the entire HSCT process, and depends on an appropriate supply of nutrients.^145,146^

A patient with a good nutritional status has a high prognostic factor. Thus, nutritional support is indicated before the onset of digestive complications. The changes experienced by these patients affect energy, protein and micronutrient metabolism. The conditioning regimen and transplant complications (sepsis, GVHD, mucositis, diarrhea, among others) lead to a negative nitrogen balance and loss of skeletal muscle mass.^147^

Hematopoietic stem cell transplantation is a procedure that requires high-dose chemotherapy due to its therapeutic aggressiveness, and therefore demands special nutrition care, since patients are at a higher risk of malnutrition before and after transplant. The conditioning regimen has effects on the gastrointestinal tract and immune system, leading to metabolic and nutritional changes. Altered nutritional status before transplant is a negative prognostic factor for patient progression.^147^

Allogeneic HSCT tends to be more aggressive when compared to autologous HSCT; however, virtually all patients had complications, irrespective of the type of transplant, and they all benefited from nutritional support.^147^

In the late post-HSCT, patients may present with several nutritional problems, such as malnutrition, due to insufficient oral intake, or metabolic disorders, such as diabetes, obesity, dyslipidemias and hypertension.^119^

Nutritional counseling must be appropriate to cover patient’s nutritional requirements, respecting their eating habits and food preferences.^49^

Fluid requirements for HSCT patients are based on the recommendations for healthy individuals, *i.e*., 1m/kcal or 35ml/kg/body weight; however, due to dynamic losses and frequent fluid retention, some adjustments may be needed in these calculations.^49,148^

## ENERGY REQUIREMENTS

Hematopoietic stem cell transplantation is a very stressful condition with a high energy requirement due to hypermetabolism and increased catabolism, cytoreductive therapy, infections, multiple organ failure, and tissue repair.^91^

Hematopoietic stem cell transplantation predisposes patients to digestive complications. Aggressive immunosuppression can cause nausea and vomiting, mucositis, impaired taste perception and esophagitis, as well as diarrhea, a common complication that can persist for weeks after HSCT.^147^

These undesirable digestive manifestations experienced by these patients have negative implications on the protein-calorie supply and nutrient absorption, in addition to the increase in energy requirements brought about by the treatment and prolonged hospitalization. The consequence of these factors combined is progressive deterioration of the nutritional status.^149^

According to the *Instituto Nacional de Câncer José Alencar Gomes da Silva* (INCA), determination of the energy value must take into consideration the baseline nutritional status of the patient, metabolic stress, age and weight.^49^

Although energy expenditure varies between autologous and allogeneic transplants, due to intense catabolism, it is generally agreed that energy requirements will range between 130% and 150% of the baseline energy expenditure.^146,147,150^

In most HSCT centers, energy recommendation protocols are similar. Szeluga et al.,^151^ demonstrated that maintaining nitrogen balance at zero required 30 to 50kcal/kg body weight/day in adolescents and adults.^146,151^

According to the Fred Hutchinson Cancer Research Center criteria, in the post-HSCT period (days 30 to 50), energy requirements are higher due to conditioning, fever, infections, GVHD and other metabolic complications.^146,152^

Critically-ill patients are usually in a hypermetabolic state and, therefore, have higher nutritional requirements.^143,153^

Patients subjected to HSCT are at risk of malnutrition due to prior chemotherapy and/or radiation therapy, which lead to undesirable digestive changes, increased energy requirements, and prolonged hospitalization.^149^

Regarding carbohydrate metabolism, glucose intolerance has been reported, caused by administration of cyclosporine and steroids, or due to sepsis. Lipid metabolism abnormalities are less frequent in the first phase of the transplant, however high levels of cholesterol and triglycerides are seen in the late post-transplant period. The lipids offered in the diet must contain long- and mid-chain triglycerides.^147^

In the post-HSCT period, hyperglycemia and abnormal lipids are the most common, and they increase the risk of comorbidities. Glucose must be administered at no more than 5g/kg of body weight/day, and lipids must be used to complete the calorie requirements (20% to 30%). That is why it is important to supply appropriate doses of lipids, to reduce the amount of glucose in patients with hyperglycemia.^119^

Glucose control is important during the post-HSCT period, since hyperglycemia raises the risk of infectious diseases caused by neutropenia, immune cell dysfunctions, cytokine increase and proliferation, muscle and lipid catabolism, in addition to an increased risk of GVHD in allogeneic HSCT, as well as morbidity and mortality.^119^

## PROTEIN REQUIREMENTS

Increased metabolism results from some factors, such as fever, infections, chemotherapy or radiation therapy, leading to tissue destruction.^149^

Protein requirements are estimated to provide the right substrate for tissue repair after cytoreductive therapy, and minimize the loss of lean mass.^146,152^

Most critically-ill patients have proportionally higher protein requirements than energy requirements, due to the fact that protein is the most important macronutrient in tissue repair, immunological support, and maintenance of lean mass.^40,143^

There is a consensus regarding protein requirements in both types of transplant (autologous and allogeneic), which ranges between 1.4 and 1.5g/kg of body weight/day, and can reach up to 2.0g/kg/day) to meet nutritional demands related with the transplant.^149,154^

Table 8 lists the nutritional recommendations for adult patients undergoing HSCT.

**Table 8:** Nutritional recommendations for adult patients undergoing hematopoietic stem cell transplantation (HSCT)

Recommendations	Pre-HSCT	Post-HSCT
Energy	35-50kcal/kg/day	30-50kcal/kg/day
Protein	1.5-2.0g/kg/day	1.5-2.0g/kg/day
Glucose	No recommendation	Up to 5g/kg/day
Lipids	Removal of trans fatty acids from diet Adjustment of fatty acids content: - Saturated: <7-10% of total daily calories based on cardiovascular risk - Monoinsaturated: 15% of the total daily calories - Polyunsaturated: 5-10% of total daily calories	Removal of trans fatty acids from diet Adjustment of fatty acids content: - Saturated: <7-10% of total daily calories based on cardiovascular risk - Monoinsaturated: 15% of the total daily calories - Polyunsaturated 5-10% of total daily calories
Water	35mL/kg/day	35mL/kg/day

Source: Brasil. Ministério da Saúde. Instituto Nacional de Câncer José Alencar Gomes da Silva (INCA). Consenso Nacional de Nutrição Oncológica. 2ª ed [Internet]. Rio de Janeiro (RJ): INCA; 2015. 186 p [citado 2019 Maio 10]. Disponível em: http://www1.inca.gov.br/inca/Arquivos/Consenso_Nutricao_vol_II_2_ed_2016.pdf;^(49)^ Fuji S, Einsele H, Savani BN, Kapp M. Systematic Nutritional Support in Allogeneic Hematopoietic Stem Cell Transplant Recipients. Biol Blood Marrow Transplant. 2015;21(10):1707-13;^(119)^ Franceschini SC, Priore SE, Euclydes MP. Necessidades e recomendações de nutrientes. In: Cuppari L. Guias de Medicina Ambulatorial e Hospitalar - Nutrição - Nutrição Clínica no Adulto. Barueri (SP): Manole; 2005. p. 3-32.;^(148)^ Sociedade Brasileira de Nutrição Parenteral e Enteral. Associação Brasileira de Nutrologia. Projeto Diretrizes - Associação Médica Brasileira e Conselho Federal de Medicina [Internet]. São Paulo: 2011 [citado 2019 Abr 26]. Disponível em: http://www.saudedireta.com.br/docsupload/1331171426terapia_nutricional_no_transplante_de_celula_hematopoietica.pdf;^(149)^ Tvedt TH, Reikvam H, Bruserud Ø. Nutrition in Allogeneic Stem Cell Transplantion--Clinical Guidelines and Immunobiological Aspects. Curr Pharm Biotechnol. 2016;17(1):92-104. Review.;^(154)^ Atualização da diretriz brasileira de dislipidemias e prevenção da aterosclerose – 2017. Arq Bras Cardiol. 2017;109(2 Supl 1):1-76..^(155)^

## MICRONUTRIENTS IN HEMATOPOIETIC STEM CELL TRANSPLANTATION

There are few studies about the use of micronutrients in HSCT, and still the level of recommendation for supplementation is low.^94^ It would not be possible to address all the micronutrients involved in this process and, therefore, here we discuss the most frequently mentioned in the literature: zync, magnesium, and vitamins D and B12.

### Zync

Zync is a microelement which is very important for the body. It is a cofactor of >300 enzymes, responsible for the synthesis of nucleic acids, a structural component of different proteins, and it prevents formation of free radicals and maintains the immune system, among other roles.^156^

Deficiency or low serum levels of zync have been seen in patients with leukemia. This situation has been thoroughly studied and characterized in children around the world, and also in Brazil.^159,160^ Zync deficiency in children with leukemia is so relevant that some studies have proposed its supplementation as an adjuvant treatment.^161^

In older patients undergoing HSCT, the decrease in serum zync, already verified in studies, seems to be related with a greater susceptibility to infections, impaired taste perception, changes in the gastrointestinal system, among other reasons.^156,162^

Clinical manifestations of zync deficiency may be caused by genetic changes (acrodermatitis entheropatica), use of PNT without zync supplementation, alcohol abuse, drugs (penicilamin, thiazides and glucagon), intake of high-protein and phytate-rich grains, malabsorption syndrome, sickle cell anemia, blood loss and excessive sweating (in tropical countries).^156,157^ Specifically in HSCT, zync deficiency is associated with low food intake, use of total parenteral nutrition (TPN), malabsorption and increased requirements for restructuring of the bone marrow.^163^

The symptoms of zync deficiency, such as alopecia, diarrhea, skin rash, failure to thrive, pustular and bullous dermatitis, weight loss, recurring infections, delayed wound healing, decreased lean mass, hypogeusia, dysgeusia, impaired nighttime visual acuity, among others,^156,157,164^can be mistaken for post-HSCT symptoms.^165^

In HSCT, zync deficiency has been correlated with late mortality. During the new hematopoiesis and under inflammatory conditions, zync requirements are even higher. Therefore, keeping serum levels within normal ranges during the HSCT process seems to have an effect on the disease prognosis.^163^

During HSCT, high serum iron can be a negative prognostic factor, due to its build-up in the liver. To correct abnormal levels, iron chelating agents are used, which are not free from side effects. Zync is a potent iron chelator, and its supplementation and the consequent maintenance of higher serum levels, associated with chelating agents given to these patients, seem to improve the results, reducing side effects and, furthermore, reducing liver iron overload.^166,167^

Also, HSCT-associated complications are more frequent in patients with lower serum zync, such as acute gastrointestinal GVHD, fever of longer duration, and higher susceptibility to infections. Therefore, it is suggested that low serum zync can be a risk factor for adverse events during and after HSCT.^163,165^

Although this deficiency is underestimated in clinical practice, some studies have shown a 67% deficiency rate in children, particularly young children, after HSCT. In adults, in turn, this deficiency seems to be more prevalent in the pre-HSCT period.^165^

In the post-HSCT period, zync supplementation has been suggested as a potential therapy, aiding immunosuppresants, but with no side effects.^168^ However, it is not a routine in HSCT centers.

### Magnesium

The use of immunosuppressants may cause hypomagnesemia in patients undergoing HSCT.^94^Nonetheless, there are reports of hypermagnesemia in these patients, which can be associated with the use of laxatives, kidney dysfunction or abnormal intestinal motility.^169,170^

Magnesium supplementation must be given only when a laboratory diagnosis is established, since this micronutrient could be increased or decreased.

### Vitamin D

Vitamin D can reduce the incidence of chronic GVHD and mortality in HSCT. Morbidity and mortality of HSCT are attributed to infection, organ system toxicity, GVHD and recurring diseases.^166^

There are two ways in which the human body produces vitamin D: by endogenous skin synthesis and food sources. Endogenous production depends on ultraviolet B (UVB) rays.^167,168^ In diets, vitamin D is found in non-fortified natural products, such as fatty fish (salmon, sardines, cold liver and oil), or some types of mushrooms (shitake), which have relevant amounts of the two main forms, cholecalciferol and ergocalciferol.^168^

In human skin, cholecalciferol is synthesized from 7-dihydrocholesterol when exposed to sunlight (UVB 290-315). Cholecalciferol is biologically inactive and immediately binds to vitamin-D-binding proteins or albumin. Thus, vitamin D3 is metabolized in the liver, into 25-hydroxyvitamin D3, catalyzed by CYP2R1 and CYP27A1 enzymes. In the kidney, 25-hydroxyvitamin D3 is metabolized into 1,25-dihydroxyvitamin D3 by enzyme 1-a-hydrolase (CYP27B1), which is strictly controlled by parathyroid hormone and fibroblast growth factor 23 (FGF-23).^167,168^

Vitamin D deficiency can lead to osteomalacia, bone pain, muscle weakness, fatigue, increased risk of fracture, and precipitate or exacerbate osteopenia and osteoporosis. It can be caused by reduced skin synthesis, reduced gastrointestinal absorption, and hereditary or acquired metabolic diseases. Also, liver failure and chronic kidney disease can lead to decreased vitamin D synthesis; the use of anticonvulsants, glucocorticoids and antirejection drugs can increase vitamin D catabolism.^171^

The main cause of vitamin D deficiency is inadequate exposure to sunlight. People with darker skin are naturally protected from sun rays and need a 3-to-5-fold longer exposure time to produce the same amount of vitamin D than people with light skin. Also, obesity, malabsorption syndromes, bariatric and other gastrointestinal surgeries, medications, chronic granulomatous diseases, lymphomas and primary hyperparathyroidism are related with a higher risk for this deficiency.^172^

Vitamin D has different roles in the body. One of them is the important part it plays in mineral metabolism and bone health. Vitamin D increases intestinal calcium and phosphate absorption, estimulates osteoclast differentiation, as well as calcium resorption, and promotes bone matrix mineralization.

Vitamin D may also relate with the onset of certain diseases, such as cardiovascular diseases, cancer, autoimmune diseases, type 1 diabetes mellitus, multiple sclerosis and inflammatory bowel disease.^168,173-176^

Vitamin D also regulates host immune response and prevents autoimmunity. Vitamin D inhibits maturation of dendritic cells, polarizes T-cell populations to express Th2 rather than Th1 cytokines, and blocks proliferation of allogeneic T-cells. The vitamin D receptor is expressed in multiple hematopoietic precursors, as well as monocytes, some thymocytes and active B and T lymphocytes. Its action is boosted by the binding between retinoid acid/retinoid X receptor.^168,173-176^

In hematological disease, vitamin D also has an important role in anticancer therapy. Vitamina D analogs can take part in the maturation and differentiation of different cell lines into mature blood cells, particularly in MDS and AML. Another important role of vitamin D may be associated with modulated immune response in HSCT, where vitamin D receptors may impact immune reconstitution after HSCT and reduce the risk of infection, GVHD, and side effects.^168,173-176^

Patients undergoing HSCT may have low levels of 25-hydroxyvitamin D due to less exposure to sunlight, the main cause of vitamin D deficiency.^172^ This deficiency results from long hospital stays, low dietary vitamin D, use of steroids, limited outdoor activities, use of sunscreen and decreased oral intake due to gastrointestinal toxicity of the treatment.^171,177^

Also, gastrointestinal GVHD limits vitamin D absorption, and some drugs administered during GVHD can increase this vitamin’s catabolism, affecting liver and kidney function.

### Vitamin B12

Vitamin B12 is a cofactor of only two enzymes: methionine synthase and L-methylmalonyl-coenzyme A mutase. It is required for the development and initiation of central nervous system myelination.^178^

Its deficiency can cause megaloblastic anemia, neurological dysfunction, glossitis, malabsorption, infertility and thrombosis.^178^

In patients undergoing HSCT, pancreatic changes may occur due to atrophy or insufficiency, which may lead to greater loss of fat in stools, in addition to bacterial hyperproliferation.^179^ Also, the presence of intestinal GVHD may hinder absorption of vitamin B12.^180^

In case of vitamin B12 deficiency due to reduced absorption, oral supplementation may not be sufficient, and parenteral supplementation must be initiated.

### Nutritional complications

Cancer patients are frequently submitted to antineoplastic therapy. During conditioning for HSCT, these drugs are used at high doses. Some of the side effects of the conditioning regimen are nutritional complications due to toxicity of chemotherapeutic agents. They include: mucositis, xerostomia, dysgeusia, dysphagia, odynophagia, nausea, vomiting, constipation, diarrhea, anorexia, anemia, leading to deterioration of the patient’s nutritional status and impacting on treatment response. Oral epithelial cells are the first affected by antineoplastic agents due to their high proliferation rate. The complexity of the oral cavity in these patients will depend on the drugs used, dose, general health status, patient tolerability. Oral complications directly impact on nutritional status, because they contribute to a decreased food intake. Patients receiving individualized nutritional counseling in the pre-transplant stage had a lower rate of side effects and better tolerance to treatment.^181^

### Most common chemotherapeutic agents

Patients undergoing HSCT are potential candidates for malnutrition. One of the reasons is related with toxicity of chemotherapeutic agents used during conditioning, until bone marrow engraftment or recovery, leading to decreased oral intake.^182^

Table 9 presents the main nutrition-related toxic effects of chemotherapeutic agents used in HSCT.

**Table 9:** Nutrition-related toxic effects of the chemotherapeutic agents most commonly used in hematopoietic stem cell transplantation

Drug	Nutrition-related toxic effects
Carboplatin	Leukopenia, anemia, thrombocytopenia, nausea, vomiting, nephrotoxicity, constipation, diarrhea and anorexia.
Cyclophasphamide	Myelosuppression, anorexia, nausea, vomiting, stomatitis, mucositis, colitis, nephrotoxicity, cardiotoxicity and hepatotoxicity
Ifosfamide	Myelosuppression, nausea, vomiting, anorexia, mucositis, nephrotoxicity, cardiotoxicity and hepatotoxicity
Carmustine	Late and cumulative myelosuppression, nausea, vomiting, anorexia, stomatitis and nephrotoxicity
Etoposide	Myelosuppression, diarrhea, vomiting, nausea, anorexia, mucositis and hepatotoxicity

Source: Adapted from Ikemori EH, Oliveira T, Serralheiro IF, Shibuya E. Nutrição em Oncologia. São Paulo: Lemar; 2003.^(182)^

### Xerostomia

Xerostomia is defined as a subjective complaint of dry mouth, which can result from decreased saliva production. The main components of saliva are water, protein and electrolytes. These components improve taste, speech and swallowing, facilitating lubrication, irrigation and protection of mucous membranes of the upper digestive tract. The physiological functions of saliva include antimicrobial and buffer properties that protect the teeth. After the onset of symptoms, patients experience discomfort, such as pain, burning sensation and difficulty swallowing.^182^

Nutritional recommendations to relieve xerostomia are frequent intake of liquids in small quantities; encouraging consumption of mints and lemon drops (assess condition of the oral cavity; acid foods promote salivation); and offering moist diets (preparations with sauces, soups and broths).

### Odynophagia

Odynophagia is caused by esophageal ulcers or erosions, resulting from inflammation of infectious (herpes, candidiasis and HIV) or chemical (drugs, acids and caustics) origin, causing pain when swallowing foods. Strong pain and/or burning sensation in the chest are typical symptoms of odynophagia.^181,183^

Nutritional recommendations to alleviate symptom comprise to provide pain relief before meals; avoid consumption of foods that irritate the oral mucosa (carbonated drinks, acid foods, and dry/hard foods); prefer foods at room temperature, and avoid extreme heat or cold; modify diet consistency according to the level of impairment of the oral mucosa (bland, soft, liquefied and liquid diets); association of oral NT, EN, PNT, according to the patient’s severity and clinical condition.

### Dysgeusia

It is characterized by loss or impairment of the sense of taste, where taste bud function is abnormal, and bitter taste is the first to be affected, followed by sweetness and saltiness.^184^

Nutritional recommendations in this situation include enhancing flavors with the use of condiments and spices; consume citric foods; serve food warm and cold, rather than very hot; assess the need to reduce offer of red meat, as well as iron-rich supplements, since these can worsen the sense of a metallic taste in foods, due to the use of platinum-based chemotherapeutic agents (cisplatin, carboplatin and oxaliplatin).

### Dysphagia

Dysphagia is a change in the swallowing mechanism due to multiple causes, such as neurological and/or structural causes, head and neck trauma, stroke, degenerative neuromuscular diseases, head and neck cancer, toxicity of certain antineoplastic drugs, dementias and encephalopathies. Dysphagia most frequently presents as problems in the oral cavity, pharynx, esophagus or esophagogastric transition. Dysphagia or difficulty swallowing can result in food entering the airways, causing cough, choking/asphyxia, lung problems and aspiration. It may also lead to nutritional *deficits* and dehydration.^182,185,186^

Nutritional recommendations in cases of dysphagia are referring patients for evaluation by a speech and language therapist (SLT), adapting diet consistency and using thickeners for liquids, at the SLT’s discretion.

### Diarrhea

Diarrhea occurs when there is a change in the number of evacuation episodes and consistency of stools, which become liquid. Diarrhea leads to abnormal transport of water and electrolytes, which may cause dehydration. Most often, patients undergoing HSCT have diarrhea due to chemotherapy or radiation therapy toxicity of the intestinal mucosa.^183^

Diarrhea is a common complication in patients on high-dose chemotherapy, and even more common in those undergoing HSCT. *Clostridium difficile* has been frequently identified as the cause of infectious diarrhea at hospitals. Prolonged use of antibiotics may potentiate the risk of diarrhea in patients undergoing HSCT.^187^

The presence of diarrhea can be frequent in any phase of HSCT, warranting investigation, since it could have different causes, ranging from shedding of cells due to mucositis to acute GVHD, and infections caused by bacterial or viral enteropathogens.

Nutritional recommendations in cases of diarrhea comprise controlling the intake of lactose, sucrose, as well as high-fat and naturally laxative foods; limit intake of fiber-rich or laxative foods; increase consumption of liquids for rehydration; and use drinks that promote electrolyte replacement, such as coconut water or isotonics. Also, due to the marked, prolonged immunosuppression expected in these cases, it is advisable that all hygiene-related recommendations be observed during handling and preparation of food intended for these patients, minimizing as much as possible the risk of infectious complications in the gastrointestinal tract.

### Constipation

Constipation does not cause major nutritional losses like diarrhea, but it is a symptom that causes considerable discomfort to patients.^183^

Opioids are opium-derived substances indicated to treat pain and dyspnea, a common picture in patients undergoing HSCT. They are classified according to their chemical and pharmacological nature, as well as receptor-affinity. Opioid receptors are found throughout the gastrointestinal system, with higher concentrations in the gastric antrum and proximal duodenum. Their effects are linked to central and peripheral nervous system receptors. In the genesis of constipation, the action of opioids is related to different mechanisms: increased fluid absorption, inhibition of gastric, biliary and pancreatic secretions; participation in gastrointestinal neural activity, decreased propulsion; and increased segmental contractility. These mechanisms result in reduced intestinal mobility, prolonging contact between the intestinal content and the mucosa, favoring greater water and electrolyte absorption and forming, therefore, dry stools.^188^

As for treating opioid-induced constipation, there is still no clinical evidence on the efficacy of exclusively nutritional interventions, including fiber-rich diets and increased fluid intake (except in cases of dehydration) in regulating intestinal function. Thus, patients and caregivers must be informed, through nutritional education, on the adverse effects of these drugs, as well as on the prophylactic purpose of a laxative diet and adequate fluid intake.^188^

Nutritional recommendations in cases of constipation are to increase fluid intake; include soluble and insoluble fiber modules; include whole-grain foods such as grains, brown rice, whole-grain pasta, wheat bran, oat bran, flaxseed meal, and quinoa flakes; consume legumes (beans, lentils, peas, chickpeas and soybeans); fresh fruit is preferred (ensure proper washing); increase intake of leafy vegetables; and drink juices with laxative properties (papaya, orange and prunes).

### Nausea and vomiting

These are common effects, present in different clinical conditions, particularly in cases of drug toxicity, which are frequent with antineoplastic drugs. These disorders may lead to nutritional *deficits* due to poor food acceptance, as well as dehydration, electrolytic changes, and prolonged hospitalization, which poses imminent risks to patients on prolonged neutropenia. The presence of these symptoms has a negative and quite significant impact on the patient’s nutritional status. Their occurrence and severity vary according to the drug, dose and combinations used.^189,190^

The nutritional recommendations for relief of nausea and vomiting are to adjust diet according to patient tolerance (bland preparations); replace foods that cause discomfort; avoid contact of the patient with the food during preparation, because the smell can exacerbate symptoms; during meals, patients must be in a semirecumbent position (sitting or semi-sitting, with elevation of the head of bed), and chew thoroughly; avoid very sweet and/or fatty foods; prefer cold foods; avoid hot liquids when with nausea; hydrate by increasing fluid intake; maintain adequate oral hygiene; meals should be preferably eaten in a place with good air circulation, avoiding hot, poorly ventilated areas.

### Ginger (*Zingiber officinale*)

Ginger is considered a medicinal plant/spice that, among other therapeutic benefits, has a major role in preventing nausea (antiemetic properties) in different situations, such as in patients undergoing chemotherapy.

The direct relation between consumption of ginger extract and control/reduction of side effects in patients undergoing cancer treatment is under investigation. Studies have shown that 1.5g of ginger powder, divided into three daily doses, is effective in women undergoing chemotherapy for advanced breast cancer.^191^

Studies and reviews have found beneficial results when ginger is used in cancer patients undergoing antineoplastic therapy, reducing side effects, such as nausea and vomiting. However, further studies are warranted in humans to elucidate the real efficacy of this medicinal plant. Thus, it is still not possible to adopt the use of ginger in clinical practice.^192^

### Mucositis

Mucositis is a very common adverse event in patients undergoing high-dose chemotherapy and HSCT.

The lesions are very painful, with difficult clinical management, and their occurrence can limit the duration and/or power of the treatment proposed, and also greatly impact on the quality of life of patients, increasing treatment-related costs.^193^

The injury of mucous barrier, or mucositis, is the tissue damage caused by cancer therapy, particularly high-dose chemotherapy and radiation therapy used in HSCT. From the clinical standpoint, two clinical syndromes associated with mucous barrier injury are recognized: oral mucositis, defined by the presence of erythematous, ulcerative lesions in the oral cavity; and gastrointestinal mucositis, which affects the entire digestive tract and can manifest as anorexia, nausea, vomiting and diarrhea.^194^

In 2003, a study with 599 patients demonstrated that 303 (51%) of those who received chemotherapy for solid tumors or lymphoma developed oral and/or gastrointestinal mucositis. Oral mucositis was found in 22% of 1,236 chemotherapy cycles, while the gastrointestinal form, in 7% of cycles. The two forms concomitantly presented in 8% of cycles. An even greater percentage (approximately 75% to 80%) of patients receiving high-dose chemotherapy before HSCT developed severe mucositis. This was considered the most disabling characteristic of the transplant.^195^

Several American reviews have demonstrated severe mucositis increases the use of hospital resources, such as opioids, antimicrobials and PNT, prolonging hospitalization.^194,196^

The European Group for Blood and Marrow Transplantation (EBMT) carried out a study with 25 centers, in 13 European countries, with the primary purpose of verifying the presence of mucositis in patients undergoing autologous HSCT, who received conditioning regimens with high-dose melphalan or BEAM (carmustine, etoposide, cytarabine and melphalan). The study enrolled 197 patients and concluded that 44% of patients evolved with severe oral mucositis, with a median time to onset of ulcerative oral mucositis on D+11. The highest level of discomfort was reached on D+12, which coincided with the onset of neutrophil engraftment. The duration of the use of opioids was 6.5 days in patients with grade 4 oral mucositis, according to the World Health Organization classification. The use of PNT ranged between 19% in patients without oral mucositis to 59% in patients with grade 4 oral mucositis, and 35% for all patients.^193^

Recent studies have shown that the fundamental mechanism involving mucositis pathogenesis is more complex than just the direct injury to the epithelium. The mechanisms through which chemotherapy and radiation therapy can induce oral mucositis seem to be similar.

The following model, with five stages of pathogenesis, is based on current evidence:

1. Initiation: radiation therapy and/or cheotherapy induce cell damage, which results in death of epithelial cells. It is believed that the production and release of free radicals also play a role in initial injury in mucositis.
2. Signalling: additionally to direct tissue damage, free radicals activate secondary messengers, which transmit signals from cell surface receptors to the inside of cells, leading to release of inflammatory cytokines, and causing tissue damage and cell death.
3. Amplification: the release of tumor necrosis factor alpha (TNF-α) produced mainly by macrophages, causes damage to mucosa cells, and also activates molecular pathways that amplify this mucosal injury.
4. Ulceration: there is significant infiltrate of inflammatory cells associated with ulceration of the mucosa, based, in part, on metabolic byproducts of the microbiome colonizing the oropharynx. This secondary infection is also responsible for the production of inflammatory cytokines.
5. Healing: this phase is characterized by epithelial proliferation and cell and tissue differentiation, restoring the integrity of the epithelium.^194^

Clinical manifestations of mucositis include signs and symptoms of inflammation, ranging from discreet erythema, edema and discomfort, to disabling pain and ulcerations. These changes interfere with the patient’s activities of daily living, such as speaking and eating. They can hinder proper nutrition or, in some cases, eating can become impossible.^195^ Dehydration, malnutrition and infections and, consequently, decreased overall survival, have been repeatedly associated with mucositis.^197^

There are three main classifications of mucositis severity, presented in table 10. Yet, the most commonly used oral mucositis classification was proposed by the WHO.

**Table 10:** Comparing degrees of severity of oral mucositis

Grade	0	1	2	3	4
WHO	Nothing	Discreet pain±erythema	Erythema, ulcer; patient is able to swallow solids	Ulcers with extensive erythema Patient cannot swallow solids	No kind of food is possible
RTOG	Nothing	Mucosal erythema	Plaques <1.5cm	Confluent plaques >1.5cm	Necrosis or deep ulceration
WCCNR	No lesions Color: pinkish No bleeding	Lesions: 1-4 Color: slightly reddish No bleeding	Lesions: >4 Color: moderately red Spontaneous bleedings	Coalescent lesions. Color: vivid red Spontaneous bleedings	NA

Source: Niscola P, Romani C, Cupelli L, Scaramucci L, Tendas A, Dentamaro T, Amadori S, de Fabritiis P. Mucositis in patients with hematologic malignancies: na overview. Haematologica. 2007 Feb;92(2):222-31. Review.^(198)^

WHO: World Health Organization; RTOG: Radiation Therapy Oncology Group; WCCNR: Western Consortium for Cancer Nursing Research; NA: not applicable.

There is no consensus regarding prevention or even management of oral mucositis. Different measures have been used to prevent severe mucositis. Good oral hygiene, regular dental checks – including prevention and treatment of cavities and gingivitis – and comprehensive guidance to patients are important measures.

Cryotherapy seems to have an important role in preventing mucositis from high-dose melphalan.^198^ Its purpose is to promote vasoconstriction in the oral cavity and, thus, reduce toxicity from the drug administered. Cryotherapy has been described in the literature due to its low cost, absence of side effects, and influence on the outcomes of the complication at issue.^199^ It is recommended to offer ice 30 minutes before initiating the drug infusion, until administration is completed. Caution is recommended in respect to the quality of water for production of the ice cubes.^199^ Since patients will receive the drug over the course of many hours, and have to simultaneously keep the ice in their mouth, ice prepared with nutritional supplements, fruit juice and infusions has been an alternative for cryotherapy, and could increase the energy and nutrients offered to patients.^200^

Antibacterial prophylaxis, although adopted in recipients of different types of transplant, has not been not proven effective in reducing microorganisms present on the surface of the oral mucosa, which have a major role in the onset of mucositis.^201^

Likewise, the use of chlorhexidine and stimulators of salivary secretion, such as pilocarpine, has not been proven effective in preventing severe mucositis.

## TREATMENTS

### Palliative treatment

Systemic analgesia with morphine in infusion pump is the treatment of choice for oral mucositis-related pain, in both adults and children. The use of transdermal fentanyl for this purpose has also been published, but its efficacy is yet to be confirmed.^202^

### Low-intensity laser therapy

The first studies of laser therapy for oral mucositis have shown reduction of inflammation and pain, when compared to groups not subjected to this therapy.^203^

A Brazilian, retrospective study with 43 patients undergoing allogeneic transplants between 2004 and 2008, comparing two groups – one receiving dental care and low-intensity laser therapy, and a second group with no interventions – found that these procedures can reduce the extent and severity of oral mucositis.

Another study showed that the use of laser therapy and dental care reduced the morbidity of oral mucositis, also reducing the costs of hospitalization.^204^

### Keratinocyte growth factor (palifermin)

Palifermin is a mytogenic agent for fibroblasts, keratinocytes and endothelial cells, which increases mucosal thickness and also attenuates the effects of TNF-α, reducing the typical inflammation found in mucositis. Clinical trials have demonstrated a decrease in the incidence and duration of severe oral mucositis (grades 3 and 4).^205^

### Nutritional therapy in mucositis

NT in cancer patients aims to minimize weight loss and prevent specific nutritional deficiencies. Patients with mucositis also have difficulty swallowing and, sometimes, keeping food in their mouth. Diarrhea may also be present. In these cases, the NT of choice is other than oral and must not exacerbate diarrhea.

In mild mucositis, while oral feeding is still possible, it is important to change the consistency of the diet. In addition, foods that do not irritate the mucosa must be used (reduced acids, spices and salt), as well as foods with a soft texture.

Gastrointestinal mucositis causes damage to crypt cells, decreases production of digestive enzymes, and transient intolerance to lactose can occur. Therefore, a dairy-light diet may be required.

For patients unable to meet their nutritional targets, enteral nutrition (EN) or PNT must be considered. It is possible to safely insert an enteral tube in patients with more severe mucositis. Parenteral nutrition therapy may be indicated for patients unable to be fed orally or enterally due to ileus, incoercible vomiting, or obstructive conditions. Patients with severe mucositis, although they are still candidates for EN, may have additional benefits with PNT, especially for prolonged periods.

Glutamine supplementation was, for a long time, used as part of the management of gastrointestinal mucositis, but recent studies have shown conflicting results regarding its benefits.^206^

The typical lesions of oral mucositis are, by and large, very painful, and may compromise the nutrition and oral hygiene of patients, in addition to increasing the risk of local and systemic infection. Due to its complex and multifactorial pathogenesis, the treatment of mucositis consists of palliative care, such as pain management and oral hygiene, as well as NT, which must be a multidisciplinary decision.^196^

## ANOREXIA

Anorexia can occur due to the disease process, metabolic disorder, side effects of treatment, or even depression, and is common in cancer patients, particularly in more advanced disease states. It can also be related with the effects of nausea and vomiting.^49,207^

Anorexia is defined as early satiety and/or loss of appetite, and is classified as a biopsychosocial phenomenon, present in approximately 40% of cancer patients; its etiology is still under debate.^207,208^

Both hormonal factors and cytokine activity are involved in appetite regulation. Neuropeptide Y and ghrelin are orexigenic hormones, and the first was found at lower levels in animals with tumors. Melanocortin is attenuated in the presence of cancer, and has anorexigenic action. Some cytokines, such as TNF-α, interleukins 1 and 6, and interferon gamma, are released by tumors and act to reduce food intake.^207,209^

Anorexia is not the only symptom involved in the weight loss of cancer patients, although it is relevant in the context of cachexia.^208,210^

It is the most frequent cause of insufficient intake after conditioning regimens, and early satiety and delayed gastric emptying are the main factors contributing to reduced hunger. The appropriate nutritional interventions advocate smaller meals, eating slowly and drinking liquids only between main meals. Other factors impacting appetite include impaired sense of taste and emotional problems, such as anxiety and depression − frequently present in transplant patients, mostly due to prolonged hospitalization.^211^

Anorexia usually occurs between the second and the third weeks of the transplant, mostly caused by the conditioning regimen.^212^ Oral intake is heavily affected during the transplant and remains low for up to 3 weeks after treatment.^213^

A study investigated 147 transplanted patients, and decreased oral intake was observed in 92%, with a mean calorie intake of 3% of baseline energy requirements of these patients. This marked decrease in oral intake took place predominantly between days 10 and 12 of the conditioning regimen.^213^

Another study observed that, in 69 patients followed-up in the post-transplant period, 66% had feeding difficulties on day 50 after the bone marrow infusion, and anorexia was one of the reasons for reduced oral intake. This loss of appetite regressed over time after treatment, but was still prevalent in 16% of patients on day 200 after infusion.^95^

Some studies have investigated the prevalence of malnutrition and its correlation with the presence of side effects in cancer patients on adjuvant or neoadjuvant therapy. A recent multicenter study with 65 hospitals and 561 patients on adjuvant therapy showed that 90.7% had lost weight. The same study found that 96.4% had nutritional complications such as anorexia (70.9%), gastrointestinal disorders (32.6%), dysgeusia (40.5%) and dysphagia, among others.^49^

Since toxicity effects lead to decreased food intake for extended periods, this makes it even more important to monitor the nutritional status of patients undergoing transplant.^95^

In the nutritional approach to anorexia,^49^ educating patients on the need to eat; eating smaller, more frequent meals; increasing calorie and protein content in meals; increasing consumption of high-calorie snacks; increasing consumption of better accepted foods; adding high-calorie, high-protein supplements; and choosing higher-calorie drinks are important points to be observed.

## WEIGHT LOSS

Hematopoietic stem cell transplantation is now indicated in different hematological and high-morbidity, non-hematological diseases. High-dose cytotoxic chemotherapy, with or without total body irradiation, results in hypercatabolism, breakdown of the gastrointestinal mucosal barrier, and immunosuppression. Gastrointestinal and infectious complications from conditioning are frequent, impairing the nutritional status and contributing to high morbidity and mortality. In addition, for patients already in a catabolic state, the increased metabolic demand associated with treatment further aggravates the problem.^214^

A study looked into the impact of patient weight on transplant outcomes. When compared with patients close to their ideal weight, patients with low weight had worse results after allogeneic HSCT, and this was probably related with the aggressive pre-transplant therapy. Also, worse results can reflect differences in drug distribution volumes and an effect of weight on pharmacokinetics.^215^

Patients scheduled to undergo HSCT are at higher risk for malnutrition related with prior chemotherapy and/or radiation therapy, due to side effects − many of which involve the gastrointestinal tract, leading to increased energy requirements and prolonged hospitalization.

Hematopoietic stem cell transplantation has multiple side effects, such as anorexia, diarrhea, mucositis and dysgeusia, which affect food acceptance and, as a consequence, deteriorate patients’ nutritional status.^95,216^ A significant part of patients undergoing transplant develop mucositis, with considerable difficulty maintaining adequate calorie intake, which is even worse in patients with acute GVHD affecting the digestive tract.^217,218^

Nutritional status is an important prognostic factor in patients undergoing HSCT. There are three important periods for patients undergoing HSCT: the initial period, which comprises the conditioning phase, and the cytoreductive phase, causing extensive tissue damage. The second period, right after the transplant, is characterized by intense pancytopenia, followed by repair of damaged tissues. The third period, when the implantation of transplanted cells takes place, is when infectious complications can occur.^219^

In a retrospective study, morbidity and mortality were higher among patients with lymphoma undergoing autologous HSCT when these patients were malnourished at admission, in comparison to those with normal weight.^220^

All patients undergoing HSCT are at nutritional risk, mainly due to adverse effects from prior treatment. Moreover, half of the patients undergoing HSCT have impaired nutritional status for up to one year after transplant, and weight loss and nutritional complications are more frequent in patients submitted to allogeneic HSCT.^221^

Another study also found weight loss after autologous and allogeneic transplant. The authors found that weight loss persisted for up to 75 days after transplant of 118 study subjects, even for those who did not complete the study.^95^

A survey conducted in a cancer institution in the state of São Paulo, in 2016, studied 123 autologous and allogeneic transplants, and found that the mean weight at pre-conditioning was 75.6kg, and the mean weight at post-conditioning was 73.8kg. Of the total patients, 83 lost weight during the transplant, with an average weight loss of 3.2kg.

Some studies have assessed the impact of nutritional status on patients undergoing HSCT. In a study for the ASBMT, which investigated the impact of nutritional status in 1,087 patients treated with high-dose melphalan and undergoing autologous HSCT with a diagnosis of multiple myeloma, patients were sorted based on BMI as normal, overweight, obese or severely obese. There was no overall effect of BMI on progression-free survival, overall survival, disease progression, or relapse-free mortality.^19,222^

The pre-HSCT nutritional status is related with the post-HSCT clinical progression, directly affecting the time to engraftment. Due to the intense toxicity to which these patients are subjected, the risk for malnutrition in the pre- and post-transplant phases must be monitored, as well as complications related to nutritional status, and the consequent decrease in food intake.^214^

Patients with a nutritional diagnosis of malnutrition and obesity are at higher risk of death in the immediate post-HSCT period, and patients with major muscle mass depletion have more clinical complications and longer lengths of stay.^88^

Malnutrition, very often characterized by weight loss, deteriorates patient prognosis and survival.^92,133^ A study investigated the length of stay according to patients’ nutritional status. Patients classified as well-nourished, as per the PG-SGA, had a mean length of stay of 16.9±6.3 days, while patients classified as at risk for malnutrition, or patients with moderate and severe malnutrition, had a mean length of stay of 23.9±9.9 days, with a significant difference between the groups (p=0.002).^92^

Irrespective of whether the current food intake and nutritional status are maintained, patients scheduled to undergo HSCT require individualized nutritional monitoring, aiming to offer and educate on the appropriate NT for each phase of the transplant, minimizing involution of the nutritional status and future nutritional complications. Transplanted patients present with unintentional weight loss, regardless of the type of HSCT to which they are submitted to.^223^

## ORAL DIET

### Nutritional supplements

Nutritional supplements are one of the alternatives when patients are not consuming the calories and nutrients required to cover their nutritional demands,^224^ and to offer nutrients for therapeutic purposes.

Oral nutritional supplements are defined as mixtures containing nutrients for oral ingestion, and can be purchased as ready-to-consume formulations, prepared with fresh foods, and industrially processed; they can also be purchased based on a combination, in order to supply calories and nutrients in a concentrated form.^47,224^Table 11 presents some of the formulations available in the market.

**Table 11:** Characteristics of nutritional supplements available in Brazil

Description of of formulations	Energy density (Kcal/mL)	Osmolality (mOsm/L)	Protein (g)/100 Kcal	Additional characteristics	Brand names
Standard formulations					
Powder	1.0	350-700	3.7-7.5	Flavors: vanilla, chocolate, strawberry, banana. Contains sucrose and lactose	Ensure^®^, Nutren^®^ Active, Sustare^®^, Sustain^®^, Sustacal^®^, Sustenlac^®^ and Sustevit^®^
Liquid, high-calorie, flavored (200-250mL)	1.25-1.5	355-749	4-10	Flavors: vanilla, chocolate, strawberry, banana, grape, pineapple and apple. Contains sucrose and lactose. May contain beta-hydroxy-beta-calcium methylbutyrate	EnergyZip^®^, Nutridrink Protein^®^, Ensure Protein^®^, Ensure Plus^®^, Ensure Plus Advance^®^, Fresubin^®^ Energy Drink, Fresubin^®^ Protein Energy Drink and Fresubin^®^ Jucy Drink
Liquid, high-calorie, flavored and reduced volumes (125-150mL)	1.5-2.4	385-730	3.7-10	Flavors: vanilla, chocolate, strawberry, cappuccino, lime/lemon, peach with ginger, and tropical fruits. May contain sucrose and lactose. May contain EPA	Nutridrink Compact^®^, Forticare^®^ and Fresubin^®^ Lipid Drink
Unflavored	1.0-1.5	400	4-11	May contain sucrose and/or lactose	NUTREN^®^ Sênior
Liquid or semi-liquid, high-calorie	2.0	359-720	4.4-12.5	May contain sucrose. Used in soft diets.	Nutren^®^ 2.0^®^ and Fresubin^®^ 2 kcal Crème
Sucrose/lactose-free formulations					
Powder	1.0	350-470	4.9-6	Vanilla	Glucerna SR^®^ and Novasource^®^ GC
Unflavored	1.5	365-390	5-6.2	Unflavored	Nutridrink MAX^®^ and IMMAX^®^
Liquid (200-250mL)	1.0	334-470	4.3-6	Vanilla, strawberry, and chocolate	Glucerna SR^®^, Diamax^®^ and Diasip^®^, Novasource GC
Liquid, high-calorie	1.5	614	5	Vanilla	Glucerna 1.5kcal^®^

EPA: eicosapentaenoic acid (omega-3). Search conducted on manufacturers’ websites in June 2019.

Prolonged, repetitive use of the same supplements may hinder adherence and reduce tolerance to this strategy. Special caution should be taken with high-osmolar-concentration formulations, when diarrhea is present, and with nutrients that can aggravate symptoms or irritate the mucosa, such as fibers, mono- and disaccharides.^225^ Options with higher energy density are interesting due to their greater calorie supply, however they usually have this limitation. Some strategies, such as using flavorless supplements, alternating options, or even mixing these formulations with foods may improve adherence,^224^ as well as resorting to supplements compounded with foods, which offer greater diversity of flavors and textures.

### Modular supplements

Modular supplements can be an interesting option to improve the consumption of a nutrient alone, such as to adjust protein intake.^224^ They are interesting in dietary management, since they can be used in preparations with foods that are better accepted by or more familiar to patients.

### Carbohydrate module

Usually based on maltodextrin, sucrose or sucrose polymer. Powdered, and used to increase energy densit,y without significantly altering the flavor of the final preparation.

### Lipid module

Based on middle-chain triglycerides, lipid modules are used for their better absorption, their offer of essential fatty acids, or both. Mostly found in liquid formulations, they are also used to increase the energy density of preparations.

### Protein module

Usually based on whey proteins, protein modules may contain essential amino acids, branched chain amino acids, protein hydrolysate, calcium caseinate etc. They are used to increase the protein density of preparations without changing texture or volume. Mostly found in powdered form.

### Supplements with aminoacids glutamine and beta-hydroxy-beta-methylbutyrate

Beta-hydroxy-beta-methylbutyrate (HMB) is a leucine metabolite, which has been used in studies on sarcopenia, with more significant results in young patients than in the elderly, as an alternative for restoring protein synthesis.^47^ Patients under treatment for HSCT, using anthracyclines and corticosteroids, which contribute to accentuate protein catabolism, may have intense muscle fatigue.

The role of glutamine (conditionally essential amino acid) and its benefits in patients undergoing HSCT are not yet well established. Its oral administration has been indicated for symptoms such as mucositis and associated odynophagia; however, studies do not provide conclusive results due to different methodologies.^47,226^

### Probiotic supplements

According to the WHO, probiotics are living organisms that, at the right amount, can promote health and benefits to their hosts.^227^ In related publications, there is no evidence of any pharmacological action of probiotics in the host, however a symbiotic effect has been verified in experimental studies, by comparing parameters such as weight of organs, cardiac output and immune response of experimental germ-free animals.^227^

It is known that patients undergoing HSCT have intense dysbiosis, frequently associated with severe diarrhea, GVHD and infection. The change in the percentage of beneficial bacteria is even more marked with the use of antibiotics, which are frequently given to transplant patients due to the intense immunosuppression to which they are subjected. However, regarding probiotic supplementation in patients undergoing HSCT, few but promising studies have shown that probiotic supplementation, in addition to reestablishing intestinal dysbiosis, is capable of preventing some post-HSCT complications, such as GVHD.^228^ Some findings argued that supplements containing *Lactobacillus* may prevent the proliferation of *Enterococcus* in the gut.^228^ Feared adverse events, such as bacteremia and bacterial translocation, have not been found in the study by Ladas et al.,^228^ who administered *Lactobacillus plantarum* to children undergoing HSCT.^228^

The main microorganisms commercialy available in Brazil are *Lactobacillus acidophilus, Lactobacillus casei shirota*, *Lactobacillus casei* variety *rhamnosus*, *Lactobacillus casei* variety *defensis*, *Lactobacillus delbrueckii* subsp. *bulgaricus*, *Lactobacillus paracasei*, *Lactococcus lactis*, *Bifidobacterium bifidum*, *Bifidobacterium lactis*, *Bifidobacterium longum*, *Bifidobacterium animalis*, *Enterococcus faecium* and *Streptococcus salivarius* subsp. *thermophillus*. The minimum quantity to be considered a probiotic is 10^8^ to 10^10^colony forming units (CFU) per portion, and they can be sold in capsules, powdered, freeze-dried or in yoghurts.^229^ There is no consensus in the literature about which dose to use and at which point during HSCT this supplemention should be initiated; although the idea seems promising, further clinical studies are needed to establish the safety and efficacy in patients undergoing HSCT.

### Herbal supplements

The integrative approach, which combines knowledge, practices and natural resources with conventional treatments, has been well accepted by health professionals, allowing for investigation of new alternatives to relieve symptoms, stimulate the appetite and help alleviate anxiety.^47^

It is always necessary to reinforce to patients and family members that they must inform the care team about any herbal supplements or other natural substances they may be on.^230^ Some infusions, herbs and compounds may contain substances that interfere with the metabolism of some drugs, affecting their action.^230^ For instance, *Hypericum perforatum* (Saint John’s wort), popularly recommended for depressive conditions, may interfere with imatinib mesylate (Glivec^®^).^230^*Camellia sinensis* (green tea or matcha), known for its antioxidant and thermogenic properties, interferes with bortezomib (Velcade^®^).^230^

### Nutritional supplements added to foods

The use of supplements with foods, combined in such a way as to offer calories and specific nutrients, with different temperatures, consistencies and flavors, may be an option to meet nutritional demands during treatment. These alternatives involve food preparations with different presentations and can stimulate food intake and nutritional demands in periods of hyporexia. They can be formulated as needed.

### Diet for patients with neutropenia

Neutropenia is defined as a neutrophil count under 500/µL or lower than 1.000/µL, expected to drop to 500/µL within two days. In these situations, some prophylactic measures are adopted to control the risk of infection.^231^ However, there is no consensus in the literature about the benefits of using a neutropenic diet. Its use was instituted in the 1980’s to prevent digestive tract infections, and its main characteristic was restricting foods that could act as vectors of pathogenic microorganisms.^47,221^ Until 2006, the literature had studies with diets restricting consumption of raw vegetables, fresh and sun-dried fruit, nuts, yoghurts, eggs and undercooked meats. Studies comparing diets with these restrictions with conventional diets, with properly washed foods, showed that said restrictions did not protect neutropenic patients and, in some cases, the number of complications was higher for the more restrictive diets.^232^

According to the ESPEN consensus, due to the difficulty comparing studies with different methodologies and divergent concepts, including in respect to the neutropenic condition, which warrants these approaches, the use of these diets is not supported in the scientific literature.^47^The guidance has been pointing to an opposite direction: instead of restrictive diets, the focus has been on the quality of sanitation procedures, *i.e*., allowing more flexible diets, provided the right precautions are taken when purchasing, storing and preparing foods.^49,232^

For sanitization of fruit and vegetables, it is safe to use chlorine-based solutions, such as the hypochloride solutions intended for this purpose, with 200 ppm of active chlorine, which ensures great antimicrobial power with no corrosive action.^233^ As for residues, the amount ingested is much lower than tolerated, even considering the intake of washed, raw foods, several times a day. After washing, foods can be rinsed with treated water.^233^

The recommendations about the precautions adopted in this sense are those used for the general population, as presented in table 12.

**Table 12:** Suggested procedures for eating and preparing foods

Food	Washing/preparation	Sanitizing solution
Fruit and raw vegetables	Wash in running water and remove visible dirt	Soak for 15 minutes in: 1 tablespoon of hypochlorite solution for 1L of water*
Eggs and meat (poultry and fish)	Always after cooking at 74ºC	Reach 74°C at the center (juices and inner part must be clear and not pinkish or reddish, eggs with yolk cooked hard, and fish must be opaque and break easily into flakes)
Milk	Use pasteurized or UHT milk	Up to 3 days in the refrigerator once opened
Cheeses	Do not consume white (brie and camembert) and blue (gorgonzola) mold cheeses	Keep the cheese in a closed container in the fridge, and consume within up to 5 days once opened (including cheese spreads)
Yoghurts	Do not consume yoghurt with added probiotics	Keep refrigerated and consume within 48 hours once opened
Juice and honey	Consume pausteurized versions	Up to 3 days in the refrigerator once opened
Deli meats (ham and turkey cold cut, etc.)	Prefer vacuum packed products	Up to 3 days in the refrigerator once opened

Source: Adapted from Silva Junior EA, editor. Manual de controle higiênico sanitário em serviços de alimentação. São Paulo: Varela; 2007. p. 239-66.^(233)^

* Maximum safe daily consumption of chlorine: 5,100,000µcg. Average intake in a meal with sanitized vegetables: 7,000µcg.

Water is another important item that is worth discussing, considering aspects related with water treatment, as well as sales and distribution channels. The definition of treated water is water from the water supply system, filtered or boiled for at least 2 minutes, or filtered and chlorinated.^233^ There are national programs under the Ministry of Health, such as the (SISÁGUA - *Sistema de Informação de Vigilância da Qualidade da Água para Consumo Humano*) which monitor the quality of water for human consumption, and must be periodically consulted by healthcare professionals for better understanding of recommendations.^234^ In cities with no watter supply network, it is recommended to add one drop of sodium hypochlorite for each liter of water consumed, or boil the water in a clean pan.^235,236^

As for bottled spring water, a study has shown that fungal contamination may occur during the bottling and/or storage processes, therefore, this alternative is not necessarily safe.^235^

Furthermore, one should pay attention to the cleaning of domestic water tanks every 6 months, as well as make sure to know the origin and the way water containers were stored and transported. These procedures must be explained to patients to guide them on the best options to use high chemical- and microbiological-quality water.^234^

Table 13 presents the main recommendations for patients with neutropenia.

**Table 13:** General recommendations for patients with neutropenia

Purchasing foods	Handling foods and preparing meals	Eating out
Check the expiration date of products	Wash hands before handling foods, before meals, and after using the restroom*	Check the hygiene conditions of the place where you plan to eat. Avoid places where the food is exposed and resting on countertops, or where there is movement of people without proper protection
Buy pasteurized milk and dairy products, fruit juice and honey	Use separate cutting boards to prepare beef, fish or chicken. Prefer boards made of glass	Use sauces, condiments, salt and sugar only in individual sachets
Buy intact fruit and vegetables, and avoid those with visible damage	Keep the refrigerator temperature below 5ºC	Observe if employees who handle food are wearing hairnets
Do not taste foods in supermarkets and points of sale	Defrost meat in the microwave. Turn the turntable at least twice to defrost uniformly	Check if restrooms are clean and if soap is available for washing hands
Avoid foods exposed on countertops	Never leave perishable products outside the refrigerator for more than 2 hours	Avoid drinks with ice of unknown origin
Follow the right order while grocery shopping: first, non-perishable foods, then frozen perishables and, lastly, refrigerated perishable items	Use only treated water for preparing foods and clean the surface before you start	
Check if there is water on the floor by meat counters, freezers or refrigerators: this may indicate that the equipment was off for some time	Discard leftovers after 3 days in the refrigerator	
When transporting foods, avoid warm locations	Cook foods thoroughly. Pay special attention to larger portions and thicker meat cuts. When heating pre-prepared food, check if the temperature is the same in the inner part of the product	
Check if the facility if clean and tidy	Discard all foods that have expired (also consider the opening date)	

Source: Agência Nacional de Vigilância Sanitária (ANVISA). Guia de alimentos e vigilância sanitária [Internet]. Brasília (DF): ANVISA; 2004 [citado 2017 Mar 18]. Disponível em: http://portal.anvisa.gov.br/resultado-de-busca?p_p_id=101&p_p_lifecycle=0&p_p_state=maximized&p_p_mode=view&p_p_col_id=column-1&p_p_col_count=1&_101_struts_action=%2Fasset_publisher%2Fview_content&_101_assetEntryId=395967&_101_type=document;^(236)^

Fox N, Freifeld AG. The neutropenic diet reviewed: moving toward a safe food handling approach. Oncology (Williston Park). 2012;26(6):572-5.^(237)^

* Associated with the use of alcohol gel.

### Parenteral nutrition

Nutritional Therapy in HSCT has the purpose of maintaining and recovering nutritional status, avoiding or reducing nutritional *deficits* resulting from chemotherapy and/or radiation therapy, minimizing the effects of the conditioning regimen, and providing appropriate substrate for recovery of the hematopoietic and immune systems.

Patients undergoing HSCT can be considered as at nutritional risk, due to reduced calorie supply, impaired nutrient absorption, and increased metabolic demands, which may compromise the nutritional status of these patients throughout the process.^12,216,238,239^Thus, these patients are prone to developing multiple metabolic disorders of variable severity − mostly during the immediate post-transplant period. The main causes include adverse effects from the conditioning regimen, immunosuppressants and PNT.^240^

Adverse effects of chemotherapy and radiation therapy predominantly affect the gastrointestinal tract and the immune system.^238^ Therefore, in addition to nausea and vomiting, severe mucositis frequently occurs, associated with intense odynophagia, abdominal pain, diarrhea, and infections.^147^ These factors further impair oral tolerability, contributing to aggravation of weight loss, which can persist for up to 40 days after admission for the transplant.^241^ As a consequence, malnutrition can rapidly occur while the patients is hospitalized for HSCT.

Regarding the type of NT to be used, data suggest a trend towards fewer complications during the use of EN when compared to PNT in HSCT, particularly for infectious complications and propensity for more severe forms of intestinal GVHD.^242^ However, a study with patients requiring admission to the intensive care unit (ICU) did not show any association of poorer outcomes in the group receiving PNT.^243,244^

Parenteral nutrition therapy has been shown to be safer and more effective in maintaining and recovering nutritional status when compared to patients on ENT, with gastrointestinal intolerance and severe mucositis. Cost-effectiveness assessments based on definitive outcome indicators have influenced the clinical practice of delaying PNT initiation for patients at normal weight undergoing autologous HSCT, in contrast to those undergoing allogeneic HSCT, since the duration of food intolerance in the former is shorter.^40,245,246^

### Indications

Parenteral nutrition therapy must be initiated at the pre-transplant phase in all patients eligible for treatment of their underlying disease, presenting significant weight loss with clinical repercussion. Underweight patients (BMI under 18.5kg/m^2^) are candidates for early PNT when oral intake falls below 50% within the first 3 days after infusion. Most of the time, patients candidate for HSCT arrive with good enough nutritional status, and must initiate PNT when gastrointestinal toxicity associated with grade III or IV mucositis occurs, with inadequate food intake for the past 10 days with no expected improvements, and when enteral nutrition has been excluded.^147,232^

Parenteral nutrition therapy with central venous access allow for adequate supply of fluids and electrolytes, in addition to macronutrients, vitamins and trace elements.^247^ In order to meet nutritional requirements, malnourished patients need progressive PNT prescription to prevent the incidence of refeeding syndrome. Parenteral nutrition therapy must be gradually introduced after assessment of the following criteria: availability of central venous access, nutritional status or performance status, presence of organ dysfunctions, and metabolic disorders.

The transition from PNT to oral diet can be done as soon as gastrointestinal symptoms improve. Foods must be introduced based on patient tolerability, starting with liquid and soft foods, and then progressing to the usual diet. Parenteral nutrition therapy discontinuation must also be gradual, by cutting the calorie-protein supply in half, according to food acceptance. Parenteral nutrition therapy can be discontinued when oral intake exceeds 50% of nutritional recommendations.^46^

Although PNT is a valuable resource in the management of patients undergoing HSCT, some circumstances may limit its use, such as an infected venous access, severe lipid and glucose metabolism disorders, fluid overload, and progressive liver dysfunction. Transplant units that have developed protocols that prioritize EN have shown favorable results in the group of patients studied.^246^

### Vascular access

The advent of deep venous catheters, with their semi-implantable and fully-implantable variants, allowed for more safety and better local care for prolonged PNT in HSCT. In general terms, patients scheduled to undergo autologous transplantation receive a short-term, multilumen, deep venous catheter, with one of the lumens dedicated to PNT, when needed. Patients scheduled for allogeneic transplant, on the other hand, receive long-term, semi-implantable deep venous catheter, to facilitate care in the post-transplant period.

Determination of the vascular access used for administration of PNT must consider two fundamental factors: the duration of PNT and the osmolarity of the solution. Table 14 presents the criteria for determination of vascular access.

**Table 14:** Indications of peripheral and central parenteral nutrition

Peripheral parenteral nutrition	Central parenteral nutrition
Low concentrations of nutrients Duration of nutritional therapy less than 2 weeks Supplemental to oral and/or enteral nutrition	High nutrient concentrations Safe central venous site Prolonged nutritional therapy
**Observation**	**Observation**
Tip of the venous catheter out of the vena cava Maximum limit 900mOsm/L of solution Less complexity and lower costs (to place the catheter) New options of venous catheters for peripheral access	Allows for administration of hyperosmolar solutions Dedicate one line of the central venous catheter for infusion of parenteral nutrition therapy To calculate osmolarity of the parenteral nutrition therapy: {[gAas/L×10] + [gGlic/L×5] + [mEq_(Na+K)/L×2] + [mEq_Ca/L ×1.4] + [mEq_Mg/L×1]} = Total Osm

Source: Pittiruti M, Hamilton H, Biffi R, MacFie J, Pertkiewicz M; ESPEN. ESPEN Guidelines on Parenteral Nutrition: central venous catheters (access, care, diagnosis and therapy of complications). Clin Nutr. 2009;28(4):365-77.^(248)^

### Formulation and prescription

The basis of protein supply in PNT are essential and non-essential amino acid formulations. The standard 10% formulation for adults contains approximately 4kcal/g. Protein supply to critically-ill patients is crucial for availability and optimization of the metabolic demand for protein synthesis and tissue repair, and the muscle compartment is the main endogenous source of protein turnover. Glucose solutions also provide 4 kcal/g and probably represent, on average, 70% of non-protein calorie sources. Lipid emulsions are the most variable component of the formulation, due to different sources, and provides 10% to 20% of medium-chain, long-chain, monounsaturated-rich, or omega-3 triglycerides. On average, the lipid source at 20% provides 2kcal/mL and accounts for 30% of calorie supply of the solution. Macronutrient recommendations are presented in table 15.

**Table 15:** Recommended prescription of macronutrients for adult patients on parenteral nutrition therapy (ideal weight)

Macronutrients	Critically-ill patients	Stable patients
Proteins	1.2-2.0g/kg/day	1.2-1.5g/kg/day
Glucose	<3mg/kg/minute	<5mg/kg/minute
Lipids	0.5-1.0g/kg/day	0.7-1.3g/kg/day
Total calories	25-30Kcal/kg/day	30-35Kcal/kg/day
Liquids	Volume needed to provide macronutrients	30-35mL/kg/day

Source: Peterson S, Chen Y. Systemic approach to parenteral nutrition in the ICU. Curr Drug Saf. 2010;5(1):33-40.^(249)^

Patients with moderate mucositis and diarrhea must use the protein target defined for clinically stable patients, *i.e*. 1.2 to 1.5g/kg/day. In the presence of severe mucositis, or changes related with severe infections, a higher protein target must be aimed at. To make the most of the protein supply, the non-protein calorie content for each gram of nitrogen should be 100:1 to 130:1. Although the physiological properties of glutamine have incited great interest in its use for EN and PNT, there are no robust studies recommending its inclusion in the PNT solution.^232^ In fact, there could be a negative effect on the risk of late disease relapse (lymphomas).^250^

Glucose control is of utmost importance during PNT, since it minimizes the risk of complications and favors restoration of the nutritional status.^216^ Hyperglycemia is related to an increased risk of infections, metabolic disorders, organ dysfunctions and mortality,^251^ and is a common effect of the use of calcineurin inhibitors and corticosteroids, requiring glucose monitoring and insulin use. Thus it is necessary to design a glucose control protocol that fits the type of NT used and allows for prevention of hypoglycemia, glycemic variability, and significant hyperglycemia.^252,253^ There are no specific recommendations for the ideal glucose range, and the consensus for inpatients is between 110 and 180mg/dL.

In patients undergoing allogeneic HSCT for hematological tumors, lower rates of fatal GVHD were observed in those receiving parenteral formula with a high content of long-chain fatty acids (80% of non-protein calories *versus* carbohydrates); however this is a ketogenic nutrition strategy that should only be used for short periods.^254^ On the other hand, with transplanted patients exposed to the lipid-increasing effects of immunosuppressants, the use of long-chain triglycerides in the PNT composition must be restricted.^255^

Still today, there are few studies on the use of omega-3 lipids during HSCT.^255^ The literature recognizes that its parenteral use attenuates inflammatory activity in a short time (days); however, the ideal dose required, the time of administration, and safety-related issues due to changes in platelet function during critical levels of thrombocytopenia, result in the lack of recommendations for its use in the initial phase of the transplant.

Electrolytes, minerals, vitamins and trace elements must be adjusted to individual needs. Some factors, such as GVHD, antibiotics, metabolic stress, immunosuppressants, diarrhea, and vomiting may alter micronutrient requirements. Therefore, serum electrolytes should be assessed daily for potential adjustments.^216^

As for micronutrients, there are no definitive studies with recommendations targeted at patients undergoing HSCT, and the context in which the main changes in metabolic demands occur, such as oxidative stress, liver or kidney dysfunction, must be kept in mind.^256^ Unfortunately, there are no commercially available products containing trace elements in isolation, which leads to discontinuation of all trace element prescriptions when these organ dysfunctions occur.^260-262^ Biological deficiency of a micronutrient precedes its clinical manifestation.^263^ Cases of Wernicke encephalopathy by acute thiamine deficiency during PNT initiation have been reported after long fasting times, vitamin C depletion due to oxidative stress, and zync deficiency associated with voluminous diarrhea.^256^ The daily recommendations for electrolytes, vitamins and trace elements are presented in tables 16 to 18 [Table t30] [Table t31], respectively.

**Table 16:** Recommended daily amounts of electrolytes prescribed for adult patients on parenteral nutrition therapy (ideal weight)

Electrolytes	Recommendation
Calcium (gluconate)	10-15mEq
Magnesium (sulphate)	8-20mEq
Phosphate (glycerophosphate)	20-40mmol
Sodium (chloride, acetate or phosphate)	1-2mEq/kg
Potassium (chloride, acetate or phosphate)	1-2mEq/kg
Chloride	As needed to maintain acid-base balance
Acetate	As needed to maintain acid-base balance

Source: Adapted from Peterson S, Chen Y. Systemic approach to parenteral nutrition in the ICU. Curr Drug Saf. 2010;5(1):33-40.^(249)^

**Table 17 t30:** Recommended daily amounts of vitamins prescribed for adult patients on parenteral nutrition therapy

Vitamin	Recommendation (FDA, 2000)
A	3,300UI (1mg)
D	200-600UI (5 to 15µg)
E	10UI (10mg)
B1	6mg
B2	3.6mg
B3	40mg
B5	15mg
B6	6mg
B12	5µg
C	200mg
Biotin	60µg
Folic acid	640µg
K	150µg

Source: Adapted from Peterson S, Chen Y. Systemic approach to parenteral nutrition in the ICU. Curr Drug Saf. 2010;5(1):33-40.^(249)^

FDA: Food and Drug Administration.

**Table 18 t31:** Recommended daily amounts of trace elements prescribed for adult patients on parenteral nutrition therapy

Trace element	Recommendation
Zync	3-6.5mg
Copper	0.3-0.5mg
Chrome	10-15µg
Manganese	60-100µg
Selenium	60-100µg
Iodine	130µg (prolonged PN)
Iron	1.1mg (prolonged PN)

Source: Singer P, Berger MM, Van den Berghe G, Biolo G, Calder P, Forbes A, et al. ESPEN Guidelines on Parenteral Nutrition: intensive care. Clin Nutr. 2009;28(4):387-400.^(263)^

PN: parenteral nutrition.

In respect to adjustment of macro and micronutrients when prescribing PNT, we must consider the existence of two systems: the individualized PNT and industrialized PNT, previously described as ready to use (RTU). Although the individualized version gets closer to meeting the requirements of each patient, most centers use the industrialized version, without any harm to patients.^264^

The addition of these PNT components upon installation favors their bioavailability, but compromises safety due to the generation of physical and chemical imbalances in the solution, or increased risk of infection during handling.^264^ As regards commercially available products, their composition, in general, also strays away from the recommendations in table 15.

Compounded PNT solutions follow pharmacological principles and are finished by the pharmacist, which favors a more adequate supply of nutrients to transplanted patients, however they must be produced in compliance with safety standards (therapeutically and pharmaceutically appropriate, free from pyrogenic substances, sterile, at the right dose and composition, identified, stored and administered within the defined shelf-life). Nonetheless, when the calculation of nutritional needs fits into a RTU formulation, this option becomes cost-effective, and facilitates the process involved.^264^

The use of IC is greatly relevant for PNT prescription, promoting better understanding of the metabolic demand and individualization of calorie-protein requirements. The combination of specific recommendations for critically-ill patients and large-scale production of new RTU formulations seeks to promote their use even in children.^265^

### Complications

Parenteral nutrition therapy is related with adverse metabolic effects due to inadequate estimates of nutritional requirements, extent of the catabolic state, and problems with central or peripheral venous access; in addition PNT might reduce response to chemotherapy There is a relative consensus that it is preferable to adopt conservative calorie-protein supply, of about 25kcal/kg/day and up to 1.5g/kg/day, in order not to induce metabolic or fluid overload, which could impair liver or kidney function. The onset of sinusoidal obstruction syndrome is the most serious non-infectious complication of the initial stage of HSCT, resulting from conditioning toxicity in both autologous and allogeneic HSCT, and it usually manifests in the first three weeks of treatment. The clinical context of weight gain, hyperbilirubinemia, followed by oliguria and hepatic encephalopathy, in its more severe form, will require discontinuarion of PNT. Other metabolic complications are related with hyperglycemia, prolonged use of diuretics, and slowed-down appetite recovery. There is a recommendation for addition of regular insulin to the PNT solution, considering a safe ratio of grams of glucose per unit of insulin (usually starting at 20:1 to 10:1). In peripherally administered PNT solutions, the addition of hydrocortisone and heparin prevents inflammatory complications and early withdrawal of the catheter. In case of increased liver enzymes outside the context of sinusoidal obstruction syndrome, it is recommended to change the PNT infusion frequency to 12- to 20-hour cycles, reduce the supply of non-protein calories (10% to 15% less than the previous), check for any micronutrient defiencies (coline, carnitine and taurine), stimulate oral intake, and adjust the ursodeoxycholate dose to 20 to 30mg/kg/day. Consider the use of metronidazole to reduce portal endotoxemia. In cholestatic conditions, the use of copper and manganese must be limited due to neurotoxicity.

The nursing team must supervise PNT administration, including management of the PNT infusion time, adjusting according to other drug infusions scheduled. It is recommendable to administer multivitamin supplements in the first half of the day, and trace elemnts in the second half of the day. Electrolytic *deficits* must be corrected with concentrated solutions, to minimize fluid overload (in case of central venous access), with observation of the maximum safe limits for infusion of each electrolyte.

Venous access is the most common source of PNT-related complications, particularly those of infectious origin. Other complications include local thrombosis, venous thromboembolism, displaced and fractured catheters, and leaks from mechanical damage.^12^

Parenteral nutrition therapy is a procedure inherent to HSCT, and must observe the same quality and safety criteria applicable to the transplant. Changes associated with PNT administration and enteral nutrient deprivation are complex and only partially known, with multiple potential fields of study, which could help increase the safety of this therapy. In the future, further studies are needed to address the impact of sequential or combined use of EN and PNT on clinical outcomes, and complication rates.^47^ Specifically in the case of glutamine and omega-3 fatty acids, there is still a need for studies investigating their effects on mucositis, diarrhea, infections, GVHD, and relapse rates.

However, it is important that these studies separate patients by nutritional status, tumor type (solid *versus* hematological) and transplant type (autologous *versus* allogeneic), in order to reduce interpretation biases resulting from the combined analysis of these characteristics in the same study.

### Enteral nutrition

Enteral nutrition consists of a set of therapeutic procedures for maintenance or recovery of nutritional status, through enteral nutrition, administered either orally or through a tube accessing the digestive system. American Society Parenteral and Enteral Nutrition and ESPEN have published guidelines on nutritional support for HSCT patients, recommending artificial NT to malnourished patients, or those with impaired food intake or intestinal absorption for a prolonged period of time.^40,47,266^

In the context of HSCT, the best NT must be established to prevent weight loss and deterioration of the nutritional status, considering the increased nutritional requirements, gastrointestinal toxicities interfering with food tolerance, and the psychosocial conflicts involved in this procedure.

These factors favor the deterioration of nutritional status, quality of life, physical activity, and lean body mass, for up to 100 days post-HSCT.^63,93,267,268^ Also, patients presenting with weight loss for up to 3 months after allogeneic HSCT are at high risk of mortality not associated to relapse, and worse survival.^269^ It is clear that nutritional intervention should be part of HSCT therapy, since it contributes to treatment success.

In respect to the NT of choice, PNT is still the first choice in many HSCT centers, due to the easiness of administration through a central venous access, and due to it being considered by healthcare teams, family members and patients as less traumatic and uncomfortable.^24,270^ However, EN is more physiologic and maintains intestinal tropism, which is essential to avoid bacterial translocation and subsequent systemic infection.^242^

Challenging the usual choice for PNT, studies have shown that it can promote intestinal atrophy and abnormal gastrointestinal immunity, due to decreased cellularity of the GALT (gut-associated lymphatic system) and vascular perfusion, significantly reducing the number of lymphocytes in the lamina propria. There are also important changes in intestinal IgA release and antimicrobial production by Paneth cells (ileal lymph node conglomerates), compromising the epithelial barrier and chemical cleaving, altering the microbiome (dysbiosis) and increasing susceptibility to infections.^271,272^

The loss of integrity of the intestinal mucosa decreases nutrient absorption, facilitates colonization by pathogenic bacteria, and promotes infections by translocation. In addition, exsudative enteropathy, proteolysis and nitrogen loss, as consequences of this process, contribute to some complications, such as sepsis and GVHD. These can be prevented with the use of EN, which makes it a good choice.^75,271^

A study by Seguy et al.,^273^ demonstrated better 100-day survival in patients on EN, compared to patients on PNT. In another study by the same authors, EN resulted in lower GVHD incidences since it limited intestinal atrophy.^43^In another comparison between the two treatments, patients on PNT had higher risk of infection.^242^

In line with these findings, several other clinical trials have considered EN just as effective as PNT, however with lower complication rates, in addition to better survival, lower incidence of acute GVHD, and faster neutrophil recovery. Parenteral nutrition therapy was only recommended in cases of severe mucositis or gastrointestinal insufficiency.^47,232^

Thus, EN must be stimulated during HSCT, challenging preconceptions of patients, family members and the professionals involved. For this purpose, we must encourage the creation of nutritional care protocols^246^that value EN as a feasible and viable option, even if it requires skill development for management of gastrointestinal toxicities arising from this therapeutic modality.

### Indication for enteral nutrition therapy

Enteral nutrition is indicated for patients with oral acceptance <60% of nutritional requirements, before HSCT, or in the late post-transplant period, as well as malnourished patients with dietary acceptance (diet + oral NT) <60% of nutritional requirements prior to HSCT or in the late post-transplant period (without the presence of mucositis and thrombocytopenia).^246^ Consider the risks and benefits of EN if there is an indication for refusal of oral NT pre-HSCT.^47^

Patients with a history of inadequate/erratic eating and poor adherence to nutritional guidance, based on observations of the pre-HSCT assessment, must be individually assessed for indication of EN, in an attempt to identify, together with professionals from each specific area, if there are any psychosocial issues interfering with treatment.

Indication may be considered in patients undergoing myeloablative HSCT, due to the potentially higher toxicity, and in elderly patients on high-dose melphalan for autologous HSCT, because of the higher risk of developing severe mucositis, although there is no evidence for formal EN indication in this population.^242,274^

The enteral feeding tube must be inserted early on, due to gastrointestinal toxicities.^275^ It is suggested the insertion of the catheter of enteral nutrition until D+3, when gastrointestinal toxicities are not yet pronounced.

The healthcare team, the patient and family members must be approached about the possibility of EN indication at the pre-HSCT assessment, when questions and myths about NT should be clarified, and benefits explained.^30,242^ The healthcare team must be encouraged to use EN as something beneficial to the treatment and the patient, and understand that NT in HSCT must be focused on preventing^72,275^ deterioration of the nutritional status and nutrition-related risks to patients.

In patients able to receive oral nutrition, once EN is initiated, oral feeding may be continued and should be stimulated for faster and adequate weaning from EN. There is an indication for EN in patients weaning from PNT with food acceptance ≥60% of requirements, for 3 consecutive days.^246^

### Position of the nasoenteral catheter/tube

Gastric positioning is preferred due to better tolerability to enteral formulations, withstanding osmotic overload, and greater storage capacity when compared to the bowel. If the tube is displaced due to vomiting, it must be reinserted in an enteric position. If the nasoenteral tube has to be reinserted more than twice, PNT should be evaluated for its risks and benefits.

The use of gastrostomies must be reserved for patients with malnutrition and impaired oral intake due to the underlying disease,^47^ and the procedure must be done before the transplant, as part of disease management.

### Enteral diet formulations

The use of industrialized EN is suggested due to the lower risk of contamination and the better supply of macro- and micronutrients for HSCT patients. The enteral formulation indicated is one that meets the nutritional requirements of patients with good tolerability. The use of polymeric, high-calorie, high-protein EN is advocated.^30,40,246^ Continuous infusion is recommended, with the use of a pump for better control of the diet. It is fundamental that the team establish the criteria for fulfillment of patients’ nutritional needs in the shortest time possible.^276^

To complement oral nutrition, Bay et al.,^30^ recommended nocturnal EN infusion, with a 10-hour infusion duration, starting with 20mL per hour and progressing to 10mL to 20mL per night, aiming to administer 1,000 to 1,500kcal per day.^30^

Enteral nutrition monitoring must occur to ensure patient tolerability of the therapy initiated, metabolic balance, and toxicity detection.^30,277^ This monitoring, irrespective of the NT adopted (oral nutrition, EN or PNT), must be associated with surveillance of nutritional status, fluid balance, and kidney and liver function throughout the entire HSCT procedure.^30^

### Contraindications of enteral nutrition therapy

The main contraindications for maintaining or indicating Enteral nutrition are mucositis, digestive bleeding, neutropenic colitis, incoercible vomiting and diarrhea (grades III and IV) refractory to drug therapy, gastrointestinal tract obstruction, ileus, severe intestinal GVHD (grades III and IV) refractory to drug therapy, and sinus disease. The risk of mucositis, if not already installed, is not a contraindication for EN.^40,47,94,246,275^

Table 19 presents the main EN-related complications.

**Table 19:** Main complications related with enteral nutrition therapy

Complications	Description	Preventive measure
Mechanical	Displacement or accidental removal of the nasoenteral tube Obstruction of the nasoenteral tube	Ensure proper fixation of the nasoenteral tube, marking the exit point with indelible ink to monitor the positioning Always flush the nasoenteral tube with filtered water after administration of the enteral diet, at every pause in the diet, before and after administration of each medication Verify the position of the nasoenteral tube whenever there is cough, vomiting or an agitated patient If vomiting is present, pause diet and feeding for 15 minutes, and continue to monitor for new episodes of vomiting Use a 20mL syringe with warm water to unclog the nasoenteral tube
Gastrointestinal	Nausea and vomiting Diarrhea Bloating Constipation Abdominal discomfort Reflux esophagitis	Observe good practices of preparation and conservation of enteral diets (if in doubt, ask the dietitian about prior storage, installation and expiry date of the product). Control dripping or infusion volume using continuous infusion pump (CIP) Ensure proper hygiene and temperature on diet administration Keep patient in the semirecumbent position (30 to 45°) during and for 30 minutes after feeding
Lung	Bacterial colonization Aspiration Sepsis Pneumonia Pneumothorax	Monitor laboratory tests and X-rays to check positioning of the nasoenteral tube Keep patient in the semirecumbent position before and after administration of enteral diets Prefer the jejunal position of the nasoenteral tube in patients at risk for bronchoaspiration
Ear nose throat	Injury Necrosis or nasal abscess Sinusitis Hoarseness Otitis	Use a flexible nasoenteral tube On prolonged EN, replace tube every 3 months Ensure proper fixation and cleaning of the tube and patient nostril When changing the nasoenteral tube, use the other nostril

Source: August DA, Huhmann MB; American Society for Parenteral and Enteral Nutrition (A.S.P.E.N.) Board of Directors. A.S.P.E.N. clinical guidelines: nutrition support therapy during adult anticancer treatment and in hematopoietic cell transplantation. JPEN J Parenter Enteral Nutr. 2009;33(5):472-500.^(40)^ Stroud M, Duncan H, Nightingale J; British Society of Gastroenterology. Guidelines for enteral feeding in adult hospital patients. Gut. 2003;52(90007 Suppl 7):vii1-12.^(277)^ Waitzberg D, Dias MC, Isosaki M. Manual de boas práticas em terapia nutricional enteral e parenteral do HCFMUSP (Hospital das Clínicas da Faculdade de Medicida da Universidade de São Paulo). 2nd ed. São Paulo: Atheneu; 2015. 431 pp.^(278)^ Bankhead R, Boullata J, Brantley S, Corkins M, Guenter P, Krenitsky J, et al. Special Report Enteral Nutrition Practice Recommendations. 2009;33(2):122-67.^(279)^ Harris AE, Styczynski J, Bodge M, Mohty M, Savani BN, Ljungman P. Pretransplant vaccinations in allogeneic stem cell transplantation donors and recipients: an often-missed opportunity for immunoprotection? Bone Marrow Transplant. 2015;50(7):899-903. Review.^(280)^

### Gastrointestinal toxicities

During HSCT, multiple gastrointestinal toxicities are observed as a result of the action of chemotherapeutic agents during conditioning, total body irradiation, and antimicrobials. There are many conditioning regimens, leading to variable compromise of the gastrointestinal tract, which can be graded using the Common Terminology Criteria for Adverse Events (CTAE), the monitoring of which is extremely important during EN use.^281^

In non-Hodgkin lymphoma, a comparison of toxicities from three conditioning regimens showed greater toxicities for the BEAM regimen, with mucosal impairment (greater than grade II), diarrhea and fever; with BEAC (carmustine, etoposide, cytarabine and cyclophosphamide), in turn, patients had more anorexia, vomiting and bleeding; and in CBV (cyclophosphamide, carmustine and etoposide), the incidence of toxicities was lower.^282^ In another investigation, neutropenic colitis was more frequent in patients with non-Hodgkin lymphoma treated with BEAM.^283^

Nausea, vomiting, loss of appetite, diarrhea and mucositis do not contraindicate the use of EN, but these symptoms hinder appropriate nutrition of patients undergoing HSCT, and must be evaluated and closely monitored for better adjustment of NT.

The use of protocols for management of nausea and vomiting must be encouraged in these services. Vomiting causing tube displacement does not contraindicate EN, however it requires post-pyloric placement of the enteral nutrition tube.

Cases of diarrhea must be evaluated for the possibility of infections such as *C. difficile*, and frequency and volume must be checked daily. Management must be conducted by the medical and nutrition teams. In case of voluminous diarrhea, with no improvement for three days, after ruling out potential infectious causes and gastrointestinal GVHD, diet volume and formulation must be reassessed. It is essential that the patient be informed that the diarrhea is not caused by EN.

However, a study investigating gastrointestinal symptoms during autologous and allogeneic HSCT with different conditioning regimens found that most patients consuming <60% of calorie requirements until engraftment had less diarrhea. This fact may be due to the intake of nutrients that contribute for recovery of the intestinal epithelium, with a positive impact on oral intake.^284^

One possible therapy for diarrhea is the use of probiotics, which contribute for prevention and treatment of antibiotic-associated diarrhea.^285,286^ However, their safety and efficacy have not yet been established and, therefore, they are not yet indicated via EN in patients undergoing HSCT. Only one strain has been proven safe in children and adolescents during HSCT-associated neutropenia, but its use in adults and the elderly are not yet established, as well as the appropriate doses.^228^

As for the choice of enteral formulations during episodes of diarrhea, the use of elemental or semielemental formulations is controversial and must be individually assessed, considering these formulations have higher osmolarity, and diarrhea taking place during the course of HSCT is usually osmotic.

Addressing gastrointestinal toxicities and the use of EN in patients submitted to myeloablative HSCT, a recent study with 28 patients in each group showed that 92% of those on EN and 95% of those in PNT has nausea and vomiting. Diarrhea was observed in 44% and 54% of patients on EN and PNT, respectively. Enteral nutrition was discontinued in only 28% of patients due to change in body image, incoercible vomiting, and displacement or obstruction of the enteral nutrition tube, when mucositis was already present and prevented the feeding tube from being reinserted.^242^ This observation demonstrated the feasibility of routine use of EN in patients undergoing HSCT.

The American, French and European societies agreed that the first-choice NT, when the gastrointestinal tract is functioning, should be enteral rather than parenteral, due to the potential infectious complications or metabolic implications. Management of gastrointestinal toxicities must be adopted as a daily practice by healthcare teams.^30,40,47^ Gastrointestinal toxicities, such as mucositis, are not a limitation for the use of EN, provided the tube has been previously inserted.^30^

### Infection related to the use of enteral feeding tube and enteral nutrition therapy

Comparatively, EN has lower correlation with infection than PNT.^242^ The risk of EN-related infection results from insertion of the tube for enteral feeding, inadequate handling of enteral diets as well as of the enteral feeding tube, incorrect storage of the diet to be offered, and incorrect reuse of tubes and feeding containers for food.

### Insertion of the enteral nutrition tube

The three must frequent accesses are nasogastric, nasojejunal, and percutaneous endoscopic gastrostomy. The risk of infection is related with insertion of the tube (nasogastric and nasojejunal), contact with the hand of the healthcare professional guiding the catheter and, in gastrostomy, placement of the tube and infection of the insertion site.

### Compounding of enteral diet

The healthcare unit must follow the Good Manufacturing Practices for Enteral Nutrition, as per ANVISA’s Collegiate Board Resolution 63, from 2000.^266^

The dietitian is responsible for supervising preparation of enteral nutrition. Enteral nutrition preparation involves reviewing the diet prescription, compounding, quality control, conservation and transport of enteral nutrition, under responsibility and direct supervision of the dietitian. Enteral nutrition compounding must be carried out using aseptic technique, and following written and validated procedures.

Enteral nutrition must be labeled with clear identification of the patient’s name, composition, and other legal and specific information, to ensure safe use and traceability. Dietitians are responsible for maintaining the quality of EN until its delivery to the professional responsible for administration, and they must guide and train the employees in charge of its transport.

For closed-system EN, the manufacturer’s recommendations regarding conservation and transport must be followed. The use of a closed system is generally preferred, since the product is sterilized, there is no local handling, and the tube is accessed fewer times, which reduces the chance of infection.^287^

Enteral feeding produced the same organisms that cause food poisoning, with similar symptoms, such as abdominal discomfort and/or bloating, nausea, vomiting and diarrhea, which, in immunocompromised patients, can lead to bacteremia, sepsis and even death.^277,288,289^

### Storage and administration

After EN products are received, the nurse is responsible for their conservation and administration.^266^

To reduce biological risks, the nursing team must:

- Visually inspect EN before its administration. If any abnormalities are detected in enteral nutrition, it must not be administered, the dietitian in charge must be contacted, and the EN product must be returned.
- Confirm the position and patency of the tube before initiating enteral nutrition administration.
- Properly wash hands before proceeding with arrangements for administration of enteral nutrition.
- Administer enteral nutrition with strict observation of the duration established.
- If the administration has to be paused, closed-system EN must comply with manufacturer’s instructions regarding its stability time once opened. Compounded EN (open system) must be administered within 4 hours once opened.^287^

Enteral nutrition is inviolable until the end of administration, and cannot be transferred to any other container. Administration must ensure the safety of the patient and maximize effectiveness in respect to costs, using standardized materials and techniques.

There are multiple ways EN can be contaminated in a hospital, home or clinic, such as:^290^

- Poor hand washing: lack of hand washing can lead to contamination of enteral nutrition, tube, system, and EN container.^290^
- Inadequate cleaning of reusable devices during and after use: the system and the infusion pump must be kept clean while in use, and completely cleaned after feeding is completed.^287^
- Inadequate installation of enteral feeding: handling of the catheter and container without the appropriate technique.^287^
- Poor tube maintenance: the tube must be regularly flushed with filtered water to reduce the build-up of residues and drug inside, which could cause obstruction and become a source of contamination. Tubes for enteral feeding must be flushed with 20mL to 30mL of water, every 4 to 6 hours, during continuous feeding, during feeding pauses, and before and after drug infusions.
- Indequate use of the system: all tubes used in enteral feeding are designed for this specific purpose. Ideally, an enteral feeding system should not connect to any other access. It is possible to administer drugs through the enteral feeding tube; however, the pharmacy staff at the hospital must educate on the correct handling and administration of these substances.

Education and training on infection prevention and control are essential.^288,291^ The expertise of the nurse is vital to prevent bacterial contamination in enteral nutrition.^290^ Training of the nursing staff on proper handling procedures and enteral nutrition protocols can reduce the level and incidence of bacterial contamination in EN, as well as improve EN offer and impact clinical outcomes of HSCT patients.^287,288,291,292^

### Reuse of materials

Most hospitals and clinics recommend single use of devices, which must be discarded afterwards. Nurses are responsible to ensure the proper disposal of all devices used. A tamper-proof safety mechanism must be considered. These errors may not sound as serious as drug administration errors, for example, however the effects of such misconducts can be very serious to patients.^289,290^ The reuse of devices may affect their integrity and increase the likelihood of bacterial contamination.^290^

Another product that must be used only once is the syringe used to flush catheters. If syringes must be reused, they must be washed right after use with soap and water. The plunger should be removed to allow for thorough cleaning. Other cleaning methods approved include dishwasher, soaking in boiling water (3 minutes), cold sterilization solution, and microwave steam sterilizers. Once dry, store in a clean, dry container. Wait to reassemble the syringe right before use. It must be strictly used in only one patient. Syringes are validated for 30 uses. For most patients, the syringe must be changed once a week (equivalent to a maximum of four doses per day). However, if the syringe is used more than four times a day, it must be changed more frequently.

### Weaning

Enteral nutrition discontinuation depends on acceptance of oral nutrition, and oral intake must reach at least 60% of nutritional requirements for three consecutive days.^246^ After resolution of all gastrointestinal toxicities, such as mucositis, and restablishment of oral feeding, oral intake must be stimulated and oral nutrition therapy must be initiated for adequate supply and consumption of nutrients. When the oral intake of foods and supplements combined is equal to or greater than 60% of individual nutritional requirements, for three consecutive days, EN must be suspended.^40,47,246^

### The importance of using nutritional therapy indicators

Nutritional therapy indicators enable quality management of the nutritional care provided to patients. These indicators, after analysis of their results, allow for critical assessment of the actions performed, and implementation of corrective measures to improve results and the quality of patient care.^293^

When these indicators are included in the NT quality assurance program, there are some proven benefits, such as greater efficiency in daily routines, cost reduction, health care practice supported by scientific literature, greater capacity for process analysis, and better clinical and quality of life outcomes for patients.^293,294^

The selection of indicators is based on the needs and possibilities of each service.^278^ Three indicators presented in tables 20 to 22 [Table t34] [Table t35], as technical charts, are related with the content of the EN topic.

**Table 20:** Frequency of compliance of enteral nutrition therapy indication

**Objective**	Verify if the EN is indicated as per the guidelines prescribed in protocols for patients on EN
**Description**	Measure the frequency of EN indications according to previously established guidelines
**Rationale**	Assess the frequency of EN indication in compliance with previously established guidelines, and take measures to ensure compliance with the guidelines for all patients requiring this therapy
**Formulation**	Number of patients on EN indicated as per guidelines × 100 Total number of patients on EN
**Frequency**	Monthly
**Target**	<10%
**Person in charge of this information**	Dietitian and Multidisciplinary Nutritional Therapy Team

Source: Isosaki M, Gandolfo A, Jorge A, Evazian D, Castanheira F, Bittar O. Indicadores de Nutrição Hospitalar. São Paulo: Atheneu; 2015.^(294)^

**Table 21 t34:** Frequency of patients on enteral nutrition therapy reaching 100% of nutritional requirements within 3 days

**Objective**	Monitor the time it takes for patients to meet adequate calorie and/or protein requirements
**Description**	Assess the number of patients reaching calorie and/or protein targets within 3 days from initiation of EN
**Rationale**	Monitor the time until the calorie and protein targets of patients are met
**Formulation**	Number of patients on EN meeting 100% of adequate calorie and/or protein requirements* × 100 Total number of patients on EN
**Frequency**	Monthly
**Target**	≥80% or according to the needs of the unit
**Person in charge of this information**	Dietitian and Multidisciplinary Nutritional Therapy Team

Source: Waitzberg DL, editor. Indicadores de qualidade nutricional: aplicação e resultados. São Paulo: Atheneu; 2010;^(293)^ Isosaki M, Gandolfo A, Jorge A, Evazian D, Castanheira F, Bittar O. Indicadores de Nutrição Hospitalar. São Paulo: Atheneu; 2015.^(294)^

* Within 3 days.

**Table 22 t35:** Frequency of patients on enteral nutrition therapy who recovered oral intake

**Objective**	Measure the frequency of recovery of oral intake in patients on EN
**Description**	Frequency of recovery of exclusively oral intake in patients on EN, ingesting >60% of estimated calorie requirements
**Rationale**	Learn the frequency of patients recovering oral intake after nutritional therapy
**Formulation**	Number of patients recovering oral intake* × 100 Total number of patients on EN
**Frequency**	Monthly
**Target**	>80%
**Person in charge of this information**	Dietitian

Source: Isosaki M, Gandolfo A, Jorge A, Evazian D, Castanheira F, Bittar O. Indicadores de Nutrição Hospitalar. São Paulo: Atheneu; 2015.^(294)^

* ≥60% of estimated calorie requirements.

**Figure 1:**
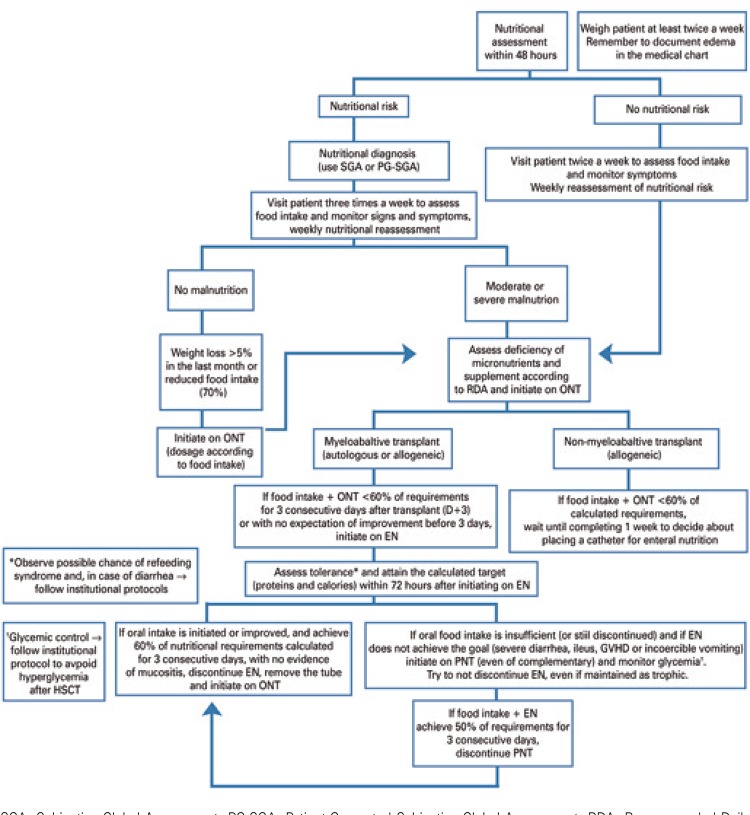
Flow chart for decision-making regarding admission for hematopoietic stem cell transplantation SGA: Subjective Global Assessment; PG-SGA: Patient-Generated Subjective Global Assessment; RDA: Recommended Daily Allowance; ONT: oral nutrition therapy; EN: enteral nutrition; GVHD: graft-versus-host disease; PNT: parenteral nutrition therapy.

**Figure 2:**
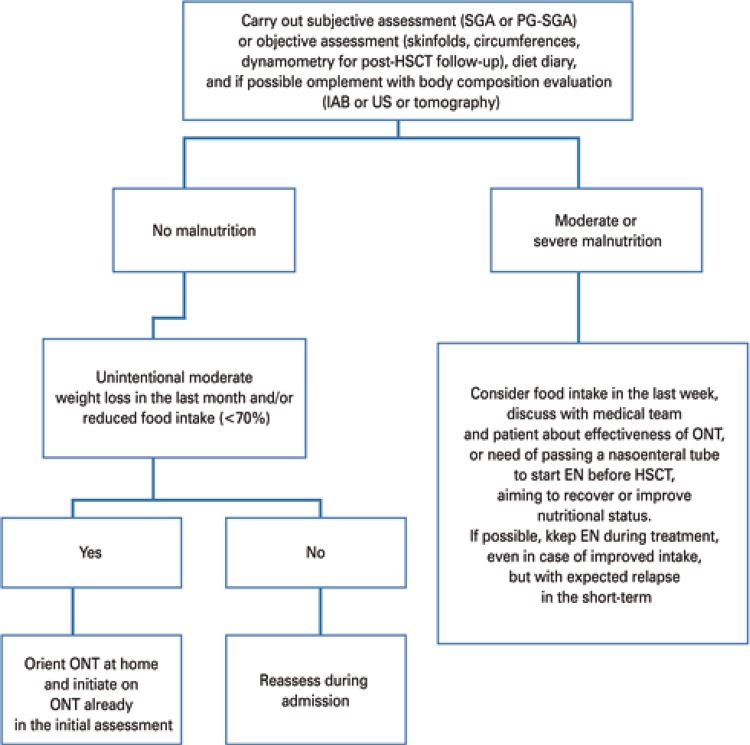
Flow chart for decision-making in nutritional assessment before hematopoietic stem cell transplant (HSCT) SGA: Subjective Global Assessment; PG-SGA: Patient-Generated Subjective Global Assessment; IAB: intraaortic balloon; US: ultrasound; ONT: oral nutrition therapy; EN: enteral nutrition.

Figures 1 and 2 present the flow charts for decision-making.^39,47,119,242,246,295-297^
