# Supplementary material for: Brazilian Nutritional Consensus in Hematopoietic Stem Cell Transplantation: Adults
Source: Einstein (Sao Paulo). 2020 Jan 31;18:AE4530. doi: 10.31744/einstein_journal/2020AE4530 (PMC6999189; doi:10.31744/einstein_journal/2020AE4530)
Supplement: Translation

# Translation: Consenso Brasileiro de Nutrição em Transplante de Células-Tronco Hematopoiéticas: Adultos

**Authors:** Juliana Bernardo Barban, Belinda Pinto Simões, Bruna Del Guerra de Carvalho Moraes, Cássia Rehem da Anunciação, Cristiane Santos da Rocha, Daniela Cristina Querino Pintor, Daiane Cristina Guerra, Deise Andrade Silva, Edith de Castro Martins Brandão, Fábio Kerbauy, Fernanda Ramos de Oliveira Pires, Graciele Lima Morais, Jayr Schmidt, Juliana Maria Faccioli Sicchieri, Karine Sampaio Nunes Barroso, Luciana Verçoza Viana, Mariana Hollanda Martins da Rocha, Maysa Penteado Guimarães, Natalia Leonetti Couto Lazzari, Nelson Hamerschlak, Plinio Patricia Ramos, Plinio Nascimento Gomes, Priscila da Silva Mendonça, Raquel Candido de Oliveira, Renata Corrêa Scomparim, Ricardo Chiattone, Rosa Wanda Diez-Garcia, Thaís de Campos Cardenas, Thais Manfrinato Miola, Thalita Cristina de Mello Costa, Vanderson Rocha, Andrea Z Pereira

**Affiliations:** 01 Brasil Escola Paulista de Medicina, Universidade Federal de São Paulo, São Paulo, SP, Brasil.; 02 Brasil Hospital das Clínicas, Faculdade de Medicina de Ribeirão Preto, Universidade de São Paulo, Ribeirão Preto, SP, Brasil.; 03 Brasil Hospital das Clínicas, Faculdade de Medicina, Universidade de São Paulo, São Paulo, SP, Brasil.; 04 Brasil Hospital Leforte Liberdade, São Paulo, SP, Brasil.; 05 Brasil Hospital Santa Marcelina, São Paulo, SP, Brasil.; 06 Brasil Centro de Transplante de Medula Óssea, Instituto Nacional de Câncer José Alencar Gomes da Silva - INCA, Rio de Janeiro, RJ, Brasil.; 07 Brasil IBCC Oncologia, São Paulo, SP, Brasil.; 08 Brasil Universidade Federal de São Paulo, São Paulo, SP, Brasil.; 09 Brasil A.C.Camargo Cancer Center, São Paulo, SP, Brasil.; 10 Brasil Hospital São Rafael, Salvador, BA, Brasil.; 11 Brasil Hospital Universitário Walter Cantídio, Universidade Federal do Ceará, Fortaleza, CE, Brasil.; 12 Brasil Hospital de Clínicas de Porto Alegre, Porto Alegre, RS, Brasil.; 13 Brasil Hospital Samaritano, São Paulo, SP, Brasil.; 14 Brasil Hospital Israelita Albert Einstein, São Paulo, SP, Brasil.; 15 Brasil Universidade Estadual de Campinas, Campinas, SP, Brasil.

**Keywords:** Transplante de células-tronco hematopoiéticas, Estado nutricional, Terapia nutricional

## Abstract

O estado nutricional do paciente submetido ao transplante de células-tronco hematopoéticas é considerado fator de risco independente, podendo influenciar na qualidade de vida e na tolerância ao tratamento proposto. O comprometimento do estado nutricional durante o transplante de células-tronco hematopoéticas ocorre principalmente devido aos efeitos adversos decorrentes do condicionamento ao qual o paciente é submetido. Desta forma, a adequada avaliação nutricional e o acompanhamento durante o transplante de células-tronco hematopoéticas tornam-se imprescindíveis. Com o objetivo de salientar a importância do estado nutricional e da composição corporal durante o tratamento, bem como as principais características relacionadas à avaliação nutricional do paciente, o Consenso Brasileiro de Nutrição em Transplante de Células-Tronco Hematopoiéticas: Adulto foi elaborado visando uniformizar e atualizar a Terapia Nutricional nesta área. Com a participação de nutricionistas, nutrólogos e hematologistas de 15 centros brasileiros referências em transplante de células-tronco hematopoéticas

## INTRODUÇÃO AOS TIPOS DE TRANSPLANTE DE CÉLULAS-TRONCO HEMATOPOÉTICAS

O transplante de células-tronco hematopoéticas (TCTH) é uma modalidade terapêutica consagrada para o tratamento de grande variedade de doenças hematológicas benignas e malignas.^1^

A cada ano, cerca de 50 mil pessoas são submetidas ao TCTH no mundo.^2^ Estima-se que, no ano de 2018, foram realizados 3.091 TCTH no Brasil, segundo dados do Registro Brasileiro de Transplantes, seguindo as perspectivas mundiais de incremento no número de procedimentos realizados.^3^ Além do aumento progressivo do número de centros transplantadores, esse crescimento se deve ao grande número de doadores cadastrados no Registro Brasileiro de Doadores Voluntários de Medula Óssea (REDOME), que ultrapassam 4 milhões de pessoas, consagrando-se como o terceiro maior registro do mundo, aumentando, dessa forma, a possibilidade de se encontrar um doador.^4^

De acordo com o doador, o TCTH pode ser denominado como autólogo, quando a célula-tronco hematopoética (CTH) enxertada é do próprio paciente; alogênico, quando provinda de outro doador; e singênico, quando o doador é um gêmeo univitelino. Nos transplantes alogênicos, o doador pode ser aparentado (HLA compatível ou incompatível − haploidêntico) ou não aparentado (de preferência HLA compatível), proveniente do registro de doadores ou do banco de sangue de cordão umbilical e placentário (SCUP) − compatível ou com certo grau de incompatibilidade HLA. Na tabela 1, encontram-se descritas as modalidades de TCTH. Na tabela 2, estão as principais doenças com indicação de TCTH.^5^

**Tabela 1:** Modalidades de transplante de células-tronco hematopoéticas

Tipos de transplante	Fonte de células-tronco hematopoéticas	Doador
Autólogo	Medula óssea	Próprio paciente
	Sangue periférico	
	Sangue de cordão umbilical e placentário (situações raras)	
Alogênico	Medula óssea	Aparentado (irmão ou outro parente)
	Sangue periférico	
	Sangue de cordão umbilical e placentário	Não aparentado: qualquer pessoa sem laços familiares com o paciente
Singênico	Medula óssea	Irmão gêmeo idêntico
	Sangue periférico	

Fonte: Adaptado de Passweg JR, Halter J, Bucher C, Gerull S, Heim D, Rovó A, et al. Hematopoietic stem cell transplantation: a review and recommendations for follow-up care for the general practitioner. Swiss Med Wkly. 2012;142:w13696. Review.^(5)^

**Tabela 2:** Principais indicações de transplante de células-tronco hematopoéticas

Autólogo
Desordens malignas
Mieloma múltiplo
Linfoma não Hodgkin
Linfoma Hodgkin
Leucemia mieloide aguda
Neuroblastoma
Sarcoma de Ewing
Tumores de células germinativas
Outras neoplasias raras na infância
Desordens não malignas
Doenças autoimunes
**Alogênico**
Desordens malignas
Leucemia mieloide aguda
Leucemia linfoblástica aguda
Leucemia mieloide crônica (refratária a inibidores de tirosino-quinases)
Síndromes mielodisplásica (alto risco)
Neoplasia mieloproliferativa (alto risco)
Linfoma não Hodgkin
Linfoma Hodgkin
Mieloma múltiplo
Leucemia linfocítica crônica
Desordens não malignas
Adquiridas
Anemia aplástica severa
Hemoglobinúria paroxística noturna (não elegível ao tratamento de inibição do complemento)
Congênitas
Anemia de Fanconi e outras síndromes de falência medular
Talassemia
Doença falciforme
Síndrome de imunodeficiência congênita
Erros inatos do metabolismo

Fonte: Traduzido de Passweg JR, Halter J, Bucher C, Gerull S, Heim D, Rovó A, et al. Hematopoietic stem cell transplantation: a review and recommendations for follow-up care for the general practitioner. Swiss Med Wkly. 2012;142:w13696. Review.^(5)^

## TRANSPLANTE DE CÉLULAS-TRONCO HEMATOPOÉTICAS AUTÓLOGO

O TCTH autólogo possui como princípio básico a utilização de altas doses de drogas quimioterápicas, seguida de resgate com CTH, pois, caso contrário, não seriam tolerados pelo paciente, devido à mielotoxicidade intensa com aplasia muito prolongada. Possui como objetivo suplantar a resistência das células tumorais a doses convencionais de quimioterapia, melhorando a resposta ao tratamento.^4^ Dentre as indicações mais frequentes para o TCTH autólogo (Tabela 2), estão pacientes com mieloma, indicado como pilar do tratamento atual para pacientes sem comorbidades importantes; e pacientes com linfoma em primeira remissão (como o linfoma do manto) ou para aqueles como linfomas não Hodgkin ou doença de Hodgkin que recidivam após quimioterapia.

Nesta modalidade de tratamento, as CTH (CD34^+^) são do próprio paciente, coletadas por aférese e criopreservadas para serem devolvidas após administração de doses altas de quimioterapia/radioterapia. As células são coletadas após 5 a 6 dias de estímulo com fator estimulador de colônias granulocítica (GCSF), na dose de 10ug/kg/dia por veia periférica.^5^

As complicações desta terapia relacionam-se à toxicidade do regime de condicionamento, como mucosite, alterações hepáticas, renais, cardíacas e de outros órgãos; além de complicações infecciosas durante o período de aplasia.^5^ Ainda que o TCTH autólogo seja modalidade curativa para diversas patologias, a recidiva da doença de base ainda é a principal complicação pós-transplante.

## TRANSPLANTE DE CÉLULAS-TRONCO HEMATOPOÉTICAS ALOGÊNICO

O TCTH alogênico consiste na reconstituição da medula óssea por meio da infusão intravenosa de CTH de um doador aparentado ou não após a utilização de um regime de condicionamento. Este último tem por objetivo não somente erradicar as células neoplásicas por citotoxicidade direta, mas também criar um espaço imunológico no receptor, pela imunoablação e pela imunossupressão, permitindo a enxertia das CTH alogênicas.^6^

As disparidades genéticas mediadas por meio do complexo principal de histocompatibilidade (CPH), existentes entre o doador e o receptor, desencadeiam as principais reações aloimunes que acometem o pós-TCTH: a rejeição, a doença do enxerto contra o hospedeiro (DECH) e o efeito enxerto contra o tumor (ECT). Os linfócitos T do doador foram considerados os principais efetores desses dois efeitos.^7^

O efeito ECT tem papel central no TCTH alogênico, pois as células T alorreativas do doador são responsáveis por eliminar as células malignas residuais e, consequentemente, contribuem com menor risco de recaída da doença de base.^7^ Dessa forma, atualmente, considera-se que o TCTH alogênico seja uma forma de terapia celular antitumor.

## FONTE DE CÉLULAS-TRONCO HEMATOPOÉTICAS

Durante muitos anos, as CTH eram coletadas exclusivamente por múltiplas aspirações da medula óssea pela crista ilíaca posterior. No entanto, um marco importante na difusão da técnica do TCTH foi a descoberta de outras fontes de CTH, como células periféricas e SCUP.^5^

## A SELEÇÃO DO DOADOR

No TCTH alogênico, a seleção do doador com grau adequado de compatibilidade representa uma das estratégias essenciais para o sucesso do TCTH. Dentre os fatores genéticos que exercem maior influência no resultado desses transplantes estão os genes do sistema HLA.^7^

De todas as possibilidades de doadores em potencial para o TCTH alogênico, o doador HLA aparentado totalmente compatível continua contribuindo para as melhores taxas de sobrevida global e livre de progressão.^8^ Infelizmente, apenas 25% a 30% dos candidatos a TCTH alogênicos apresentam doador HLA aparentado compatível. Assim, aproximadamente 70% dos pacientes não possuem doador familiar sem incompatibilidades no sistema HLA. Existem três alternativas de fontes de CTH diante desta situação: células de doadores não aparentados cadastrados nos registros de medula óssea, células provenientes de cordão umbilical e placentário ou aquelas de doadores aparentados parcialmente compatíveis (haploidênticos).

## TIPOS DE REGIMES PREPARATIVOS (CONDICIONAMENTO)

O regime preparativo, ou de condicionamento, tem o objetivo de reduzir e até mesmo erradicar uma doença neoplásica existente, além de induzir a imunossupressão no receptor, para que seja permitida a enxertia das CTH. Na tabela 3, encontram-se os principais tipos de regimes de condicionamento utilizados no TCTH alogênico.^5^

**Tabela 3:** Tipos de regime de condicionamento

Tipos de regimes de condicionamento	Considerações
Mieloabaltivo	Altas doses de quimioterapia/radioterapia
	Mielotóxico
	Alta toxicidade
Não mieloabalativo	Objetivo principal imunoablação/imunossupressão
	Menos tóxico
Condicionamento mieloablativo de intensidade reduzida	Permite TCTH em pacientes com idade mais avançada ou com comorbidades
	Toxicidade reduzida, intermediária entre MA e NMA

Fonte: Passweg JR, Halter J, Bucher C, Gerull S, Heim D, Rovó A, et al. Hematopoietic stem cell transplantation: a review and recommendations for follow-up care for the general practitioner. Swiss Med Wkly. 2012;142:w13696. Review.^(5)^

TCTH: transplante de células-tronco hematopoéticas; MA: Mieloablativo; NMA:Não mieloablativo.

## COMPLICAÇÕES IMEDIATAS APÓS O TRANSPLANTE DE CÉLULAS-TRONCO HEMATOPOÉTICAS: TOXICIDADE, APLASIA E DOENÇA DO ENXERTO CONTRA O HOSPEDEIRO AGUDA

Após o condicionamento mieloablativo (incluindo todos os TCTH autólogos) e alguns de intensidade reduzida, observamos, depois da infusão das CTH, uma fase imediata de toxicidade em consequência da quimioterapia e/ou radioterapia administrada, que dura em torno de 10 a 15 dias. Nesta fase, os pacientes apresentam náuseas, vômitos e diarreia, sinais que podem estar associados com mucosite de grau leve à grave, ocorrendo em torno de 47% a 100% dos pacientes.^9^

A presença da mucosite depende do condicionamento recebido – a presença de irradiação corporal total, por exemplo –, da idade do paciente, do uso de metotrexato na profilaxia da DECH, da higiene oral prévia, e de alguns polimorfismos genéticos que atuam no metabolismo das quimioterapias.^9,10^ Nessa fase, os pacientes vivenciam também a toxicidade medular com aplasia e, consequentemente, o aumento da necessidade transfusional e a alta incidência infecções, principalmente bacterianas. Assim, nessa fase de alta atividade metabólica e inflamatória (chamada de síndrome de liberação de citocinas), a maioria dos pacientes não se alimenta e necessita de opioides pela dor causada pela mucosite, apresentando perda ponderal importante, caso não haja suplementação de nutrientes adequada. Esta fase desaparece principalmente com a enxertia ou a recuperação neutrofílica. Porém, neste período, aparece o risco de desenvolver a DECH aguda, que pode se manifestar na pele, no fígado e no tubo gastrintestinal. Neste último caso, o paciente pode apresentar náuseas, vômitos e diarreia, às vezes muito volumosa e, nos casos mais graves, enterorragia e melena. Novamente, o paciente apresenta um consumo metabólico importante, com necessidade de uso de corticosteroides em longo prazo, parte da terapêutica da DECH, e necessidade de jejum ou alimentação restrita.

## ❚A IMPORTÂNCIA DO ESTADO NUTRICIONAL NO TRANSPLANTE DE CÉLULAS-TRONCO HEMATOPOÉTICAS

Os pacientes submetidos ao TCTH são uma população heterogênea, do ponto de vista nutricional, e com diferenças quanto ao tipo necessário de intervenção nessa área, razão pela qual necessitam de intervenções nutricionais distintas. Devem-se levar em consideração, também, a intensidade do regime de condicionamento e a ocorrência da DECH, que influenciam diretamente no estado nutricional do paciente. A diminuição na ingesta oral, as complicações pós-TCTH e o aumento das necessidades nutricionais levam à necessidade de uma intervenção nutricional individualizada.^11^

A identificação precoce dos pacientes com risco nutricional minimiza os efeitos deletérios da desnutrição e/ou sobrepeso/obesidade. Ambos são fatores de risco para complicações e aumento da mortalidade, associada ou não à recaída em pacientes transplantados.^12^ Entretanto, uma das dificuldades que limita o impacto do peso corporal (PC) no prognóstico pós-transplante é que a maioria dos dados disponíveis são limitados ao primeiro ano após o TCTH, além da diferença entre as metodologias aplicadas e os pontos de corte utilizados para a classificação do estado nutricional.^13^ A avaliação inicial e seriada do estado nutricional é importante, pois antecipa alterações nutricionais associadas ao condicionamento e a outras toxicidades relacionadas ao tratamento, que alteram a ingesta, a absorção e a utilização de nutrientes.^11^

## ❚OBESIDADE

O excesso de peso (sobrepeso e obesidade) é um importante fator de risco para doenças cardiovasculares, *diabetes mellitus*, entre outras. A prevalência da obesidade aumenta a cada ano. Segundo dados da Organização Mundial da Saúde (OMS), em 2016, 39% da população adulta apresentava sobrepeso e 13% da população mundial, obesidade.^14^ No Brasil, não é diferente, já que dados de 2016 da Vigilância de Fatores de Risco e Proteção para Doenças Crônicas por Inquérito Telefônico (VIGITEL)^15^ demonstram que mais da metade da população está com excesso de peso e 18,9% dos brasileiros estão obesos. Assim, é cada vez mais comum pacientes com excesso de peso ou pós-cirurgia bariátrica necessitarem de transplante de medula óssea.

Diversas publicações evidenciaram a obesidade como fator prognóstico independente de resultados adversos, como aumento da mortalidade, do risco de infecções, da incidência de DECH aguda grau II-IV e toxicidade. Tarella et al.,^16^ em estudo retrospectivo com pacientes com linfoma não Hodgkin submetidos ao transplante autólogo de medula óssea, evidenciaram piores resultados, com risco de morte 2,9 vezes maior em comparação ao grupo de não obesos. Meloni et al.,^17^ também demonstraram que a obesidade é fator preditivo positivo de maior toxicidade relacionada ao tratamento e à mortalidade em pacientes com leucemia mieloide aguda (LMA) submetidos a transplante autólogo de medula óssea. A própria quantidade de células nucleadas utilizadas no transplante, menor no paciente obeso, pode influenciar nos resultados do transplante.^18^ Vogl et al.,^19^ não evidenciaram efeito do sobrepeso e nem da obesidade na sobrevida livre de progressão, sobrevida global, progressão e mortalidade relacionada à recaída em pacientes submetidos ao transplante autólogo por mieloma múltiplo. Neste mesmo estudo, os obesos submetidos ao condicionamento com melfalano e irradiação corporal total tiveram menor taxa de recaída; entretanto, a justificativa é incerta.

No entanto, em metanálise de 2014, publicada na *Bone Marrow Transplantation*, o impacto negativo da obesidade sobre os resultados do transplante de medula óssea foi estatisticamente significante no transplante alogênico e não no autólogo.^20^

Com relação à DECH, existem estudos que evidenciam o aumento da incidência de DECH agudo graus II-IV com o aumento do índice de massa corporal (IMC).^18^

A obesidade também está associada com o aumento da incidência de doenças infecciosas (bacteriana, fúngica e viral), devido ao aumento da hiperglicemia, quando comparada a pacientes com IMC menor que 30kg/m^2^.^18^

Obesos podem ter alterações na farmacocinética das drogas. Algumas delas não são solúveis em lipídios, com distribuição ruim no tecido adiposo.^17^ O *clearence* de uma droga é o parâmetro farmacocinético de maior relevância, pois se correlaciona a resultados clínicos. O fígado é o principal responsável pela metabolização, e a esteatose hepática, comum em obesos, pode ter impacto no *clearence.*^21^ Não foi evidenciada relação positiva entre obesidade, doença venoclusiva hepática e disfunção hepática nesses pacientes.^18^ O efeito renal da obesidade não está muito claro.^21^

A *American Society for Blood and Marrow Transplantation* (ASBMT), em revisão de 2014, não encontrou evidência fortemente significativa que orientasse a prescrição específica de quimioterapia para condicionamento de pacientes obesos. As sugestões, por drogas, são empíricas ou extrapolam dados de pacientes não transplantados.^22^ Entretanto, a *American Society of Clinical Oncology* (ASCO) refere que os piores resultados no tratamento quimioterápico em obesos têm causa multifatorial – no entanto, a redução inexplicada da dose dos medicamentos justifica, em parte, a mortalidade maior em pacientes com sobrepeso e obesidade. A orientação da ASCO é manter dose para o peso real do paciente.^21^

A hiperglicemia, quando ocorre na fase de neutropenia, é fator de risco para DECH aguda, estando associada ao aumento da mortalidade não relacionada à recaída. A obesidade está relacionada à hiperglicemia e à resistência à insulina. A hiperglicemia também aumenta a incidência de doenças infecciosas.^18^

A síndrome metabólica está se tornando mais prevalente nos pacientes pós-transplante. Aumento nos níveis de proteína C-reativa (PCR) e leptina, e diminuição dos níveis de adiponectinas sugerem relação com inflamação. A leptina, que é encontrada proporcionalmente à percentagem de gordura no organismo, afeta a função e a proliferação do linfócito T regulatório (TReg), suprimindo sua atividade e aumentando o risco da DECH aguda.^18^ O aumento da gordura visceral é observado nos sobreviventes.

## DESNUTRIÇÃO PROTEICO-CALÓRICA

Mesmo com a atual transição epidemiológica, ainda existe percentual considerável de pacientes com sarcopenia,^23^ desnutrição proteico-calórica e em risco nutricional.^24^

Há décadas, sabe-se que a desnutrição afeta negativamente o resultado do TCTH. Um estudo restrospectivo com 2.238 pacientes submetidos ao transplante autológo e alogênico encontrou associação significativa entre perda de peso após 150 dias do TCTH e pior prognóstico.^28^

Um estudo que avaliou 544 pacientes adultos submetidos ao transplante alogênico encontrou maior mortalidade por infecção e por recidiva da doença nos indivíduos com IMC inferior a 20kg/m^2^.^29^ Perda de peso superior a 10% também está associada à maior taxa de mortalidade pós-transplante alogênico.^26^

Em um estágio de desnutrição proteico-calórica, de perda involuntária de peso e de redução de proteínas plasmáticas, como a albumina, há risco elevado de toxicidade à quimioterapia e às demais classes de medimentos administrados.^30^ A albumina é a proteína mais abundante do soro humano, desempenha diversas funções na homeostase do organismo e possui papel essencial como proteína de transporte de drogas. Isso pode ter implicação farmacocinética na terapia clínica, tendo consequência nas concentrações plasmáticas dos quimioterápicos.^31^

A hipoalbuminemia pré-TCTH impacta significativamente na sobrevida desses pacientes. Doentes com níveis de albumina inferiores a 3,2g/dL apresentaram sobrevida livre de doença significativamente mais baixa do que aqueles com nível de albumina superior a 3,2g/dL.^32^ O baixo PC também reduz a tolerância ao tratamento, contribuindo para maior toxicidade à quimioterapia, por alterar a farmacocinética das drogas.^33^

Outro grande prejuízo que o comprometimento do estado nutricional pode trazer ao indivíduo que se submete ao TCTH é a maior duração da aplasia durante o transplante, na qual o baixo IMC, a hipoalbuminemia e o aumento da excreção de nitrogênio urinário são associados ao maior tempo para enxertia neutofílica, o que acarreta aumento da suscetibilidade a infecções,^34^ além de trazer prejuízos financeiros, pois estes pacientes costumam apresentar internação hospitalar prolongada.^30^

Além dos danos já esperados, a desnutrição proteico-calórica e a baixa ingestão oral são associadas ao aumento da DECH^35^ − em especial a DECH de pulmão, trato gastrintestinal e cavidade oral. A deficiência de vitaminas A e D também é associada ao aumento da incidência e da gravidade do DECH.^36^

Nesse sentido, recomenda-se a realização da avaliação nutricional pré-TCTH até 30 dias antes da internação para o TCTH, com o objetivo de recuperar ou manter o estado nutricional pré-TCTH e corrigir carências nutricionais, buscando minimizar os efeitos deletérios do TCTH no estado nutricional.

Por meio da avaliação do estado nutricional, de alterações na ingestão alimentar e da semiologia nutricional, podem ser definidos grupos de maior risco, possibilitando intervenção nutricional rápida e eficaz, para redução do catabolismo proteico.^37^

## ❚AVALIAÇÃO NUTRICIONAL

Pacientes submetidos ao TCTH devem ser triados assim que admitidos para o procedimento ou em qualquer internação relacionada a complicações do tratamento ou dos regimes de condicionamento realizados. Normalmente, são considerados de risco nutricional ou já estão desnutridos em função da doença de base,^38^ do regime quimioterápico realizado e da toxicidade do tratamento. Os diferentes agentes citotóxicos, a radioterapia e outros novos fármacos utilizados no tratamento onco-hematológico afetam não apenas as células tumorais, mas também as células saudáveis, especialmente aquelas com alta taxa de replicação, como é o caso dos linfócitos e das células do trato gastrintestinal (enterócitos e colonócitos). Os efeitos sobre estas células resultam em grandes alterações funcionais do aparelho digestivo e do sistema imunológico, levando à má absorção e comprometendo seriamente o estado nutricional do paciente.^39,40^ Os regimes de condicionamento de intensidade reduzida (RIC - *reduced-intensity conditioning*) são indicados, principalmente, para pacientes idosos ou com múltiplas comorbidades, o que contraindicaria o TCTH mieloablativo. O RIC reduz os efeitos colaterais, como mucosite e menor tempo de neutropenia.^41,42^ O TCTH alogênico tem considerável toxicidade e leva a uma resposta inflamatória importante, além de mudanças metabólicas (como caquexia), sintomas gastrintestinais e efeitos generalizados, que podem levar à redução do consumo alimentar e à piora do estado nutricional. Esse comportamento claramente coloca os pacientes em risco nutricional, o que deve, adicionalmente ao tratamento, influenciar, de forma negativa, os desfechos clínicos.^43^

Considerando-se uma doença crônica, como o câncer, e os fatores que podem desfavorecer a condição do doente ou a resposta clínica ao tratamento (como a idade e o fator psicossocial, por exemplo), há grande chance de prejuízo do estado nutricional em fases importantes do tratamento − principalmente durante o TCTH. Alguns diagnósticos consideram a perda de peso, a massa muscular e o processo inflamatório, e caracterizam, em fases diversas, a presença de caquexia (pré-caquexia ou caquexia refratária).

Dentre 503 pacientes avaliados na Alemanha^44^ em relação à presença de caquexia considerando os critérios de Fearon et al.,^45^ (perda de peso <5% durante os últimos 6 meses, ou perda de peso de 2 a 5%, aliada a IMC <20kg/m^2^, ou perda de peso de 2 a 5% somada à presença de sarcopenia), encontrou-se incidência de 131 pacientes com diagnóstico de caquexia, e, destes, 15,2% eram pacientes hematológicos. Os autores demonstraram que pacientes com caquexia possuíam múltiplos sintomas, como anemia e prejuízo das funções renal e hepática (colestase).

No entanto, para efeito de padronização de discussão, abordaremos apenas o diagnóstico de desnutrição ou baixo peso, segundo medidas antropométricas.

A avaliação do estado nutricional com o instrumento validado antes, durante e depois do TCTH ainda é pouco discutida e documentada, mas, certamente, deve ser realizada. Não existe consenso, na literatura, que diferencia a forma de avaliação nutricional para cada fase, mas é fato que a avaliação deve acontecer de acordo com o protocolo de cada instituição e englobando as principais fases do TCTH: admissão; início do regime quimioterápico de preparação; dia da infusão das células-tronco hematopoiéticas; início dos imunossupressores, alta hospitalar, 1 mês e 3 meses após o TCTH; 6 meses e 1 ano após o TCTH alogênico (em nível ambulatorial).

A *American Society Parenteral and Enteral Nutrition* (ASPEN)^40^ e a *European Society Parenteral and Enteral Nutrition* (ESPEN),^46^ em seus consensos, recomendam triagem nutricional e intervenção nutricional se o paciente for incapaz de manter seu estado nutricional. O último consenso da ESPEN para pacientes oncológicos, de 2017,^47^ recomenda, para detecção de distúrbios nutricionais em estágio precoce, que sejam avaliados regularmente o consumo alimentar, a mudança de peso e o IMC, a começar no diagnóstico oncológico, sendo revistos de acordo com a estabilidade clínica do paciente. Orienta que, para tanto, sejam utilizados parâmetros como IMC, perda de peso e índice de registro alimentar, ou a utilização de um instrumento de triagem validado como o *Nutritional Risk Screening* (NRS-2002), *Malnutrition Universal Screaning Tool* (MUST), *Malnutrition Screening Tool* (MST) *or Mini Nutritional Assessment Short Form* (MNA-SF). É importante notar que um resultado anormal (presença de risco) de triagem, por si só, não fornece informações suficientes para desenhar um plano de cuidado ao paciente. Pacientes em risco precisam ser seguidos e avaliados por instrumentos mais específicos para, então, uma intervenção ser desenhada. Assim, a ESPEN recomenda fortemente que, após identificação do risco, o paciente seja submetido a uma avaliação objetiva e quantitativa acerca de consumo alimentar, impacto de sintomas, massa muscular, desempenho físico e grau de inflamação sistêmica. Recomenda também que essa avaliação seja repetida em períodos frequentes (por exemplo, quinzenalmente, mensalmente, a cada 6 meses, conforme apropriado). Os instrumentos citados para essa etapa são: Avaliação Subjetiva Global (ASG), Avaliação Subjetiva Global Produzida pelo Paciente (ASG-PPP) ou Miniavaliação Nutricional (MAN).

O que mais preocupa a equipe assistencial e os gestores responsáveis por unidades de TCTH é o fato de o risco nutricional estar associado ao aumento de mortalidade e dos custos de hospitalização.^48^

Assim, é primordial, no cuidado ao receptor do TCTH, que a triagem nutricional e a avaliação nutricional sejam realizadas na admissão hospitalar, e em suporte ambulatorial anterior à internação, em um preparo para o TCTH. A periodicidade deve ser definida com base na presença ou não do risco nutricional, devendo não exceder 15 dias para pacientes ambulatoriais já sob risco nutricional e 30 dias para aqueles que ainda não apresentaram o risco. Os pacientes hospitalizados devem ser triados em até 48 horas após a internação, sendo reavaliados em 7 dias. Essa reavaliação semanal deve ser conduzida até a alta hospitalar.^40,49^

O tempo de triagem pode ser alterado, a depender de critérios de qualidade em cuidados da assistência nutricional. A triagem de risco nutricional realizada até 24 horas da admissão hospitalar é uma das exigências do processo de auditoria de qualidade, como a *Joint Commission International* (JCI).^50^

Ainda não se tem definido qual é o melhor instrumento para reavaliar o doente oncológico. Muitos não são válidos para reavaliações. Porém, de fato, o doente onco-hematológico que será submetido ao TCTH deve ser acompanhado e reavaliado periodicamente. As visitas nutricionais possibilitam intervenções precoces e importantes para cada fase do tratamento, mas a mudança do estado nutricional ou funcional deve ser diagnosticada. Os instrumentos que possibilitam escores de diagnóstico final podem ser alternativos para avaliar o paciente em diversos momentos. A avaliação funcional ou a de qualidade de vida, nesse sentido, podem ser instrumentos importantes para nortear mudanças no decorrer do tratamento (bons exemplos podem ser ASG-PPP e dinamometria para avaliação funcional da força muscular).

## INSTRUMENTOS PARA AVALIAÇÃO NUTRICIONAL

### NRS-2002

O instrumento mais indicado para avaliar o risco nutricional em pacientes hospitalizados é o NRS-2002, que considera a idade do paciente (acrescentada à pontuação final da triagem), inclui todos os pacientes clínicos e cirúrgicos,^51^ e é recomendado pela ESPEN para triagem em hospitais. Foi validado em uma análise de 128 ensaios controlados randomizados de suporte nutricional, para avaliar se poderia distinguir pacientes com desfecho clínico positivo devido à intervenção nutricional daqueles que não mostraram benefício com a Terapia Nutricional (TN).^52^ O NRS-2002 é o primeiro instrumento de triagem nutricional desenvolvido com base na medicina pautada em evidência.^51,53^

Além disso, tem mostrado ter alta validade preditiva e baixa variação interobservador (k=0,67).^53^ O risco nutricional é avaliado pela combinação de estado nutricional atual e da gravidade da doença, sendo o primeiro composto pelas variáveis IMC, perda de peso recente e ingestão dietética durante a última semana antes da admissão hospitalar.

Estudo nacional na Suíça^24^ em centros de TCTH que somaram 621 transplantes realizados em 2014 (226 alogênicos e 395 autólogos) observou que todos os centros envolvidos em TCTH alogênico (n=3) realizavam triagem nutricional usando principalmente o NRS-2002. Em apenas um deles, a triagem foi desenvolvida pelo próprio centro, com incorporação de parâmetros de calorimetria indireta (CI) e bioimpedância elétrica. Quanto aos centros de TCTH autólogo (n=7), somente metade deles possuía protocolo de triagem nutricional. Três centros acionaram o nutricionista apenas se existisse suspeita de desnutrição durante a internação.

Liu et al.,^54^ investigaram, em 99 pacientes chineses com leucemia, se o NRS-2002 é capaz de avaliar adequadamente o risco, antes e após o TCTH, e se há diferenças com relação à idade e ao sexo. A média de idade dos pacientes era de 32,4 anos, e o tempo médio em quarto de fluxo laminar era de 30 dias. Dois nutricionistas ficaram responsáveis pela aplicação do instrumento no dia da internação (antes do TCTH) e no dia da conclusão do TCTH (depois). Dos pacientes triados, 22,2% apresentavam risco nutricional. Ao mesmo tempo, 15,4% dos pacientes com IMC ≥18,5kg/m^2^ tinham risco nutricional, apontando que o IMC sozinho não poderia, acertadamente, refletir se há ou não risco nutricional. Os pacientes foram agrupados de acordo com sexo, idade e outras condições, e a incidência de risco foi comparada. Os autores mostraram que não há diferença significativa na incidência de risco de acordo com o sexo; no entanto, há diferença para idade, grau de compatibilidade (pacientes com menos de 30 anos e não “*full-matched*” possuem maior risco nutricional), perda de peso e redução da ingestão alimentar ou do IMC (p<0,01). Todos os pacientes foram identificados como risco nutricional após o TCTH, visto que só o fato de realizar o transplante, segundo autores, já confere escore 3 na gravidade da doença (ou seja, risco nutricional), mostrando que o NRS-2002 não deve ser instrumento apropriado nessa fase. No entanto, os autores destacam que 63,6% dos pacientes possuíam escore 6 (o máximo, a considerar que não havia nenhum paciente com mais de 70 anos na amostra), o que significa que, embora o NRS-2002 não distinga risco após o TCTH, os pacientes tiveram escore mais alto que antes do procedimento. Além disso, 77,8% dos pacientes perderam peso pós-TCTH e, dentre eles, 63,6% tinham perda >5% em um mês.^54^

### Avaliação Subjetiva Global e Avaliação Subjetiva Global preenchida pelo próprio paciente

A ASG é um instrumento frequentemente utilizado para avaliar o estado nutricional de pacientes hospitalizados. Trata-se de método clínico, desenvolvido por Detsky et al.,^55^ capaz de avaliar o risco nutricional e, principalmente, identificar pacientes que necessitam de TN mais específica.^55^

A ASG, quando aplicada sequencialmente após o NRS-2002, aumenta a sensibilidade e a especificidade dos dois testes na predição de desfechos clínicos negativos. Assim, a TN pode ser direcionada para pacientes efetivamente em débito nutricional.^56^

A ASG apresentou associação com os escores *Acute Physiology and Chronic Health Evaluation II* (APACHE II) e *Simplified Acute Physiology Score* II (SAPS II) e com mortalidade em coorte descritiva de 124 pacientes críticos clínicos e cirúrgicos.^57^

A limitação do método ASG é o fato de ser, como o próprio nome diz, subjetiva, dependendo da experiência do observador.

Em 1995, foi validada a utilização de uma ASG adaptada para pacientes oncológicos: a ASG-PPP. Consiste em questionário autoaplicativo, dividido em duas partes. Na primeira parte, o próprio paciente responde às questões, descrevendo alteração de peso, ingestão alimentar, sintomas relacionados ao câncer e a alterações da capacidade funcional. Na segunda parte, respondida pelo profissional que aplica o questionário, as questões são baseadas em fatores associados ao diagnóstico que aumentam a demanda metabólica, como estresse, febre, depressão, fadiga, estadiamento do tumor ou tratamento, e o exame físico.^58^ Um ponto negativo deste método é o fato de que alguns pacientes podem encontrar dificuldades para responder às questões relacionadas à perda de peso nos últimos meses, assim como para especificar a ingestão alimentar durante o último mês, o que pode alterar, em alguns casos, a classificação do estado nutricional.^59^

A ASG-PPP também tem como vantagem o paciente se sentir mais participativo e otimiza o tempo gasto pelo profissional para finalizar a avaliação. Mostra-se adequada para identificar pacientes oncológicos que se beneficiariam de intervenção nutricional preventiva durante a terapêutica oncológica.^60,61^ As limitações decorrem da incompreensão do paciente para responder aos questionamentos relacionados à perda de peso, bem como para especificar a ingestão alimentar.^60^ A ASG-PPP apresenta alta sensibilidade e especificidade, com valores de 80% e 89%, respectivamente.^62^

Barritta de Defranchi et al.,^63^ por meio de estudo de coorte prospectivo, longitudinal, descreveram e compararam o estado nutricional de 123 pacientes sob TCTH, admitidos em hospital universitário em Buenos Aires, em três diferentes momentos: em até 24 horas da admissão para transplante (normalmente 3 a 7 dias antes do transplante para quimioterapia), na alta e em consulta de seguimento (10 dias após a alta). O estado nutricional foi avaliado pelo IMC e pela ASG-PPP. Do total de pacientes, 36 apresentavam dados falhos em algumas das fases de avaliação do estudo, por diversos motivos, como reinternação, transferência para UTI e até mesmo óbito. A média de idade é de 50,5 anos. Mieloma múltiplo foi o diagnóstico mais frequente (44,8%). A maioria (80,5%) foi submetida ao TCTH autólogo. Os autores encontram significativa diferença entre o estado nutricional na admissão, alta e seguimento: os escores médios da ASG-PPP eram 3,39 na admissão, 12,81 na alta e 6,71 no acompanhamento ambulatorial (p<0,001). A maioria dos pacientes admitidos (94,3%) era bem nutrida na admissão. Se considerado o IMC, não existia paciente com baixo peso na amostra, e a maioria apresentava sobrepeso (34,1%) ou era obesa (33,3%). Na alta, 59,7% estavam desnutridos (ASG-PPP B e C). Pacientes com tempo de internação hospitalar maior que 21 dias tiveram 2,9 pontos a mais no escore da ASG-PPP que pacientes com tempo de internação menor que 21 dias (p=0,034). Pacientes com idade inferior a 60 anos tinham pior escore da ASG-PPP (p=0,0007). Pacientes submetidos ao TCTH alogênico tiveram escore mais alto na alta hospitalar (ou seja, pior desfecho) que aqueles submetidos ao TCTH autólogo (p=0,0152). Na avaliação ambulatorial, o escore da ASG-PPP ainda é mais alto que o escore da admissão (3,66 pontos a mais). Grande parcela de pacientes (74,7%) tinha escore ≥4, mostrando necessidade de intervenção nutricional imediata. O PC aumentou da alta para a consulta (média de 74,5kg para 75,4kg), mas também continuava menor que o peso da admissão (média de 78kg).

### Malnutrition Universal Screeaning Tool

O instrumento *Malnutrition Universal Screeaning Tool* de triagem universal de desnutrição é utilizado principalmente na desnutrição proteico-calórica, que inclui três parâmetros clínicos (IMC, perda de peso involuntária nos últimos 3 a 6 meses, doença aguda ou jejum superior a 5 dias) e atribui a cada item uma pontuação de zero, 1 ou 2, conforme a descrição a seguir:

- IMC >20kg/m^2^, pontuação igual a zero; 18,5-20kg/m^2^, pontuação igual a 1; abaixo de 18,5kg/m^2^, pontuação igual a 2;
- Perda ponderal menor que 5%, pontuação igual a zero; 5% a 10%, pontuação igual a 1; maior que 10%, pontuação igual a 2;
- Doença aguda ou jejum superior a 5 dias, se ausente, pontuação igual a zero; se presente, pontuação igual a 2.

Após a realização dos primeiros três passos, somam-se todas as pontuações para calcular um risco geral de desnutrição. Um escore maior que 2 pontos classifica o paciente em alto risco de desnutrição; escore igual a 1 ponto, em médio risco de desnutrição; e escore igual a zero, em baixo risco de desnutrição. Para cada categoria de risco nutricional, são oferecidas linhas de orientação, que auxiliam o profissional da saúde como agir a partir dos resultados propostos.^64,65^

### ANTROPOMETRIA

A desnutrição oncológica envolve histórico pregresso e contínuo de *deficits* e provoca alterações na composição corporal, incluindo perda de gordura corporal e massa magra, que resultam em perda de peso e mudanças em outros parâmetros antropométricos.^66^

As medidas antropométricas apresentam grande importância no estabelecimento de fatores determinantes da desnutrição e do sobrepeso, dentre outros fatores, como instrumento de vigilância nutricional.^67^

Entre as medidas antropométricas mais utilizadas na prática clínica para avaliação do estado nutricional dos pacientes oncológicos, podemos citar PC, altura, IMC, espessura de dobras cutâneas, circunferência do braço (CB) e circunferência muscular do braço (CMB).^68^ No entanto, na prática clínica, em virtude de algumas restrições de mobilidade, presença de acessos, edema ou imobilidade, o uso de tais parâmetros nem sempre é possível. A inclusão em protocolos deve ser avaliada com cautela, até mesmo para considerar o diagnóstico encontrado frente a outros instrumentos utilizados (como avaliações subjetivas). Uma interessante opção é usar um indicador antropométrico para comparação de mudanças ao longo do tempo.

A antropometria é o método mais difundido, por não ser invasivo, de fácil execução, rápido, de baixo custo, factível à beira do leito e de resultados fidedignos, desde que executados por profissionais capacitados. Como desvantagem, é incapaz de detectar distúrbios recentes no estado nutricional e identificar deficiências nutricionais específicas. De 226 pacientes adultos ambulatoriais oncológicos acompanhados em um estudo espanhol, 64% apresentavam desnutrição (avaliada pela ASG-PPP), e o IMC não foi de utilidade para tomada de decisão de TN, já que metade da população (56,5%) apresentava IMC adequado, embora tenha sido observado que, à medida que diminuía o IMC, aumentavam as dificuldades de alimentação.^69^

## PESO E ÍNDICE DE MASSA CORPORAL

O peso é a soma dos compartimentos corporais e reflete o equilíbrio proteico energético do indivíduo. Para mensuração do peso, é necessário o uso de uma balança (plataforma ou eletrônica) com precisão de 100g. Ela deve estar instalada em local nivelado; o indivíduo deve se posicionar no centro do equipamento, com o mínimo de roupa possível, descalço, ereto, com os pés juntos e os braços estendidos ao longo do corpo.^70^

O peso deveria ser corrigido para excesso de fluidos (como edema pleural, ascite e/ou edema de qualquer natureza).^47^

Pesquisadores japoneses avaliaram 48 pacientes com linfoma ou mieloma submetidos ao TCTH autólogo em hospital universitário, com relação à presença de disgeusia avaliada via entrevista (divididos em pacientes que apresentaram disgeusia durante o tratamento – 42% – e pacientes que não apresentaram – 58%). O uso de crioterapia com flocos de gelo antes do melfalano (total de 120 minutos a cada regime) foi significativamente menor no grupo que apresentou disgeusia. Análises univariadas e multivariadas de regressão logística mostraram que o esquema quimioterápico utilizado e a presença de mucosite oral foram fatores de risco independentes para disgeusia, enquanto a crioterapia foi fator supressor independente. Não houve diferença na perda de peso entre os grupos; porém, ambos perderam cerca de 4kg depois do TCTH autólogo.^71^

Aoyama et al.,^72^ avaliaram 51 pacientes (média de idade de 51 anos) submetidos a TCTH alogênico em um departamento de transplante de células-tronco no Japão e relação de perda de peso com algumas variáveis controladas no fluxograma de indicação de TN no hospital da pesquisa. Os itens foram avaliados de 1 a 2 dias antes do início do pré-tratamento e no final da TN parenteral (TNP; que pode ser indicada de forma complementar), até o dia 100. Os pacientes foram divididos em dois grupos: aqueles que perderam ≥7,5% do peso nos últimos 3 meses e aqueles com perda de <7,5% no mesmo período. Os autores observaram forte e significativa correlação (r=0,89; p<0,0001) entre as variações de peso e mudanças na composição corporal (avaliado por bioimpedância). No entanto, uma fraca correlação foi observada entre variações de peso e mudanças de massa gordurosa (r=0,42; p=0,002). O IMC médio antes do início do pré-tratamento era de 22,1kg/m^2^, e a perda de peso, de 4,5%. A DECH aguda ocorreu mais frequentemente, e de forma mais grave nos pacientes com perda de peso ≥7,5%, sugerindo que o estado nutricional deve afetar o desfecho clínico pós-TCTH. Também nesse grupo foram observados escores mais altos para eventos adversos relacionados à nutrição e por período mais longo. Os autores apontam que, no estudo, os eventos adversos relacionados à nutrição (como náuseas, mucosite, estomatite, alteração do paladar, anorexia e vômitos) foram associados com aumento na graduação de performance status, e seria interessante que intervenções nutricionais considerassem a gravidade dos eventos adversos citados.

Yang et al.,^73^ em estudo de coorte retrospectivo, avaliaram 267 pacientes chineses (66,7% com idade ≤40 anos) com diagnóstico de LMA ou leucemia linfóide aguda (LLA), que receberam radiação de corpo inteiro ou bussulfano + ciclofosfamida, como regimes de condicionamento. Os pacientes foram seguidos periodicamente, após o TCTH, até a morte ou último seguimento – 13 anos de acompanhamento (média de seguimento do grupo de 19,7 meses). Os grupos de IMC foram reunidos em pacientes com menor IMC (baixo peso e eutrofia, ou seja, segundo critérios chineses de avaliação, IMC <23kg/m^2^) e maior IMC (sobrepeso e obesidade). Do total, 9,7% possuíam baixo peso; 51,3%, eutrofia; 16,9%, sobrepeso; e 22,1%, obesidade. Não houve diferença entre os grupos de IMC, exceto pela idade (> e ≤40 anos). Um total de 93 pacientes (34,8%) morreu durante o seguimento do estudo. A probabilidade de sobrevida global foi pior no grupo de menor IMC, comparado ao de maior IMC (IMC ≥23kg/m^2^), com p=0,041. Após 3 anos do TCTH, a probabilidade de sobrevida global era de 55,7% para o grupo de menor IMC e 72,3% para o grupo de maior IMC. Comparado com o baixo peso, os pacientes eutróficos, com sobrepeso ou obesidade mostraram menor razão de chance (RC), com significativa tendência de aumento da sobrevida global com o aumento do IMC (p=0,019), após ajuste para idade, sexo, diagnóstico, estado da doença, tipo de enxerto, regime de condicionamento, regime de profilaxia da DECH e tempo do diagnóstico ao transplante. Além disso, pacientes com mais alto IMC sobreviveram mais tempo, com diminuição significativa da RC por volta de 40% comparada com o grupo de baixo IMC. Os autores acreditam que a diferença na sobrevida encontrada no estudo entre os IMC (maior e menor) deve ser atribuída à diferença no metabolismo de quimioterápicos usados em regimes de condicionamento. Pacientes com sobrepeso e obesos possivelmente têm mais altas doses cumulativas de drogas ou tempos maiores de exposição, o que pode resultar em melhores desfechos.

Jaime-Pérez et al.,^74^ analisaram 77 pacientes com doenças hematológicas malignas ou benignas submetidos a TCTH 6/6 HLA compatíveis (64 alogênico e 13 autólogo), em modelo de tratamento ambulatorial, tratados em hospital universitário, no México. O programa de condicionamento consistia em esquema RIC com dosagem baseada no peso ideal. Não houve diferença na mortalidade entre pacientes com IMC <18 e ≥18kg/m^2^). As alterações de IMC foram registradas a cada visita ou, ao menos, mensalmente. Os dados foram analisados nos primeiros 6 meses após o TCTH e, depois, a cada 3 meses, até completar 1 ano. Considerando o IMC, 18,2% possuíam baixo peso; 24,7% tinham peso normal; 33,7% apresentavam sobrepeso e 23,4% eram obesos. Houve significativa diferença no peso do paciente antes (média de 68,3kg) e após o TCTH (média de 56,3kg), com p=0,014, mas não na percentagem de gordura ou no IMC (p=0,458 e p=0,067, respectivamente). Não houve correlação entre IMC > ou <30 e tempo para pega medular (p=0,404) ou tempo de neutropenia – neutrófilos <500x10^9^/L (p=0,014). Analisando a curva de Kaplan-Meier de sobrevida global, não houve diferença entre os subgrupos de IMC e sobrevida (p=0,059), mas, quando a sobrevida era comparada entre pacientes obesos (n=18) e o restante do grupo, sobrevida maior foi observada nos pacientes obesos (p=0,017), tanto para TCTH autólogo quanto para alogênico. Não houve relação entre IMC e desenvolvimento da DECH no grupo de TCTH alogênico analisado.

Espinoza et al.,^75^ avaliaram 18 pacientes submetidos ao TCTH autólogo e 32 ao TCTH alogênico, no Chile, com média de idade de 41 anos, 84% com condicionamento mieloablativo e 16% de RIC. A avaliação nutricional foi realizada no momento do TCTH, 10 dias após e no momento da alta. O tempo médio de permanência hospitalar foi de 32 dias e a média de seguimento, de 41 meses. Na admissão, não havia diferença significativa com relação a parâmetros nutricionais entre o grupo autólogo e o grupo alogênico, e somente no grupo autólogo houve alteração significativa do IMC após o TCTH (27,3kg/m^2^ para 26,3kg/m^2^). Considerando ambos os tipos, houve redução do IMC no pós-TCTH comparado ao pré (26,9kg/m^2^ para 26,1kg/m^2^), com p<0,01.

## DINAMOMETRIA

A capacidade muscular de indivíduos desnutridos encontra-se significativamente diminuída, uma vez que a desnutrição proteico-calórica afeta diretamente a perda de todas as fibras musculares esqueléticas e, consequentemente, diminui a força muscular.^76^ A dinamometria manual, ou força do aperto de mão, é um método utilizado na avaliação nutricional dos pacientes, por ser um marcador de prognóstico, inclusive em pacientes oncológicos. É um método de fácil aplicabilidade, simples, rápido e de baixo custo, além de prever o status funcional muscular.^77^ A dinamometria é indicada como complementação da triagem nutricional.^49^

Os aparelhos utilizados para esta medida de força podem ser classificados em quatro categorias: hidráulicos, pneumáticos, mecânicos e *strain gauges* (ou células de carga).^78^

Pastore et al.,^79^ avaliaram o impacto do estado nutricional e da força muscular na qualidade de vida de 77 pacientes no sul do Brasil com câncer do trato gastrintestinal e de pulmão, indicados à quimioterapia pela primeira vez. Os pacientes tiveram o estado nutricional avaliado pelo IMC e ASG, e a função muscular avaliada por dinamometria (dinamômetro mecânico Jamar). Foram obtidas três medidas de cada mão (dominante e não dominante), e foi utilizada a maior medida. O domínio de estado de saúde geral e qualidade de vida foi avaliado pelo questionário *European Organization for Research and Treatment of Cancer – Quality of Life Questionnaire Core-30* (EORTC QLQ-C30). Segundo o IMC, 60% da amostra estava estrófica; porém, a ASG encontrou somente 13% de bem nutridos. O escore de qualidade de vida médio foi 68±21,3. A média da força de preensão manual não dominante foi 25,7±10,1Kgf, sem associação com qualidade de vida (p=0,3). O pior estado nutricional associou-se com menores escores de qualidade de vida, tanto pelo IMC (p=0,04) quanto pela ASG (p=0,007). Houve alta prevalência de desnutrição, segundo a ASG, e o estado nutricional influenciou significativamente no estado de saúde geral e na qualidade de vida, porém não foi observada relação entre estado nutricional e força muscular.

Estudo realizado na Suíça^80^ com 37 pacientes com câncer de pulmão não ressecável observou que a dinamometria manual pode ser instrumento útil para avaliar o estado funcional e nutricional. Pode ser incluída na avaliação de pacientes com câncer juntamente de outros instrumentos de avaliação nutricional. O estado nutricional foi obtido por meio da ASG-PPP, e a função muscular foi avaliada utilizando dinamômetro de mão Jamar na mão não dominante. Foram obtidas três medidas, e foi utilizada a maior delas. Segundo a ASG-PPP, 73% (n=27) dos pacientes estavam moderadamente desnutridos, e 8% (n=3) estavam gravemente desnutridos. No total, 81% (n=30) estavam desnutridos. Força do aperto de mão estava abaixo do percentil 50 em 57% dos pacientes (n=21). Houve associação significativa entre o estado nutricional e força do aperto de mão (p=0,026).

O estudo de Tanaka et al.,^81^ avaliou 34 pacientes com doença hematológica submetidos a TCTH alogênico em hospital no Japão. O regime quimioterápico foi mieloablativo para 14 pacientes e não mieloablativo para 20. Foi instituído programa de reabilitação realizado cinco vezes por semana, 7 dias antes do transplante, consistindo no treinamento de resistência e em exercícios aeróbicos, 20 minutos por dia. O paciente foi acompanhado até a descontinuidade da infusão contínua de imunossupressores (aproximadamente 30 dias após o transplante). Além de testes para avaliação da função física (entre eles, a força da preensão palmar), o estado nutricional foi avaliado em cinco momentos: na admissão, no início do regime quimioterapia de preparação, no dia do transplante, quando os imunossupressores foram iniciados e na alta. O estado nutricional foi analisado avaliando-se consumo calórico, alteração de peso, albumina, PCR e circunferências (para medida muscular). Sarcopenia, para o grupo de pacientes, foi definida conforme os critérios da *European Working Group on Sarcopenia in Older People* (EWGSOP).^82^ O peso, após o TCTH, foi significativamente menor que o peso antes do TCTH (58,6±15,6kg e 56,0±14,5kg), p=0,001. A força da preensão palmar e a circunferência dos membros superiores e inferiores diminuíram, bilateralmente, e de forma significativa, após o TCTH. A força muscular diminuiu significativamente em 6%, um mês após o TCTH, mas foi recuperada 3 meses depois. No entanto, para os pacientes com DECH aguda que precisaram de esteroides, a perda muscular foi em torno de 16% ao longo de 3 meses pós-TCTH. Análise multivariada mostrou que o consumo calórico via oral após o TCTH foi fator significativo associado à perda de peso. Os autores concluem que o peso foi indicador sensível de sarcopenia; no entanto, a circunferência não se apresentou como melhor método para avaliar a força muscular. No estudo em questão, a circunferência variou significativamente após o TCTH, mas a redução foi pequena. De qualquer forma, pacientes com sarcopenia antes do TCTH estão mais suscetíveis a eventos adversos do que pacientes sem sarcopenia − por isso a importância desta avaliação.

## COMPARATIVOS

Liu et al.,^25^ avaliaram o estado nutricional de 170 pacientes com doença hematológica em Pequim submetidos a TCTH alogênico, utilizando parâmetros de antropometria e quatro instrumentos: NRS-2002, MAN, ASG e MUST. O grupo foi composto por 116 homens e 54 mulheres, com média de idade de 30 anos (12±56), 65 casos de LMA, 63 casos LLA, 14 casos leucemia mieloide crônica (LMC), 3 linfomas, 3 anemias aplásicas e 22 síndromes mielodisplásicas. Os pacientes foram avaliados antes de serem submetidos ao TCTH e entrarem em quartos de fluxo laminar, e em até 48 horas após deixarem o quarto (o tempo médio de permanência foi de 30 dias). Apenas 109 pacientes foram avaliados no dia que saíram do quarto. As avaliações foram realizadas por dois nutricionistas. O tempo de tratamento foi de 30 (±2,1) dias. Após o TCTH, os pacientes tiveram redução significativa no peso, circunferência do quadril, relação cintura-quadril, circunferência da panturrilha, CB, com relação a medidas pré-TCTH. Antes do TCTH, a NRS-2002 identificou que 21,2% dos pacientes estavam em risco nutricional, em comparação com 100% após o TCTH. O MUST indicou que, antes do TCTH, 11,8% dos pacientes apresentavam alto risco nutricional, comparado com 59,6% pós-TCTH. A MAN avaliou que 0,06% dos pacientes estavam desnutridos antes do TCTH, comparado com 19,3% após o TCTH. Avaliação Subjetiva Global identificou que, antes do TCTH, 1,76% dos pacientes apresentavam desnutrição leve a grave, proporção que aumentou para 83,3% pós-TCTH. Os autores concluem que há aumento significativo no risco nutricional e desnutrição em pacientes que receberam TCTH. A análise comparativa da triagem nutricional no pós-transplante usando MUST revelou resultados significativamente diferentes daqueles obtidos com NRS-2002. A ASG indicou que 83,3% dos pacientes apresentavam desnutrição moderada a grave, enquanto o MAN avaliou 19,3% dos pacientes como afetados pela desnutrição, o que provavelmente se deve à subjetividade da ASG, levando a aumento da probabilidade de desnutrição quando este instrumento é o escolhido. Independentemente do método de avaliação, as evidências mostram que os pacientes com doenças hematológicas sofrem de estado nutricional significativamente deteriorado após o transplante e apresentam risco nutricional relativamente elevado.

Em estudo prospectivo com 108 pacientes com leucemia após o TCTH, Wang et al.,^83^ investigaram o estado nutricional utilizando diferentes métodos de avaliação (NRS-2002, MAN, MUST e ASG). Participaram do estudo 77 homens e 31 mulheres, com média de idade de 8 (±56) anos, 61 casos de LLA, 15 casos de LMA, 14 casos de LMC e 19 casos de síndrome mielodisplásica (SMD). Um total de 108 pacientes completou ASG e 99 pacientes completaram NRS-2002, MAN e MUST. Durante o processo de tratamento, 85,2% dos pacientes perderam peso, sendo que 50% deles perderam peso superior a 5%, e 42,6% tinham reduzido significativamente a ingestão de alimentos. Para avaliação do risco nutricional, as taxas positivas de risco usando NRS-2002, MAN e MUST foram de 100%, 74,7% e 63,6%, respectivamente. Houve diferença significativa (p<0,05) entre NRS-2002, MAN e MUST. Na avaliação da desnutrição, a taxa positiva de ASG (83,3%) foi significativamente maior que a do MAN (17,2%), com p<0,05, e a incidência de risco nutricional entre pacientes com leucemia ≤30 anos foi maior do que a dos pacientes >30 anos (p<0,05). Os autores sugerem que NRS-2002 pode ser usado para avaliação de deficiências nutricionais, mas sua especificidade não é alta. A MAN é aplicável à triagem do risco nutricional no idoso, mas não é adequada para avaliação nutricional. Assim, a combinação de diferentes instrumentos de triagem e indicadores laboratoriais clínicos seria o mais interessante para uma avaliação precisa e abrangente do estado nutricional, melhorando a acurácia do diagnóstico.

## QUADRO RESUMO

A tabela 4 apresenta sugestões de condutas sobre triagem nutricional e avaliação nutricional para pacientes adultos onco-hematológico que serão submetidos ao TCTH em diversas fases do tratamento.

**Tabela 4:** Resumo da triagem e da avaliação nutricional do paciente adulto onco-hematológico em transplante de células-tronco hematopoéticas (TCTH) em diversas fase do tratamento

Fase	Avaliação
Ambulatorial	Todos os pacientes, independente do tipo de TCTH, devem ser avaliados Frequência: com risco nutricional até 15 dias e sem risco nutricional em até 30 dias NRS-2002, ASG-PPP ou ASG Perda de peso não intencional
Admissão para transplante	Triagem nutricional em até 48 horas NRS-2002, ASG-PPP ou ASG Dinamometria Anamnese alimentar Os pacientes deverão ser avaliados semanalmente, durante todo o período de internação
Início do condicionamento	Ingestão alimentar <75% das necessidades nutricionais nas últimas 2 semanas Percentagem de perda de peso
Dia do transplante	Sintomas do trato gastrintestinal de impacto nutricional por mais de 3 dias ou alternados na última semana Percentagem de perda de peso Dinamometria
Pega da medula	Sintomas do trato gastrintestinal, como esofagite, mucosite, diarreia, disgeusia e xerostomia Percentagem de perda de peso Dinamometria
1 mês após o TCTH	Sintomas do trato gastrintestinal de impacto nutricional ASG-PPP Percentagem de perda de peso Dinamometria
3 meses, 6 meses e 1 ano após o TCTH Ambulatorial	Sintomas do trato gastrintestinal de impacto nutricional ASG-PPP Percentagem de perda de peso Dinamometria

Fonte: Adaptada de Brasil. Ministério da Saúde. Instituto Nacional de Câncer José Alencar Gomes da Silva (INCA). Consenso Nacional de Nutrição Oncológica. 2ª ed [Internet]. Rio de Janeiro (RJ): INCA; 2015. 186 p [citado 2019 Maio 10]. Disponível em: http://www1.inca.gov.br/inca/Arquivos/Consenso_Nutricao_vol_II_2_ed_2016.pdf^(49)^

NRS-2002: *Nutritional Risk Screeaning;* ASG-PPP: Avaliação Subjetiva Global Produzida Pelo Paciente; ASG: Avaliação Subjetiva Global.

O TCTH é um tratamento com grande complexidade, que traz riscos nutricionais importantes. Dessa maneira, é de extrema importância que a avaliação nutricional e as intervenções nutricionais sejam realizadas adequadamente, contribuindo para a melhora dos desfechos clínicos.

## ANÁLISE DA COMPOSIÇÃO CORPORAL

A análise da composição corporal, incluindo a avaliação da massa muscular e da gordura periférica e visceral, tem demonstrado grande associação com morbimortalidade de várias doenças, como o câncer.^84-87^

No TCTH, a composição corporal tem sido estudada, apresentando importantes correlações com complicações e sobrevida.^88-90^ Alterações no estado nutricional durante o TCTH servem como indicador de prognóstico para esses pacientes.^91-93^

Embora a maioria dos pacientes não esteja desnutrida no início do TCTH, os pacientes com baixo peso e os obesos têm grande risco de morte precoce após o TCTH; associado a isso, a piora do estado nutricional durante o TCTH também é indicador de mau prognóstico para esses pacientes.^91^

Tanto a desnutrição proteico-calórica quanto a obesidade aumentam o risco de comorbidades, mortalidade, dias de internação, duração do uso de drogas imunossupressoras e desenvolvimento da DECH.^18,91,92,94,95^

A obesidade, cuja prevalência no TCTH varia de 10 a 34%,^88,89,93,96^ está associada com maior prevalência de DECH, infecções e mortalidade.^89,96^ Estudo recente, realizado em pacientes submetidos a TCTH alogênico, mostrou associação inversa entre áreas de gordura visceral e periférica com tempo livre de doença.^90^

Além disso, a redução da massa muscular, associada, entre outras coisas, ao uso de corticoides, correlaciona-se a pior prognóstico nos vários tipos de TCTH.^88^ No TCTH alogênico, essa diminuição associa-se com maior prevalência de DECH crônica e baixo desempenho.^88,94,95^

Na maioria dos estudos, a avaliação da composição corporal no TCTH tem sido realizada por tomografia computadorizada (TC), densitometria óssea de dupla absorção de raios X de corpo inteiro (DEXA) e bioimpedanciometria (BIA).^88,90,97,98^ Porém, tanto o uso da TC quanto o da DEXA, nessa avaliação, está associado à radiação ionizante e ao alto custo, não permitindo seguimento adequado dos pacientes,^90,99^ além de apresentar limitações para sua realização em pacientes obesos.^99^

No caso da BIA, embora seja um método livre de radiação ionizante, de baixo custo, que permite seguimento adequado, há limitação em seu uso para pacientes com edemas e obesos, prejudicando os resultados.^99^ No TCTH, esse é um método usado para avaliar composição corporal, devido à sua praticidade; contudo, temos limitações, como a hiper-hidratação desses pacientes, que pode superestimar a massa magra.^88,97^

A DEXA não é realizada na maioria dos serviços brasileiros, pelo alto custo e/ou ausência do método. No entanto, na pediatria, associado ao TCTH alogênico, a maioria dos trabalhos utiliza o DEXA.^100,101^ Não encontramos estudos em adultos.

A ultrassonografia (US) é um método simples, de baixo custo, prático e portátil, e surgiu como possível instrumento para avaliação da composição corporal nos pacientes hospitalizados e obesos.^99,102,103^ Esse método foi empregado em estudos clínicos e fornece a medida da espessura muscular com boa correlação com métodos padrão-ouro, como TC ou ressonância magnética (RM).^103^ A US, no idoso, permite a avaliação da sarcopenia, apresentando resultados comparáveis aos do DEXA.^106^ Além disso, é possível estudar a associação da qualidade de massa muscular com força, avaliando-se a ecogenicidade, que é baseada na medida dos *pixels* das imagens.^107-110^ Não existem estudos usando essa técnica no TCTH.

Um dos exames de rotina realizados em pacientes oncológicos, principalmente naqueles candidatos ao TCTH, é a TC do tórax, que apresenta como vantagens a precisão na quantificação de massa magra e a exata diferenciação entre o músculo e a gordura. A análise das imagens, realizada em cortes tomográficos específicos, apresenta boa correlação com a massa gorda e a massa muscular magra de todo corpo. Entre estes pontos, temos a terceira vértebra lombar e a quarta vértebra torácica.^111-114^

Além da visualização da massa magra e gorda, a TC avalia a radiodensidade, a média da atenuação da radiação em unidades de Hounsfield que, quando com baixa radiodensidade, em alguns estudos, parece ter melhor prognóstico do que sarcopenia em tumores hematológicos.^115,116^

Ainda existem poucos estudos em TCTH; contudo, um deles encontrou maior quantidade de gordura visceral e subcutânea relacionada com menor tempo de sobrevida livre de doença.^90^ Por estar na maioria dos protocolos de avaliação pré-TCTH, esse pode ser um exame promissor para avaliação de composição corporal.

A avaliação da composição corporal deveria fazer parte da avaliação nutricional rotineira dos pacientes submetidos ao TCTH. Cada serviço deveria escolher o método mais adequado e com maior custo-benefício para seus pacientes.

## AVALIAÇÃO BIOQUÍMICA

O diagnóstico do estado nutricional é essencialmente clínico. Os exames laboratoriais são utilizados de forma complementar e não devem substituir a avaliação clínica.^117^ Em pacientes submetidos ao TCTH, marcadores clássicos do estado nutricional, como a contagem de linfócitos, provas de anergia cutânea e presença de anemia carencial ficam prejudicados. Além disso, a interpretação das concentrações de proteínas séricas também se torna difícil devido ao grau de inflamação e às possíveis infecções associadas, particularmente no paciente internado.^118^ Ressalta-se a importância da coleta dos marcadores nutricionais antes do transplante, para tentar reduzir a interferência destes fatores na avaliação nutricional prévia.

## PROTEÍNAS UTILIZADAS NA AVALIAÇÃO DO ESTADO NUTRICIONAL

Na ausência de inflamação (PCR <10mg/L), valores de albumina <3,0g/dL caracterizam doente provavelmente desnutrido e com pior prognóstico. Embora a albumina não se correlacione adequadamente com desnutrição na presença de inflamação, ela ainda é forte indicador de risco para morbidade e mortalidade nestes casos. A albumina tem meia-vida de aproximadamente 20 dias, fazendo com que sua utilização como marcador imediato de melhora do estado nutricional seja limitada. A transtirretina, também denominada pré-albumina, tem meia-vida curta (2 dias), sendo sensível a quaisquer alterações de síntese proteica e catabolismo. Pode ser utilizada para acompanhamento de TNP. A proteína ligadora do retinol é a que apresenta a meia-vida mais curta (12 horas) e é bom marcador de depleção proteica.^117^

Cabe ressaltar que a avaliação das proteínas citadas deve ser feita concomitantemente com a coleta de proteínas de fase aguda. A medida da PCR visa quantificar a inflamação, auxiliando na interpretação dos exames aqui mencionados.^117^

## AVALIAÇÃO INDIRETA DO CATABOLISMO E STATUS MUSCULAR

A creatinina é formada a partir da creatina, um composto encontrado quase que exclusivamente no tecido muscular. Em pacientes com caquexia acentuada, a creatinina pode ser um marcador de perda de massa muscular. Nestes pacientes, o aumento da creatinina sérica pode significar perda de função renal, mesmo com valores de creatinina dentro da normalidade. O índice creatinina-altura (ICA), calculado a partir da creatininúria do paciente, pode ser utilizado para estimar a massa muscular, sendo que valores >60% sugerem depleção muscular grave:^117^

ICA = (creatinúria de 24 horas / creatinúria de 24 horas estimada por peso ideal) × 100

Os valores de referência de creatininúria estimada^117^ são, para homens, 23mg/kg do peso ideal, e, para mulheres, 18mg/kg do peso ideal.

A dosagem de ureia urinária em 24 horas é utilizada para calcular o balanço nitrogenado. O balanço de nitrogênio orienta a terapia de reposição proteica e representa a diferença entre o nitrogênio administrado (oferta proteica) e o eliminado (pele, fezes e urina). Valores ≥-5g em 24 horas são considerados adequados. Embora seja instrumento interessante para avaliação da TN, possui limitações em paciente com perda de função renal e grandes perdas extrarrenais, como diarreia em pacientes com DECH intestinal.^117^

Balanço nitrogenado= NA – (NE) NA =proteínas ingeridas (g em 24 horas) / 6,25NE = UU × 0,5 + (1,2 × (UU × 0,5) + 4

Sendo:

NA: nitrogênio administrado.

NE: nitrogênio excretado.

UU: ureia urinária de 24 horas em g.

## OUTROS EXAMES

A dosagem de micronutrientes e elementos traços não é rotina na avaliação do estado nutricional. Entretanto, pacientes muito desnutridos podem apresentar complicações associadas à sua deficiência. É preciso alto grau de suspeição para estas deficiências, pois as alterações clínicas são discretas, tornando seu diagnóstico difícil, de modo que a coleta do exame deve ser direcionada pela suspeição clínica.^117^

Embora não façam parte tradicionalmente da avaliação do estado nutricional, a dosagem de eletrólitos tem papel importante em pacientes submetidos ao TCTH que recebem TNP ou estão em risco para síndrome de realimentação. Sódio, potássio, cálcio, fósforo, cloro e magnésio sofrem grande influência das drogas quimioterápicas utilizadas nestes pacientes, o que influencia na prescrição da TNP. Perda acentuada de peso ou jejum prolongado (>10 dias) são fatores de risco para síndrome de realimentação, cuja marca registrada é a hipofosfatemia. A reposição de eletrólitos nestes pacientes deve ser feita previamente em relação à alimentação.^117^

A avaliação da função hepática e a de metabolismo da glicose^119^ (de preferência por meio de hemoglobina glicada), apesar de não diretamente relacionadas ao estado nutricional, são essenciais nestes pacientes. Indivíduos submetidos ao TCTH apresentam maior risco de síndrome metabólica e diabetes, e a presença de hiperglicemia tem implicação direta na prescrição nutricional e no risco de infecção após o transplante. A hepatotoxidade das drogas utilizadas, o uso de TNP e o ganho de peso pós-tratamento, além de alterações prévias da função hepática, são importantes no seguimento destes pacientes.

## EXAME FÍSICO COM ÊNFASE NO ESTADO NUTRICIONAL

Doenças hematológicas estão frequentemente associadas a alterações do estado nutricional. Em especial nos pacientes submetidos ao TCTH, a realização do exame físico com ênfase no estado nutricional é essencial durante o acompanhamento de todo processo (fases pré-transplante, pós-transplante imediato e tardio). Embora a história e o exame físico do paciente sejam considerados os pilares para um diagnóstico clínico adequado, este aspecto é, por vezes, negligenciado na prática diária.^120^ A adequada realização do exame físico, pela avaliação objetiva, é capaz de detectar desnutrição ou risco nutricional^121^ e auxiliar na definição do melhor tipo de suporte nutricional para cada paciente. Ressalta-se que, quando se fala em desnutrição, o termo inclui tanto obesidade quanto desnutrição proteico-calórica − ambas as situações associadas a um aumento de risco em pacientes submetidos ao TCTH.^119^

O exame físico é um método eficiente, de baixo custo e de realização relativamente simples.^122^ Ele deve ser feito da cabeça aos pés. Para se obter o exame físico completo, é preciso utilizar as técnicas de inspeção, palpação, percussão e ausculta. Na inspeção, deve-se observar o paciente de forma global, verificando a simetria muscular esquelética,^120,121^ lembrando-se de questionar o paciente ou seus familiares sobre mudanças aparentes na condição física e alterações funcionais observadas nos últimos meses ou após o início do tratamento.^121^ Em pacientes que apresentam mucosite, por exemplo, a inspeção da cavidade oral pode ser determinante no tipo de suporte nutricional. Suas manifestações clínicas vão desde eritema de tecidos moles até a presença de pseudomembranas e lesões erosivas, podendo levar o paciente à infecção secundária e à sepse. Higiene oral pobre, doença periodontal e cáries são fatores de risco para toxicidade bucal de quimioterápicos.^123^

As técnicas adequadas para a realização do exame físico conforme as áreas examinadas que possibilitarão a classificação do paciente em bem nutrido, perda moderada e perda severa de massa magra e de gordura subcutânea estão descritas nas tabelas 5 e 6. O avaliador deve observar sinais de perda ponderal recente, retenção de fluidos, perda de massa magra e massa gorda, e sinais específicos de deficiências de macro e micronutrientes.^124^

**Tabela 5:** Avaliação de massa magra e classificação de seu estado nutricional conforme exame físico

Áreas examinadas	Técnica adequada	Bem nutrido	Perda moderada	Perda severa
Região músculo temporal	Em frente ao paciente, observe a região, depois vire o rosto do paciente e observe a região lateral	Capaz de visualizar e sentir o músculo bem definido Ligeira protuberância ou plana	Ligeira depressão	Esvaziamento, depressão profunda
Região clavicular, músculo peitoral maior, deltoide e trapézio	Paciente ereto, observe a região frontal e dorsal Observe ossos proeminentes	Homens: ossos não estão visíveis Mulheres: ossos visíveis, mas não protuberantes	Homens: ossos visíveis Mulheres: ossos visíveis com leve protuberância	Ossos proeminentes
Ombros, região acromial e músculo deltoide	Paciente pode estar sentado ou em pé com os braços paralelos ao corpo	Arredondado, com curvas nos ombros e pescoço	Processo acromial pode se sobressair ligeiramente. Ombros em desenvolvimento de alguns ângulos	Ombro quadrado, presença de ângulos. Ossos proeminentes, acrômio com protusão muito proeminente
Região escapular, trapézio dorsal e região lombar	Paciente pode estar sentado ou em pé, mãos estendidas em linha reta contra um objeto sólido	Ossos não proeminentes, sem depressões significantes	Leves depressões, os ossos podem aparecer levemente.	Ossos proeminentes com ângulos definidos, depressões facilmente definidas entre os ombros, costelas, escapula e espinha vertebral
Dorso das mãos, músculos interósseos	Observe o músculo adutor do polegar, solicite que o paciente encoste a ponta do dedo indicador com a ponta do polegar	Protuberância muscular, pode ser plana em alguns adultos nutridos	Leve depressão	Depressão significativa
Região anterior da coxa, quadríceps	Paciente deve estar sentado com a perna apoiada na cama/cadeira, formando ângulo de 90º	Músculo desenvolvido	Depressão leve na parte interna da coxa	Depressão clara na linha da coxa
Região patelar, quadríceps	Paciente deve estar sentado com a perna apoiada na cama/cadeira, formando ângulo de 90º	Músculo protuberante, dificuldade de visualizar os ossos	Ossos com leve proeminência, menor definição muscular em torno da patela	Ossos proeminentes, pequeno sinal de músculo em torno da patela
Região posterior da panturrilha	Paciente deve estar sentado com a perna apoiada e o joelho dobrado ou sentado com a perna pendurada para fora da cama	Musculatura desenvolvida	Menor desenvolvimento do músculo, ainda com forma, mas com leve firmeza à palpação	Mínimo desenvolvimento muscular ou ausência de musculatura, ausência de firmeza à palpação

Fonte: Traduzido e adaptado de Fischer M, JeVenn A, Hipskind P. Evaluation of muscle and fat loss as diagnostic criteria for malnutrition. Nutr Clin Pract. 2015;30(2):239-48;^(121)^ Academy of Nutrition and Dietetics. Physical Exam – Parameters Useful in the Assessment of Nutritional Status [Internet]. Meridian ID: Academy of Nutrition and Dietetics. Malnutrition Coding in Biesemeier. Nutrition Care Manual. 2013 [cited 2017 Jan 1]. p. 1–3. Available from: http://www.idhca.org/wp-content/uploads/2018/07/SCOLLARD_NFPE-Idaho-Physical_Exam_MN.pdf^(128)^

**Tabela 6:** Avaliação de gordura subcutânea e classificação de seu estado nutricional por meio do exame físico

Áreas examinadas	Técnica adequada	Bem nutrido	Perda moderada	Perda severa
Região orbital	Em frente ao paciente, toque a região A retenção de líquidos pode mascarar a perda	Presença de gordura ligeiramente abaulada	Círculos levemente escuros e ausência de gordura	Círculos escuros e profundos
Região do braço e tríceps	Posicione o braço do paciente em um ângulo de 90º, com o indicador e o polegar, belisque a pele e a gordura entre os dedos	Amplo tecido adiposo entre as dobras da pele	Regular tecido adiposo entre as dobras da pele	Mínimo tecido adiposo entre as dobras da pele. Os dedos se tocam
Região torácica e lombar, costelas e crista ilíaca	Paciente se possível deve estar em pé, mãos estendidas em linha reta contra um objeto sólido	Tórax cheio, costelas não aparecem, crista ilíaca com leve/sem protusão	Costelas aparentes com leve depressão entre elas, crista ilíaca um pouco proeminente	Costelas bem aparentes, com depressões proeminentes, crista ilíaca bem proeminente

Fonte: Traduzido e adaptado de Fischer M, JeVenn A, Hipskind P. Evaluation of muscle and fat loss as diagnostic criteria for malnutrition. Nutr Clin Pract. 2015;30(2):239-48;^(121)^ Academy of Nutrition and Dietetics. Physical Exam – Parameters Useful in the Assessment of Nutritional Status [Internet]. Meridian ID: Academy of Nutrition and Dietetics. Malnutrition Coding in Biesemeier. Nutrition Care Manual. 2013 [cited 2017 Jan 1]. p. 1–3. Available from: http://www.idhca.org/wp-content/uploads/2018/07/SCOLLARD_NFPE-Idaho-Physical_Exam_MN.pdf^(128)^

A perda de gordura subcutânea e a perda muscular durante o tratamento são frequentemente associadas à redução da aceitação alimentar, bem como à resposta natural do organismo ao estresse causado pela doença.^121^ Todavia, em pacientes obesos ou com edema acentuado, a avaliação de perda de gordura e musculatura pode não ser tão simples, reforçando a importância do histórico de perda ponderal.^120^ A perda de gordura subcutânea é observada nas áreas em que o tecido adiposo está normalmente presente. Em especial nos pacientes inflamados, como é o caso dos pacientes submetidos ao TCTH, a perda de gordura subcutânea pode ser mais indolente, mesmo com catabolismo importante, tornando-se desproporcional à perda da massa muscular.^118^

A avaliação muscular deve contemplar volume, tônus e capacidade funcional. De forma geral, os músculos superiores são mais propensos à perda muscular relacionada ao *deficit* alimentar; já a perda muscular relacionada à inatividade está mais associada com a musculatura inferior.^120^ A avaliação da massa muscular pode ser feita por meio da palpação e do pinçamento das áreas observadas: inicia-se pela parte superior do corpo, examinando a região temporal e orbital, prossegue-se para região clavicular frontal e músculo peitoral maior, avança-se para a região do músculo deltoide e trapézio frontal/dorsal, ombros, região acromial e escapular, observando a região do braço e tríceps, seguindo-se para região torácica e lombar, atentando para as costelas e crista ilíaca, bem como para o dorso das mãos e músculos interósseos; segue-se para a parte inferior, observando a região dos quadríceps, patela e posterior da panturrilha.^121^ A medida da circunferência da panturrilha é uma alternativa simples para a avaliação de massa muscular, especialmente no paciente idoso. Valores inferiores a 31cm^3^ são considerados anormais.

A avaliação conjunta da função muscular é importante, devido à alta prevalência de sarcopenia nesta população.^125^ Destaca-se a avaliação da força de preensão palmar por meio de dinamômetro, que pode ser realizada à beira do leito e apresenta correlação com o prognóstico de pacientes oncológicos.^126^ Existem tabelas específicas com ponto de corte para sexo e idade; entretanto, valores inferiores a 30kg para homens e a 20kg para mulheres são classificados como redução de força muscular.^127^

Ganho de peso nestes pacientes, especialmente na fase pós-transplante imediata, pode estar relacionado ao acúmulo de líquidos, representado desde a presença de edema subcutâneo até anasarca. Para avaliar a presença de edema subcutâneo, devem-se observar a região sacral em pacientes acamados e a região do tornozelo em pacientes ambulatoriais.^120^ A presença de ascite, derrame pleural ou pericárdico e anasarca deve ser registrada na evolução do paciente.

## ACEITAÇÃO ALIMENTAR DURANTE O TRANSPLANTE DE CÉLULAS-TRONCO HEMATOPOÉTICAS

Alterações clínicas associadas ao TCTH levam a mudanças no consumo e nas experiências alimentares. O paladar é uma modalidade sensorial, que envolve aspectos cognitivos e emocionais profundamente marcados na identidade, em referências associadas ao prazer e a uma ampla gama de experiências ao longo da vida.^129^ O condicionamento quimioterápico para o TCTH é um procedimento que pode modificar a experiência alimentar.^130^

O cuidado com a alimentação deve ser iniciado na fase pré-transplante e permanecer durante todas as fases e modalidades do TCTH, tanto para garantir aporte de nutrientes e energia, como para melhorar o enfrentamento da doença. Restrições dietéticas diversas, particularidades culturais, religiosas e tipos de alimentação (vegetariana, omnívora, vegana, *kosher*, entre outras) precisam sem abordados para serem considerados na terapêutica no período de internação.

Na alimentação, o paciente expressa as dificuldades vivenciadas e as oscilações emocionais que ocorrem desde o diagnóstico da doença. O período de condicionamento é envolto em uma série de desafios, e o cotidiano é imerso em sintomas, como dor, náuseas e vômitos, associados ao sofrimento e à angústia, que impactam em vários aspectos da vida e da relação com a alimentação.^130^ É comum também a alimentação hospitalar ter pouca flexibilidade para contornar dificuldades, como horários de refeições rígidos e monotonia nas dietas hospitalares.^131^ O nutricionista precisa entender essas questões de maneira integrada e manejar a atenção nutricional, de modo a contornar essas dificuldades.

O manejo da alimentação deve ser esclarecido e negociado com o paciente e os cuidadores, que exercem influência direta no cuidado com a alimentação.^132^ A adesão dos pacientes melhora à medida que os cuidadores estão envolvidos e comprometidos com sua alimentação. A toxicidade característica do período de condicionamento limita a ingestão adequada de energia e nutrientes, o que merece atenção da equipe, por estar diretamente relacionada ao desfecho do tratamento.^133^

## MANEJO DIETÉTICO NO TRANSPLANTE DE CÉLULAS-TRONCO HEMATOPOÉTICAS

O manejo da alimentação tem como objetivos preservar ou recuperar o estado nutricional, o que contribui para o melhor prognóstico e reduz efeitos de toxicidade do tratamento; atenuar e contornar sintomas do trato digestório; e contribuir com a experiência de internação e tratamento. Estimar as necessidades nutricionais no início do tratamento, monitorar a ingestão alimentar, detectar dificuldades e manejá-las com prontidão são tarefas essenciais da atenção nutricional. A literatura mostra que a ingestão alimentar, durante o período de condicionamento, corresponde a aproximadamente metade da dieta prescrita.^131^ Dificuldades com a adaptação ao ambiente hospitalar, apresentação e atratividade dos pratos e horários das refeições são fatores relacionados à baixa ingestão alimentar.^131,133^

Várias complicações que ocorrem durante a internação, principalmente derivadas da toxicidade das drogas, exigem procedimentos dietéticos específicos. Sintomas como náuseas, vômitos, hiporexia, diarreias e mucosite podem ser contornados com mudanças na oferta de alimentos, manejo da temperatura e consistência da dieta, introdução de enzimas (lactase, alfa galactosidase, entre outras) nos alimentos em episódios de diarreia por deficiência enzimática, uso de suplementos alimentares e nutricionais especiais, entre outros.^94^

## O GOSTO

O gosto é um complexo processo de integração multissensorial,^134^ que permite tanto identificar nutrientes a serem consumidos como toxinas e materiais não digeríveis a serem evitados.^129,135^ O gosto permite o reconhecimento e a distinção dos sabores doce, salgado, azedo, amargo e *umami*, chamados de gostos ou sabores básicos.

Na cavidade oral, o gosto é formado pela diluição de substâncias químicas na saliva e sua respectiva identificação pelos receptores específicos nas papilas gustativas.^129^ O reconhecimento do sabor na nossa boca depende também do odor retronasal. Quando expiramos, enviamos pequenos sopros de ar do alimento para a parte de trás e superior da boca, através da passagem nasal, quando mastigamos ou engolimos.^135^

Alterações sensoriais são comuns no tratamento com TCTH e orquestradas por fatores, internos e externos, inter-relacionados, que dificultam conhecer os mecanismos envolvidos.

A disosmia, identificação prejudicada de odores, influência no consumo e no preparo dos alimentos.^136^ Tais condições podem se instalar e permanecer até por meses após o condicionamento, devido à toxidade das drogas usadas no tratamento.^47^ Alterações na percepção dos gostos e odores em pacientes recebendo quimioterapia chegam a aproximadamente 70% dos casos^47,136^ e podem levar a uma importante redução da ingestão alimentar, com consequente redução no aporte de energia. Conforme estudo, foi observado que pacientes com alteração no paladar reduzem aproximadamente 430kcal/dia em relação a pacientes que não apresentam essa alteração.^35,136^

Uma característica muito presente na rotina de enfermarias de TCTH é a queixa dos pacientes sobre o odor dos alimentos e náuseas provocadas por estes. Refeições pouco atrativas podem acentuar a hiporexia. Na tabela 7, são apontados exemplos de situações que influenciam na ingestão alimentar.

**Tabela 7:** Cuidados a serem adotados na rotina hospitalar para melhorar a experiência alimentar

Ambiente das refeições	Mobiliário adequado, cuidados com procedimentos de rotina (odor e horários de uso de produtos de limpeza, porta fechada de sanitário etc.) realizados simultaneamente à refeição; manter o ambiente arejado e limpo
Respeito às características individuais	Garantir que o paciente não receba os alimentos aos quais apresenta aversão ou restrição (recusa, religião ou outros motivos). Inclusão dos itens alimentares negociados com o paciente e solicitados na rotina
Suporte e acomodação para as refeições	Utensílios e mesa para apoiar bandeja ergonômicos, dieta assistida sempre que necessário, limpeza e organização do quarto
Odores e apresentação das refeições	Evitar alimentos de odor forte, usar alternativas como baixelas com *cloche*, com orifício para liberação de aromas, ou minimizar odores abrindo a bandeja previamente; evitar pratos com divisória e porções muito grandes; evitar embalagens pouco atrativas para suplementos e dietas, cuidados com o uso de utensílios descartáveis frágeis para manusear para a refeição
Restrições alimentares	Evitar restrições dietéticas desnecessárias (como dietas hipossódicas, restritas em proteínas e demais restrições que devem ser ponderadas e avaliadas)
Utilização de condimentos	Azeite, manteiga, ervas desidratadas, limão, entre outros, podem ser testados com o paciente e utilizados
Educação nutricional e suporte da equipe	Conscientização e explicação da importância e das características da dieta para pacientes e acompanhantes ajudam em sua adesão e resolução de dificuldades; as decisões das mudanças na dieta devem ser negociadas com os pacientes; a equipe deve partilhar as informações para evitar pluralidade de orientações

Fonte: Calleja Fernández A, Pintor de la Maza B, Vidal Casariego A, Villar Taibo R, López Gómez JJ, Cano Rodríguez I, et al. Food intake and nutritional status influence outcomes in hospitalized hematology-oncology patients. Nutr Hosp. 2015;31(6):2598-605;^(131)^ Gustafsson IB, Öström Å, Johansson J, Mossberg L. The Five Aspects Meal Model: a tool for developing meal services in restaurants. J Foodserv. 2006;17(2):84-93.^(137)^

### Sabor *umami*

Ainda pouco explorado, o sabor *umami,* derivado do aminoácido glutamato monossódico, representa alternativa para estimular a percepção do gosto.^135^ Ele ganhou destaque por ser um dos últimos gostos ou sabores a serem descobertos, sendo denominado o quinto sabor, em adição aos demais quatro sabores: doce, azedo, salgado e amargo.^138^ Por ativar receptores específicos para o glutamato e derivados, o taste-m GLU R4 (receptor metabotrópico do glutamato) parece ser um dos gostos mais preservados durante alterações gustativas características de períodos de exposição à toxicidade quimioterapia.^135^

O *umami* pode ser composto, além do glutamato, por outros sais, como o guanilato e inosinato dissódicos, naturalmente presentes em alimentos como queijos, tomate e peixe.^138^ Ele exerce influência sobre o fluxo salivar, promovendo uma diluição melhor de compostos químicos dos alimentos na cavidade oral. No Brasil, um estudo mostrou que ele é identificado mesmo em limiares baixos por crianças diagnosticadas com leucemias e linfomas em regime de condicionamento,^135^ mostrando ser alternativa que deverá receber mais atenção dos profissionais no manejo dietético. A dose em acordo com o Nível sem Efeitos Adversos Observáveis (NOAEL - *Non Observed Adversed Effect Level*) foi estipulada em 1.600mg/kg de peso para doses via oral − valores bem altos, mesmo considerando grandes consumidores.^139^ O uso de glutamato monossódico para temperar a comida pode ser alternativa a ser testada em pacientes com alterações no paladar.

### Sabores salgado, doce, azedo e amargo

Alterações na percepção dos sabores doce, salgado e azedo ou ácido são descritas em revisão envolvendo 446 sujeitos com diferentes regimes de quimioterapia.^140^ Todavia, a diversidade de métodos, resultados e protocolos de quimioterapia não permite concluir um perfil de alteração na detecção dos sabores. Estudo em pacientes de transplante alogênico mostrou que eles têm dificuldade em perceber a intensidade de concentrações altas e baixas do sabor doce, e as alterações no paladar persistem até 3 anos após o TCTH, podendo ser considerada uma mudança permanente e uma complicação tardia do TCTH.^141^ Isso influencia sobremaneira no manejo dietético e exige do profissional acompanhar essas mudanças e estabelecer alternativas alimentares para atenuar a decepção quando o paciente não reconhece o gosto do alimento. Duas são as possibilidades de abordagem dietética: recorrer a alimentos e preparações conhecidas e apreciadas, para incentivar o consumo de alimentos com referências pregressas positivas para o paciente; e investir em novas preparações e alimentos, para evitar a decepção provocada por alterações no paladar, colaborando para ampliar experiências alimentares.

## NECESSIDADES NUTRICIONAIS NO TRANSPLANTE DE CÉLULAS-TRONCO HEMATOPOÉTICAS

Após o diagnóstico nutricional, o próximo passo é o cálculo das necessidades nutricionais.^49,142^

As necessidades energéticas dos pacientes podem ser calculadas por equações preditivas ou por meio da CI. De acordo com a ASPEN, a CI é o método recomendado para determinar as necessidades calóricas em pacientes oncológicos críticos. Quando não disponível, as equações preditivas, como a de Harris Benedict, a Scholfield, entre outras, podem ser utilizadas. Outro método rápido e de grande aplicabilidade e efetividade para cálculo dos requerimentos energéticos é a fórmula simples, que utiliza calorias porkg/peso atual ou peso ajustado.^40,143^

O estado nutricional dos pacientes candidatos ao TCTH já é considerado por si só um fator de risco, visto que esses pacientes já se encontram em risco nutricional, devido à toxicidade terapêutica ou à doença de base.^49,144^

Durante o TCTH, ocorrem duas situações distintas e simultâneas: a diminuição da ingestão oral de alimentos e o aumento das necessidades metabólicas, que irão interferir no estado nutricional do paciente.^145,146^

A manutenção de um bom estado nutricional é de extrema importância em todo o processo do TCTH e, para isso, é necessária a oferta adequada de nutrientes.^145,146^

Um paciente com bom estado nutricional tem alto fator prognóstico. Assim, deve-se indicar um suporte nutricional antes do aparecimento de complicações digestivas. As mudanças nesses pacientes afetam o metabolismo de energia, proteínas e micronutrientes. O regime de condicionamento e as complicações do transplante (sepse, DECH, mucosite, diarreia, etc.) levam a um balanço nitrogenado negativo e à perda de músculo esquelético.^147^

O TCTH é um procedimento que necessita de altas doses de quimioterapia, devido à agressividade terapêutica, e requer cuidado nutricional especial, pois os pacientes têm risco aumentado de desnutrição pré e pós-transplante. O regime de condicionamento produz efeitos e mudanças no trato gastrintestinal e sistema imune, causando alterações metabólicas e nutricionais. A alteração do estado nutricional no pré-transplante é um fator prognóstico negativo para evolução do paciente.^147^

O TCTH alogênico tende a ser mais agressivo quando comparado com o TCTH autólogo; no entanto, quase todos os pacientes apresentam complicações independentemente do tipo de transplante e se beneficiam com suporte nutricional.^147^

No pós-TCTH tardio, pacientes podem apresentar vários problemas nutricionais, como desnutrição, em decorrência da ingesta oral insuficiente, ou desordens metabólicas, como diabetes, obesidade, dislipidemias e hipertensão.^119^

O aconselhamento nutricional deve ser adequado para cobrir as necessidades nutricionais dos pacientes, respeitando os hábitos e as preferências alimentares.^49^

Os requerimentos hídricos para os pacientes de TCTH são baseados na oferta de líquidos preconizados para indivíduos saudáveis, que é de 1m/kcal ou 35ml/kg/peso; contudo, devido às perdas dinâmicas e às retenções hídricas muitas vezes apresentadas, ajustes nesses cálculos podem ser necessários.^49,148^

## NECESSIDADES ENERGÉTICAS

O TCTH é uma condição de estresse altíssima, que necessita alto requerimento de energia, devido ao hipermetabolismo e ao aumento do catabolismo, à citoterapia de redução, às infecções, à falência de múltiplos órgãos e à reparação tecidual.^91^

O TCTH predispõe o paciente a complicações digestivas. A terapêutica imunossupressora agressiva pode provocar náuseas e vômitos, mucosite, alteração do paladar e esofagite, além de diarreia, uma complicação comum, que pode permanecer por várias semanas após o TCTH.^147^

Essas manifestações digestivas indesejáveis que acompanham esses pacientes determinam implicações negativas no aporte proteico-calórico e prejuízo na absorção de nutrientes, além do aumento das necessidades energéticas impostas pelo tratamento e pela internação prolongada. A consequência desta associação de fatores é a piora progressiva do estado nutricional.^149^

Segundo o Instituto Nacional de Câncer José Alencar Gomes da Silva (INCA), a determinação do valor energético deve levar em consideração o estado nutricional basal do paciente, o estresse metabólico, a idade e o peso.^49^

Apesar do gasto energético variar entre um transplante autólogo e um alogênico, devido ao intenso catabolismo, é consenso que os requerimentos energéticos podem variar de 130% até 150% do gasto energético basal.^146,147,150^

Na maioria dos centros de TCTH, os protocolos de recomendação energética são similares. Szeluga et al.,^151^ demonstraram que, para manter um balanço nitrogenado em zero, foram necessárias de 30 a 50kcal/kg peso/dia para adolescentes e adultos.^146,151^

Segundo critérios do *Fred Hutchinson Cancer Research Center*, no período pós-TCTH imediato (30 a 50 dias), as necessidades energéticas refletem o aumento do requerimento, devido a condicionamento, febre, infecções, DECH e outras complicações metabólicas.^146,152^

Pacientes críticos normalmente encontram-se em estado hipermetabólico e, consequentemente, apresentam requerimentos nutricionais aumentados.^143,153^

Pacientes submetidos ao TCTH apresentam risco de desnutrição devido à quimioterapia e/ou radioterapia prévias, que determinam manifestações digestivas indesejáveis, aumento das necessidades energéticas e internação prolongada.^149^

Com relação ao metabolismo de carboidratos, relata-se intolerância à glicose, causada pela administração de ciclosporina e corticoides, ou devido à sepse. As anormalidades no metabolismo lipídico são menos frequentes na primeira fase do transplante, porém são observados altos níveis de colesterol e triglicerídios no pós-transplante tardio. Os lipídeos a serem oferecidos na dieta devem conter triglicerídios de cadeia longa e média.^147^

No pós-TCTH, a hiperglicemia e as alterações lipídicas são mais comuns, aumentando o risco de comorbidades. A glicose deve ser administrada em uma proporção de menos de 5g/kg peso/dia, e os lipídios devem ser usados para completar o requerimento calórico (20% a 30%). Por essa razão, é importante fornecer doses adequadas de lipídios, a fim de reduzir a proporção de glicose em pacientes com hiperglicemia.^119^

O controle de glicose é importante durante o período pós-TCTH, pois a hiperglicemia aumenta o risco de doenças infecciosas, causadas por neutropenia, disfunções das células imune, elevação e proliferação de citocinas, catabolismo muscular e lipídico, além do aumento do risco de DECH nos TCTH alogênicos, e de morbidade e mortalidade.^119^

## NECESSIDADES PROTEICAS

O aumento do metabolismo decorre de alguns fatores, como febre, infecções, quimioterapia ou radioterapia, levando à destruição tecidual.^149^

As necessidades proteicas são estimadas, a fim de prover substrato para o reparo tecidual após a terapia de citorredução e minimizar a perda de massa magra.^146,152^

A maioria dos pacientes críticos apresenta necessidades proteicas proporcionalmente maiores do que as necessidades energéticas, devido ao fato de a proteína ser o macronutriente mais importante na reparação tecidual, no suporte da função imunológica e na manutenção da massa magra.^40,143^

Existe consenso quanto à necessidade proteica de ambos os transplantes (autólogo ou alogênico), que deve variar de 1,4 a 1,5g/kg de peso/dia, podendo chegar até a 2,0g/kg/dia) para atender às demandas nutricionais exigidas pelo transplante.^149,154^

Na tabela 8 são apresentadas as recomendações nutricionais para os pacientes adultos submetidos ao TCTH.

**Tabela 8:** Recomendações nutricionais para o paciente adulto submetido ao transplante de células-tronco hematopoéticas (TCTH)

Recomendações	Pré-TCTH	Pós-TCTH
Energia	35-50kcal/kg/dia	30-50kcal/kg /dia
Proteína	1,5-2,0g/kg/dia	1,5-2,0g/kg/dia
Glicose	Sem recomendação	Até 5g/kg/dia
Lipídios	Retirada de ácidos graxos trans da alimentação Adequação do consumo de ácidos graxos: - Saturados: <7-10% do VET, de acordo com o risco cardiovascular - Monoinsaturados: 15% do VET - Poli-insaturados: 5-10% do VET	Retirada de ácidos graxos trans da alimentação Adequação do consumo de ácidos graxos: - Saturados: <7-10% do VET, de acordo com o risco cardiovascular - Monoinsaturados: 15% do VET - Poli-insaturados 5-10% do VET
Água	35mL/kg/dia	35mL/kg/dia

Fonte: Brasil. Ministério da Saúde. Instituto Nacional de Câncer José Alencar Gomes da Silva (INCA). Consenso Nacional de Nutrição Oncológica. 2ª ed [Internet]. Rio de Janeiro (RJ): INCA; 2015. 186 p [citado 2019 Maio 10]. Disponível em: http://www1.inca.gov.br/inca/Arquivos/Consenso_Nutricao_vol_II_2_ed_2016.pdf;^(49)^ Fuji S, Einsele H, Savani BN, Kapp M. Systematic Nutritional Support in Allogeneic Hematopoietic Stem Cell Transplant Recipients. Biol Blood Marrow Transplant. 2015;21(10):1707-13;^(119)^ Franceschini SC, Priore SE, Euclydes MP. Necessidades e recomendações de nutrientes. In: Cuppari L. Guias de Medicina Ambulatorial e Hospitalar - Nutrição - Nutrição Clínica no Adulto. Barueri (SP): Manole; 2005. p. 3-32.;^(148)^ Sociedade Brasileira de Nutrição Parenteral e Enteral. Associação Brasileira de Nutrologia. Projeto Diretrizes - Associação Médica Brasileira e Conselho Federal de Medicina [Internet]. São Paulo: 2011 [citado 2019 Abr 26]. Disponível em: http://www.saudedireta.com.br/docsupload/1331171426terapia_nutricional_no_transplante_de_celula_hematopoietica.pdf;^(149)^ Tvedt TH, Reikvam H, Bruserud Ø. Nutrition in Allogeneic Stem Cell Transplantion--Clinical Guidelines and Immunobiological Aspects. Curr Pharm Biotechnol. 2016;17(1):92-104. Review.;^(154)^ Atualização da diretriz brasileira de dislipidemias e prevenção da aterosclerose – 2017. Arq Bras Cardiol. 2017;109(2 Supl 1):1-76.^(155)^

VET: Valor energético total.

## MICRONUTRIENTES NO TRANSPLANTE DE CÉLULAS-TRONCO HEMATOPOÉTICAS

Existem poucos estudos sobre o uso de micronutrientes no TCTH, e mesmo o nível de recomendação para a suplementação é baixo.^94^ Não será possível abordarmos todos os micronutrientes envolvidos nesse processo, de modo que discutiremos os mais citados na literatura: zinco, magnésio, vitaminas D e B12.

### Zinco

O zinco é um microelemento muito importante para o organismo, sendo cofator de >300 enzimas, responsável pela síntese dos ácidos nucleicos, componente estrutural de diversas proteínas, prevenção na formação de radicais livres, manutenção do sistema imunológico, entre outras funções.^156^

A deficiência ou os baixos níveis séricos de zinco têm sido observados em pacientes com leucemia. Essa alteração já foi muito estudada e caracterizada em crianças de todo o mundo, incluindo as brasileiras.^159,160^ A deficiência de zinco em crianças com leucemia mostra-se relevante a ponto de alguns estudos proporem sua suplementação como tratamento adjuvante.^161^

Nos idosos submetidos ao TCTH, a redução dos níveis séricos de zinco, já comprovada pelos estudos, parece estar relacionada com maior suscetibilidade a infecções, redução do paladar, alterações do sistema gastrintestinais, entre outros motivos.^156,162^

As manifestações clínicas da deficiência de zinco podem ser causadas por alterações genéticas (acrodermatite enteropática), uso de TNP sem suplementação de zinco, abuso de álcool, drogas (penicilamina, tiazídicos e glucagon), ingestão de cereais com alto teor proteico e ricos em fitatos, síndrome de má absorção, anemia falciforme, perdas sanguíneas e suor excessivo (em países tropicais).^156,157^ Especificamente no TCTH, a deficiência de zinco está associada à baixa ingestão alimentar, ao uso de nutrição parenteral total (NPT), à má absorção e ao aumento das necessidades para a reestruturação da medula óssea.^163^

Os sintomas de deficiência de zinco, como alopecia, diarreia, *rash* cutâneo, falha de crescimento, dermatite pustular-bolhosa, perda de peso, infecções recorrentes, dificuldade de cicatrização, redução de massa magra, hipogeusia, disgeusia, redução da acuidade visual noturna, entre outros,^156,157,164^ podem ser confundidos com os sintomas do pós-TCTH.^165^

No TCTH, a deficiência de zinco tem mostrado correlação com mortalidade tardia. Durante a nova hematopoiese e as condições inflamatórias, a necessidade de zinco está aumentada. Desse modo, manter os níveis séricos dentro da normalidade durante o processo do TCTH parece interferir no prognóstico da doença.^163^

Durante o TCTH, níveis altos de ferro sérico podem ser fator de mal prognóstico, devido ao seu acúmulo hepático. Para correção dos níveis alterados, são usados quelantes de ferro, que podem apresentar alguns efeitos colaterais. O zinco é um potente quelante do ferro, e sua suplementação e a consequentemente manutenção dos níveis séricos mais elevados, associados aos quelantes administrados a esses pacientes, parecem melhorar os resultados, reduzindo os efeitos colaterais e reduzindo, ainda, o acúmulo de ferro hepático.^166,167^

Além disso, as complicações associadas ao TCTH são mais frequentes nos pacientes com níveis séricos de zinco mais baixos, como DECH gastrintestinal agudo, episódios febris de maior duração e maior suscetibilidade a infecções. Assim, sugere-se que baixos níveis séricos de zinco possam ser fator de risco para eventos adversos durante e após o TCTH.^163,165^

Embora, essa deficiência na prática clínica seja subestimada, alguns estudos mostram 67% de deficiência em crianças, principalmente nos mais jovens, pós-TCTH. Em adultos, por sua vez, essa deficiência parece ser mais prevalente no período do pré-TCTH.^165^

No período de pós-TCTH, a suplementação de zinco tem sido sugerida como potencial tratamento, auxiliando os imunossupressores, mas sem apresentar efeitos colaterais.^168^ Todavia, não é realizada com rotina nos centros de TCTH.

### Magnésio

O uso de imunossupressores pode ocasionar a hipomagnesemia nos pacientes submetidos ao TCTH.^94^ Porém, há relatos de hipermagnesemia nesses pacientes, podendo estar associada a uso de laxante, disfunção renal ou alteração de motilidade intestinal.^169,170^

A suplementação de magnésio deve ser realizada apenas quando ocorrer diagnóstico laboratorial, devido à possibilidade de aumento ou de redução desse micronutriente.

### Vitamina D

A vitamina D pode diminuir a incidência da DECH crônica e a mortalidade no TCTH. A morbimortalidade no TCTH é atribuída à infecção, à toxicidade do sistema orgânico, à DECH e às doenças recorrentes.^166^

A vitamina D pode ser obtida por meio de duas formas no organismo humano: pela síntese cutânea endógena e por fontes alimentares. A produção endógena depende de raios ultravioleta tipo B (UVB).^167,168^ Na dieta, a vitamina D é encontrada em produtos naturais não fortificados, como peixes gordurosos (salmão, sardinha, fígado frio e óleo) ou alguns tipos de cogumelos (*shitake*), uma vez que eles têm quantidades relevantes de uma das duas formas principais, colecalciferol ou ergocalciferol.^168^

Na pele humana, o colecalciferol é sintetizado a partir do 7-dihidrocolesterol quando exposto à luz solar (UVB 290-315). O colecalciferol é biologicamente inativo e liga-se imediatamente às proteínas de ligação da vitamina D ou à albumina. Assim, a vitamina D3 é metabolizada no fígado para 25-hidroxivitamina D3, catalisada pelas enzimas CYP2R1 e CYP27A1. No rim, a 25-hidroxivitamina D3 é metabolizada em 1,25-dihidroxivitamina D3 pela enzima 1-a-hidrolase (CYP27B1), que está sob o controle estrito do hormônio da paratiroide e do fator 23 de crescimento de fibroblastos do hormônio fosfatérica (FGF-23).^167,168^

A deficiência de vitamina D pode causar osteomalacia, dor óssea, fraqueza muscular, fadiga, aumento do risco de fratura, e precipitar ou exacerbar osteopenia e osteoporose. Pode ser causada por síntese reduzida da pele, absorção reduzida no trato gastrintestinal e doenças hereditárias ou adquiridas do metabolismo. Além disso, insuficiência hepática e doença renal crônica podem causar diminuição da síntese de vitamina D; o uso de anticonvulsivantes, glucorticoides e medicamentos antirrejeição pode aumentar o catabolismo de vitamina D.^171^

A principal causa de deficiência de vitamina D em adultos é a exposição inadequada à luz solar. Pessoas com tom de pele naturalmente escuro têm proteção solar natural e precisam de exposição solar de pelo menos três a cinco vezes mais para fazer a mesma quantidade de vitamina D do que pessoas com pele branca. Além disso, obesidade, síndromes de má absorção, cirurgias bariátricas e gastrintestinais, medicamentos, distúrbios crônicos de formação de granuloma, linfomas e hiperparatireoidismo primário têm alto risco para essa deficiência.^172^

A vitamina D apresenta diversas ações no organismo. Uma delas é sua importante atuação no metabolismo mineral e na saúde óssea. A vitamina D aumenta a absorção do cálcio intestinal e do fosfato, estimula a diferenciação de osteoclastos, bem como a reabsorção do cálcio, e promove a mineralização da matriz óssea.

A vitamina D também pode estar associada com o desenvolvimento de algumas doenças, como as cardiovasculares, câncer, doenças autoimunes, *diabetes mellitus* tipo 1, esclerose múltipla e doença inflamatória intestinal.^168,173-176^

Outras importantes ações da vitamina D são a regulação da resposta imune do hospedeiro e a prevenção da autoimunidade. A vitamina D inibe a maturação das células dendríticas, polariza as populações de células T para a expressão de Th2 em comparação com as citocinas Th1 e bloqueia a proliferação de células T alogênicas. O receptor da vitamina D é expresso em vários precursores hematopoiéticos, bem como monócitos, alguns timócitos e linfócitos B e T ativos. Sua ação é potencializada pela ligação do receptor de ácido retinoico/receptor retinoide X.^168,173-176^

Na doença hematológica, a vitamina D também pode apresentar papel importante na terapia antineoplásica. Análogos da vitamina D podem participar na maturação e diferenciação de diferentes linhas celulares em uma célula sanguínea madura, atuando principalmente na SMD e LMA. Outra importante ação da vitamina D pode estar associada com a resposta imune modulada no TCTH, de modo que o receptor de vitamina D pode impactar na reconstituição imune após o TCTH e diminuir os riscos de infecção, DECH e efeitos colaterais.^168,173-176^

Os pacientes submetidos ao TCTH podem ter baixo nível de 25-hidroxivitamina D devido à diminuição da exposição à luz solar, a principal causa de deficiência da vitamina D.^172^ Tal deficiência decorre de estadias prolongadas no hospital, redução da vitamina D na dieta, uso de corticosteroides, atividade ao ar livre limitada, uso de protetor solar e diminuição da ingestão oral causada pela toxicidade do tratamento gastrintestinal.^171,177^

Além disso, a DECH gastrintestinal limita a absorção de vitamina D, e alguns medicamentos recebidos durante o TCTH podem aumentar o catabolismo desta vitamina, alterando as funções hepática e renal.

### Vitamina B12

A vitamina B12 é o cofator de apenas duas enzimas: metionina sintase e L-metilmalonil-coenzima A mutase. Ela é necessária para o desenvolvimento e o início da mielinização do sistema nervoso central.^178^

Sua deficiência pode causar anemia megaloblástica, disfunção neurológica, glossite, má absorção, infertilidade e trombose.^178^

Nos pacientes submetidos ao TCTH, pode ocorrer alteração pancreática por atrofia ou insuficiência, que pode levar a uma maior perda de gordura nas fezes, além de uma hiperproliferação bacteriana.^179^ Além disso, a presença de DECH intestinal pode prejudicar a absorção de vitamina B12.^180^

No caso de ocorrer deficiência de B12 por redução de sua absorção, a suplementação oral pode não ser suficiente para sua resolução, devendo ser iniciada a suplementação parenteral.

### Complicações nutricionais

Pacientes oncológicos comumente são submetidos à terapia antineoplásica. Na fase de condicionamento do TCTH, a utilização dessas drogas está presente em altas doses. Um dos efeitos colaterais da fase de condicionamento são as complicações nutricionais geradas em decorrência da toxicidade dos quimioterápicos. Estão entre estas complicações: mucosite, xerostomia, disgeusia, disfagia, odinofagia, náuseas, vômitos, constipação intestinal, diarreia, anorexia, anemia, levando à depleção do estado nutricional do paciente e impactando a resposta do tratamento. As células do epitélio oral são as primeiras a serem atingidas pela terapia antineoplásica devido à sua alta proliferação. O grau de complexidade da cavidade oral desses pacientes depende das drogas utilizadas, da dosagem, do estado geral e da tolerância do paciente. As complicações orais impactam diretamente o estado nutricional, pois contribuem para diminuição da ingesta alimentar. Pacientes que recebem aconselhamento nutricional individualizado no pré-transplante apresentam menor índice de efeitos colaterais e melhor tolerância ao tratamento.^181^

### Principais agentes quimioterápicos

O paciente submetido ao TCTH é um candidato à desnutrição em potencial. Um dos motivos está relacionado à toxicidade dos quimioterápicos utilizados durante a fase de condicionamento, até a pega ou recuperação medular, desencadeando decréscimo da ingestão oral.^182^

Na tabela 9 são apresentados os principais efeitos tóxicos de agentes quimioterápicos utilizados no TCTH associados à nutrição.

**Tabela 9:** Efeitos tóxicos comuns a agentes quimioterápicos mais utilizados no transplante de células-tronco hematopoéticas relacionados à nutrição

Droga	Efeitos tóxicos relacionados à nutrição
Carboplatina	Leucopenia, anemia, trombocitopenia, náuseas, vômitos, nefrotoxicidade, constipação, diarreia e anorexia
Ciclofosfamida	Mielodepressão, anorexia, náuseas, vômitos, estomatite, mucosite, colite, nefrotoxicidade, cardiotoxicidade e hepatotoxicidade
Ifosfamida	Mielodepressão, náuseas, vômitos, anorexia, mucosite, nefrotoxicidade, cardiotoxicidade e hepatotoxicidade
Carmustina	Mielodepressão tardia e cumulativa, náuseas, vômitos, anorexia, estomatite e nefrotoxicidade
Etoposida	Mielodepressão, diarreia, vômitos, náuseas, anorexia, mucosite e hepatotoxicidade

Fonte: Adaptado de Ikemori EH, Oliveira T, Serralheiro IF, Shibuya E. Nutrição em Oncologia. São Paulo: Lemar; 2003.^(182)^

### Xerostomia

A xerostomia é definida como queixa subjetiva de boca seca, que pode resultar da diminuição na produção de saliva. Os constituintes principais da saliva são água, proteína e eletrólitos. Esses componentes melhoram o paladar, a fala e a deglutição, facilitando a lubrificação, a irrigação e a proteção das membranas da mucosa do aparelho digestivo superior. Funções fisiológicas da saliva fornecem atividade antimicrobiana e tampão, que fazem a proteção dos dentes. Após o desenvolvimento dos sintomas, o paciente apresenta desconfortos, como dor, ardor e dificuldade de deglutição.^182^

As recomendações nutricionais para alívio da xerostomia são ingerir pequenas quantidades de líquidos frequentemente; estimular o consumo de balas mentosas ou de limão (avaliar condição da cavidade oral; alimentos ácidos estimulam salivação); e ofertar dietas úmidas (preparações com molhos, sopas e caldos).

### Odinofagia

É causada por úlceras ou erosões no esôfago, provocadas por processos inflamatórios de origem infecciosa (herpes, candidíase e HIV) ou química (medicamentos, ácidos e cáusticos), causando dor na deglutição dos alimentos. Dor forte e/ou sensação de queimação no peito são os sintomas característicos da odinofagia.^181,183^

As recomendações nutricionais para alívio desse sintoma são antes das refeições, providenciar alívio da dor; evitar consumo de alimentos irritantes da mucosa oral (bebidas gaseificadas, alimentos ácidos e alimentos secos/duros); preferir alimentos em temperatura ambiente, e evitar extremos entre gelado e quente; modificar a consistência da dieta ofertada de acordo com o grau de comprometimento da mucosa oral (dieta branda, pastosa, cremosa e líquida); associação de TN oral, TNE ou TNP, de acordo com a gravidade e a condição clínica do paciente.

### Disgeusia

Caracterizada pela perda ou alteração do paladar, em que a percepção das papilas gustativas são alteradas, podendo ressaltar o sabor amargo que comumente é o primeiro a sofrer alteração, seguido dos sabores doce e salgado.^184^

As recomendações nutricionais nessa situação incluem: ressaltar o sabor dos alimentos com a utilização de condimentos e especiarias; consumir alimentos cítricos; preferir preparações mornas e frias, e evitar preparações muito quentes; avaliar a necessidade de reduzir a oferta de carne vermelha, bem como suplementos ricos em ferro, pois podem potencializar o desenvolvimento de gosto metálico nos alimentos devido ao uso de quimioterápicos derivados de platinum (cisplatina, carboplantina e oxalipiatina).

### Disfagia

A disfagia é uma alteração no mecanismo de deglutição decorrente de causas múltiplas, como neurológicas e/ou estruturais, traumas de cabeça e pescoço, acidente vascular encefálico, doenças neuromusculares degenerativas, câncer de cabeça e pescoço, toxicidades de algumas drogas antineoplásicas, demências e encefalopatias. A disfagia mais frequentemente reflete em problemas da cavidade oral, faringe, esôfago ou transição esofagogástrica. A disfagia ou dificuldade na deglutição pode resultar na entrada de alimento na via aérea, gerando tosse, sufocação/asfixia, problemas pulmonares e aspiração. Também pode gerar défices nutricionais e desidratação.^182,185,186^

As recomendações nutricionais em casos de diafagia são encaminhamento para avaliação da fonoaudióloga, adaptação da consistência da dieta e utilização de espessastes para ingestão de líquidos, de acordo com a conduta da fonoaudióloga.

### Diarreia

Ocorre quando há alteração no número de episódios de evacuação e na consistência das fezes, que passam a ser líquidas. A diarreia gera anormalidade no transporte de água e eletrólitos, o que pode causar desidratação. Na maioria das vezes, pacientes submetidos ao TCTH apresentam diarreia devido à toxicidade da quimioterapia ou da radioterapia na mucosa intestinal.^183^

A diarreia é uma complicação comum em pacientes que recebem altas doses de quimioterápicos e mais comum naqueles submetidos ao TCTH. O *Clostridium difficile* tem sido frequentemente identificado como causa de diarreia infecciosa em ambiente hospitalar. O uso prolongado de antibióticos pode potencializar o risco de diarreia nos pacientes submetidos ao TCTH.^187^

A presença de diarreia pode ser frequente em qualquer fase do TCTH, merecendo investigação, já que pode ter múltiplas causas, desde a descamação das células provocada pela mucosite, passando por DECH aguda e infecções causadas por enteropatógenos bacterianos ou virais.

As recomendações nutricionais, em casos de diarreia, são: controlar ingestão de lactose, sacarose e de alimentos gordurosos e com características laxativas; limitar a ingestão de alimentos ricos em fibras ou laxativos; aumentar a ingestão de líquidos, para reidratação; e usar bebidas que promovam reposição de eletrólitos, como água de coco ou isotônicos. Ainda, em virtude das acentuadas e prolongadas condições de imunossupressão previstas, é aconselhável que sejam sempre observados cuidados fundamentais de higiene durante a manipulação e o preparo de alimentos destinados ao consumo de paciente, diminuindo ao máximo o risco de complicações infecciosas de origem gastrintestinal.

### Constipação intestinal

A constipação intestinal não causa perdas nutricionais importantes, como ocorre com a diarreia, mas é um sintoma que causa desconforto considerável aos pacientes.^183^

Os opioides são substâncias derivadas do ópio, indicados para tratamentos da dor e da dispneia, quadro comum em pacientes submetidos ao TCTH. São classificados de acordo com sua natureza química, ação farmacológica e afinidade ao receptor. Os receptores de opioides são encontrados em todo sistema gastrintestinal com maiores concentrações no antro gástrico e no duodeno proximal. Seus efeitos estão relacionados aos receptores do sistema nervoso central e periférico. Na gênese da constipação intestinal, a ação dos opioides está relacionada a diversos mecanismos: aumento da absorção de fluidos, inibição das secreções gástrica, biliar e pancreática, atuação na atividade neural gastrintestinal; diminuição da atividade propulsora; e aumento da contração segmentar. Esses mecanismos resultam na diminuição da motilidade intestinal, prolongando o contato entre o conteúdo intestinal e a mucosa, favorecendo maior absorção de água e eletrólitos, e formando, consequentemente, fezes ressecadas.^188^

Para o tratamento da constipação intestinal induzida por opioide, ainda não há evidências clínicas da eficácia da intervenção nutricional exclusiva, incluindo dieta rica em fibras e aumento da ingestão hídrica (exceto em casos de desidratação) na regulação da função intestinal. Assim, o paciente e o cuidador devem ser orientados, por meio da educação nutricional, sobre os efeitos adversos provocados pelo fármaco, esclarecendo os objetivos profiláticos da alimentação laxativa e do consumo adequado dos líquidos.^188^

Recomendações nutricionais em casos de constipação intestinal: aumentar ingestão de líquidos; incluir módulo de fibras solúveis e insolúveis; incluir alimentos integrais como grãos, arroz integral, macarrão integral, farelo de trigo, farelo de aveia, farinha de linhaça e quinoa em flocos; consumir leguminosas (feijão lentilha, ervilha, grão-de-bico e soja; preferir frutas frescas (priorizar a correta higienização); aumentar a ingestão de hortaliças; e beber sucos com propriedades laxantes (mamão, laranja e ameixa).

### Náuseas e vômitos

São efeitos comuns presentes em diversas condições clínicas, principalmente nos casos de toxicidade medicamentosa, comum no uso de drogas antineoplásicas. Esses distúrbios podem levar a deficiências nutricionais, decorrentes da baixa aceitação alimentar, bem como à desidratação, a alterações eletrolíticas e ao aumento de tempo de hospitalização, ocasionando risco eminente ao paciente em neutropenia prolongada. A presença desses sintomas tem impacto negativo e bastante significativo no estado nutricional do paciente. Sua ocorrência e severidade variam de acordo com a droga, a dose e as combinações utilizadas.^189,190^

As recomendações nutricionais para alívio dos sintomas de náuseas e vômitos são: adaptação da dieta de acordo com tolerância alimentar do paciente (preparações leves); substituição dos alimentos que causam desconforto; evitar contato do paciente com o alimento durante o preparo, porque o odor da preparação pode acentuar os sintomas; fazer as refeições em decúbito elevado (sentado ou semideitado, com cabeceira da cama elevada), e mastigando bem os alimentos; evitar alimentos muito doces e/ou gordurosos; preferir alimentos gelados/frios; evitar líquidos quentes quando estiver com náuseas; hidratar, com o aumento da ingesta hídrica; manter higiene oral adequada; preferir fazer as refeições em local arejado, evitando locais quentes e abafados.

### Gengibre (*Zingiber officinale*)

O gengibre é considerado uma planta medicinal/especiaria que, dentre suas vantagens terapêuticas, possui papel importante na prevenção das náuseas (ação antiemética), em diversas situações, como nos pacientes submetidos ao tratamento quimioterápico.

A relação direta entre o consumo do extrato de gengibre com o controle/a redução de efeitos colaterais em pacientes durante o tratamento do câncer vem sendo estudada. Estudos demonstram eficácia da utilização de 1,5g de gengibre em pó divididos em três doses diárias em mulheres submetidas ao tratamento quimioterápico para câncer de mama avançado.^191^

Estudos e revisões concluem resultados benéficos na utilização de gengibre em pacientes oncológicos submetidos à terapia antineoplásica, reduzindo efeitos colaterais de náuseas e vômitos. Entretanto, são necessários mais estudos em humanos para analisar a real eficácia desse fitoterápico. Assim, ainda não é possível acordar com a utilização do gengibre na prática clínica.^192^

### Mucosite

A mucosite é um evento adverso muito comum em pacientes que recebem quimioterapia em altas doses e TCTH.

As lesões, além de muito dolorosas, são de difícil manejo clínico, e seu aparecimento pode limitar a duração e/ou potência do tratamento proposto, além de impactar sobremaneira a qualidade de vida dos pacientes e aumentar os custos advindos da terapêutica.^193^

A injúria de barreira mucosa, ou mucosite, é o dano tecidual causado pela terapia oncológica, principalmente a quimioterapia em altas doses e a radioterapia utilizadas no TCTH. Do ponto de vista clínico, duas síndromes clínicas associadas à injúria de barreira mucosa são reconhecidas: a mucosite oral, definida pela presença de lesões eritematosas e ulcerativas em cavidade oral, e a mucosite gastrintestinal, que acomete todo o trato digestório e pode se manifestar com anorexia, náuseas, vômitos e diarreia.^194^

Em 2003, um estudo com 599 pacientes demonstrou que 303 (51%) deles que receberam quimioterapia para tumores sólidos ou linfoma desenvolveram mucosite oral e/ou gastrintestinal. A mucosite oral foi encontrada em 22% de 1.236 ciclos de quimioterapia, enquanto a forma gastrintestinal, em 7% dos ciclos. Já as duas formas se manifestaram, concomitantemente, em 8% dos ciclos. Uma percentagem ainda maior (aproximadamente de 75% a 80%) dos pacientes que receberam altas doses de quimioterapia antes do TCTH desenvolveu mucosite grave. Esta foi considerada a complicação mais debilitante do transplante.^195^

Diversas análises norte-americanas demonstraram que mucosite grave aumenta a utilização de recursos hospitalares, como a utilização de opioides, antimicrobianos e NPT, prolongando a hospitalização.^194,196^

O *European Group for Blood and Marrow Transplantation* (EBMT) realizou um estudo envolvendo 25 centros em 13 países europeus, cujo objetivo primário foi verificar a ocorrência de mucosite em pacientes submetidos ao TCTH autólogo que receberam regimes de condicionamento com altas doses de melfalano ou BEAM (carmustina, etoposide, citarabina e melfalano). Foram incluídos 197 pacientes e verificou-se que 44% dos pacientes evoluíram com mucosite oral grave com mediana de aparecimento de mucosite oral ulcerativa no D+11. O maior nível de desconforto foi atingido no D+12, coincidindo com o início da enxertia neutrofílica. A duração do uso de opioides foi de 6,5 dias nos pacientes com mucosite oral grau 4, segundo classificação Organização Mundial da Saúde. A incidência de uso de NPT variou de 19% em pacientes sem mucosite oral até 59% em pacientes com mucosite oral grau 4 e 35%, considerando todos os pacientes.^193^

Estudos recentes mostram que o mecanismo fundamental que envolve a patogênese da mucosite é mais complexo do que apenas a lesão direta ao epitélio. Os mecanismos de indução de mucosite pela quimioterapia e pela radioterapia parecem ser similares.

O modelo a seguir, com cinco fases da patogênese da mucosite, é baseado nas evidências atuais:

1. Iniciação: radioterapia e/ou quimioterapia induz dano celular, resultando na morte do epitélio celular. Acredita-se que a produção e a liberação de radicais livres também exerçam papel no dano inicial da mucosite.
2. Sinalização: adicionalmente ao dano tecidual direto, radicais livres ativam mensageiros secundários, que transmitem sinais de receptores de superfície celular para o interior das células, levando à liberação de citocinas inflamatórias, e causando dano tecidual e morte celular.
3. Amplificação: liberação de fatores de necrose tumoral alfa (TNF-α) produzidos principalmente por macrófagos, causam danos às células da mucosa e também ativam vias moleculares, que amplificam tais lesões em mucosas.
4. Ulceração: há significativo infiltrado de células inflamatórias associado à ulceração da mucosa, baseado, em parte, em produtos do metabolismo da microflora que coloniza a orofaringe. Esta infecção secundária também é responsável por produção de citocinas inflamatórias.
5. Cicatrização: esta fase é caracterizada por proliferação epitelial e diferenciação celular e tecidual, restaurando a integridade do epitélio.^194^

Manifestações clínicas da mucosite incluem sinais e sintomas de um processo inflamatório, desde discreto eritema, edema e desconforto, até dor incapacitante e ulcerações. Tais alterações interferem nas atividades diárias do paciente, como falar e se alimentar. Pode haver prejuízo na nutrição adequada ou, até mesmo, impedir completamente a alimentação.^195^ Desidratação, desnutrição e infecções, e, consequentemente, redução da sobrevida global, têm sido frequentemente associadas à mucosite.^197^

Existem três principais classificações quanto à gravidade da mucosite, apresentada na tabela 10. Entretanto, a classificação mais amplamente utilizada para mucosite oral foi proposta pela OMS.

**Tabela 10:** Comparação de graus de gravidade de mucosite oral

Grau	0	1	2	3	4
OMS	Nada	Dor discreta ± eritema	Eritema, úlcera; paciente consegue deglutir sólidos	Úlceras com eritema extenso Paciente não consegue deglutir sólidos	Nenhum tipo de alimentação é possível
RTOG	Nada	Eritema de mucosa	Placas <1,5cm	Placas confluentes >1,5cm	Necrose ou ulceração profunda
WCCNR	Sem lesões Cor: rósea Sem sangramentos	Lesões: 1-4 Cor: discretamente avermelhada Sem sangramentos	Lesões: >4 Cor: moderadamente vermelho Sangramentos espontâneos	Lesões coalescentes. Cor: muito vermelha Sangramentos espontâneos	NA

Fonte: Traduzido de Niscola P, Romani C, Cupelli L, Scaramucci L, Tendas A, Dentamaro T, Amadori S, de Fabritiis P. Mucositis in patients with hematologic malignancies: na overview. Haematologica. 2007 Feb;92(2):222-31. Review.^(198)^

OMS: Organização Mundial da Saúde; RTOG: *Radiation Therapy Oncology Group*; WCCNR: *Western Consortium for Cancer Nursing Research*; NA: não se aplica.

Não há consenso sobre prevenção ou mesmo tratamento da mucosite oral. Diversas medidas têm sido utilizadas para a prevenção de mucosite grave. A boa higiene oral, os controles periódicos da saúde dentária – incluindo prevenção e tratamento de cáries e gengivite – e a ampla orientação ao paciente são medidas importantes.

A crioterapia parece ter papel na prevenção de mucosite induzida por altas doses de melfalano.^198^ Tem como objetivo promover a vasoconstrição na cavidade oral e, assim, reduzir a toxicidade com a administração do fármaco. A crioterapia tem sido descrita na literatura por ser procedimento de baixo custo, sem efeitos colaterais e que influencia no desfecho desta complicação.^199^ A recomendação é oferecer gelo 30 minutos antes do início da infusão da droga até o final da administração. Recomenda-se atenção em relação à qualidade da água para produção dos cubos de gelo.^199^ Como os pacientes ficam por horas recebendo a medicação, tendo que simultaneamente ficar com o gelo na boca, gelos preparados com suplementos nutricionais, sucos de fruta e infusões têm sido alternativa para a crioterapia e podem, ainda, representar acréscimo de energia e nutrientes.^200^

A profilaxia antibacteriana, apesar de medida adotada em receptores dos diversos tipos de transplantes, não se mostrou eficaz na redução de microrganismos presentes na superfície da mucosa oral e que têm papel importante no desenvolvimento da mucosite.^201^

Da mesma forma, o uso de clorexidina e de estimulantes de produção de saliva, como a pilocarpina, também não se mostrou eficaz na prevenção de mucosite grave.

## TRATAMENTOS

### Tratamento paliativo

Analgesia sistêmica com morfina em bomba de infusão é o tratamento de escolha para dor relacionada à mucosite oral, tanto para adultos quanto na população pediátrica. O uso de fentanil transdérmico para este fim também foi publicado, embora necessite de confirmação, no que se refere à sua eficácia.^202^

### Laserterapia de baixa Intensidade

Os primeiros estudos em laserterapia no tratamento da mucosite oral mostraram redução da inflamação e da dor, quando comparados com grupos que não realizam a laserterapia.^203^

Estudo retrospectivo brasileiro com 43 pacientes submetidos a transplante alogênico entre 2004 e 2008 comparando dois grupos – um que realizou cuidados dentários e laserterapia de baixa intensidade e outro que não realizou tais procedimentos – concluiu que a realização dos procedimentos reduz a extensão e a gravidade da mucosite oral.

Outro estudo demonstrou que o uso de laserterapia e os cuidados dentários reduzem a morbidade da mucosite oral, reduzindo, também, os custos da hospitalização.^204^

### Fator de crescimento de queratinócitos (palifermina)

Trata-se de agente mitogênico para fibroblastos, queratinócitos e células endoteliais, aumentando a espessura da mucosa, além de atenuar efeitos do TNF-α, reduzindo a inflamação característica da mucosite. Estudos clínicos demonstraram redução na incidência e na duração de mucosite oral grave (graus 3 e 4).^205^

### Terapia nutricional na mucosite

A TN em pacientes com câncer visa minimizar a perda de peso e prevenir deficiências nutricionais específicas. Em pacientes com mucosite, há ainda o impedimento da deglutição e, muitas vezes, até de manter o alimento na boca e, eventualmente, a presença de diarreia. Nesses casos, a TN que não por via oral e não exacerbe a diarreia é a de escolha.

Em graus leves de mucosite, nos quais a alimentação oral é possível, é importante a modificação na consistência da dieta. Além disso, os alimentos não irritativos de mucosa devem ser utilizados (redução de ácidos, especiarias e sal), bem como os alimentos com textura macia devem ser estimulados.

A mucosite gastrintestinal causa dano às células da cripta, reduz a produção de enzimas digestivas, e a intolerância transitória à lactose pode ser observada. Nesse sentido, dietas pobres em laticínios podem ser necessárias.

Para os pacientes que não conseguem atingir metas nutricionais, a TNE ou a TNP deve ser considerada. É possível a passagem de sonda enteral com segurança em pacientes com mucosite mais graves. A TNP pode ser indicada para pacientes impedidos de se alimentar via oral ou via enteral devido a íleo paralítico, vômitos incoercíveis ou quadros obstrutivos. Pacientes com mucosite grave, apesar de ainda assim serem candidatos à TNE, podem ter benefícios adicionais de TNP, principalmente os que necessitarem por tempo prolongado.

Suplementação com glutamina foi, durante muito tempo, instituída como parte do manejo de mucosite gastrintestinal, mas estudos recentes obtiveram resultados conflitantes sobre seu benefício.^206^

As lesões características da mucosite oral são, em sua maioria, muito dolorosas e podem comprometer a nutrição e a higiene oral dos pacientes, além de aumentar o risco de infecção local e sistêmica. Por apresentar patogênese complexa e multifatorial, o tratamento da mucosite consiste em cuidados paliativos, como controle da dor e higiene oral, além da TN, que deve ser uma escolha multidisciplinar.^196^

## ANOREXIA

A anorexia pode ocorrer pelo processo da doença, distúrbio metabólico, efeitos colaterais do tratamento ou, até mesmo, por quadro de depressão, sendo comum em pacientes oncológicos, principalmente em doenças mais avançadas, podendo estar relacionada, também, aos efeitos de náuseas e vômitos.^49,207^

A anorexia é composta por saciedade precoce e/ou perda de apetite, sendo classificada como fenômeno bipsicossocial e estando presente em cerca de 40% dos pacientes com câncer; sua etiologia é ainda bem discutida.^207,208^

Tanto os fatores hormonais quanto a ação de citocinas estão envolvidos na regulação do apetite. O neuropeptídeo Y e a grelina são hormônios orexígenos, e o primeiro tem sido encontrado em níveis reduzidos em animais com tumores. A melanocortina está atenuada na presença do câncer e tem ação anorexígena. Citocinas como TNF-α, interleucina 1 e 6, e interferon gama são liberadas pelo tumor e apresentam ação de redução da ingestão alimentar.^207,209^

A anorexia não é o único sintoma a ser considerado na perda de peso do paciente oncológico, mesmo sendo importante no processo da caquexia.^208,210^

Trata-se da causa mais comum de ingestão insuficiente após os regimes de condicionamento, sendo a saciedade precoce e o atraso do esvaziamento gástrico os fatores que mais contribuem para a diminuição da sensação de fome. As intervenções nutricionais adequadas sugerem maior fracionamento da dieta, comer devagar e consumir líquidos apenas entre as principais refeições. Outros fatores que têm impactado o apetite são as alterações do paladar e problemas de ordem emocional, como ansiedade e depressão − tão presentes entre os pacientes do transplante, principalmente devido à internação prolongada.^211^

A anorexia ocorre normalmente entre a segunda e a terceira semana do transplante, sendo o principal fato causal o regime de condicionamento.^212^ A ingestão oral é extremamente afetada durante o transplante e permanece reduzida em até 3 semanas após o tratamento.^213^

Estudo avaliou a ingestão oral de 147 pacientes submetidos ao transplante, tendo sido observada redução da ingestão alimentar de 92%, sendo a média da ingestão calórica de 3% das necessidades calóricas basais dos pacientes. A redução importante da ingestão alimentar ocorreu principalmente entre os dias 10 e 12 do regime de condicionamento.^213^

Outro estudo observou que, em 69 pacientes acompanhados no período pós-transplante, 66% apresentaram dificuldades alimentares no dia 50 após infusão da medula, sendo a anorexia um dos motivos para a redução da ingestão alimentar. A inapetência foi reduzindo com o passar dos dias pós-tratamento, mas ainda era prevalente em 16% dos pacientes no dia 200 após a infusão.^95^

Alguns estudos investigaram a prevalência da desnutrição e sua correlação com a presença de efeitos colaterais em pacientes oncológicos em tratamento adjuvante ou neoadjuvante. Recente estudo multicêntrico envolvendo 65 hospitais e 561 pacientes submetidos a tratamento adjuvante mostrou que 90,7% apresentavam perda de peso. Esse mesmo estudo constatou que 96,4% apresentavam complicações nutricionais, como anorexia (70,9%), transtornos gastrintestinais (32,6%), disgeusia (40,5%) e disfagia, entre outros.^49^

À medida que os efeitos da toxicidade provocam redução da ingestão por um período extenso, torna-se ainda mais importante a monitorização do estado nutricional do paciente submetido ao transplante.^95^

Na conduta nutricional na anorexia,^49^ conscientizar o paciente da necessidade de comer; reduzir o volume da refeição e aumentar o fracionamento; aumentar a oferta calórica e proteica nas refeições; aumentar o consumo de lanches calóricos; aumentar o consumo de alimentos que são mais aceitos; acrescentar suplemento nutricional hipercalórico e hiperproteico; e optar por líquidos mais calóricos são pontos importantes a serem observados.

## PERDA PONDERAL

O TCTH vem sendo indicado em diversas doenças hematológicas e não hematológicas de alta morbidade. Altas doses de quimioterapia citotóxica, com ou sem irradiação corporal total, resultam em hipercatabolismo, quebra da barreira da mucosa gastrintestinal e imunossupressão. Complicações gastrintestinais e infecciosas decorrentes do condicionamento são frequentes, prejudicando o estado nutricional e contribuindo para altas taxas de morbimortalidade. Além disso, para pacientes que já estão em estado catabólico, o aumento da demanda metabólica associada ao tratamento piora ainda mais o problema.^214^

Estudo analisou o impacto do peso do paciente como parte do resultado do transplante. Quando comparados pacientes com peso atual próximo ao ideal, os pacientes com baixo peso apresentaram resultados inferiores após o TCTH alogênico, provavelmente relacionados com a terapia agressiva pré-transplante. Além disso, resultados inferiores podem refletir diferenças no volume de distribuição de drogas e no impacto do peso na farmacocinética.^215^

Pacientes que serão submetidos ao TCTH apresentam maior risco para desnutrição relacionada à quimioterapia e/ou radioterapia prévias, devido aos efeitos colaterais − muitos deles relacionados ao trato gastrintestinal, levando ao aumento das necessidades energéticas e internação prolongada.

O TCTH leva a diversos efeitos colaterais no paciente, como anorexia, diarreia, mucosite e disgeusia, que prejudicam sua aceitação alimentar e, consequentemente, pioram seu estado nutricional.^95,216^ Parte significativa dos pacientes submetidos ao transplante desenvolve mucosite, apresentando dificuldade significativa em manter a ingestão calórica adequada, mais prejudicada ainda em pacientes acometidos pela DECH aguda, que afeta o trato digestivo.^217,218^

O estado nutricional é importante fator de prognóstico no paciente submetido ao TCTH. Existem três períodos importantes nos pacientes submetidos ao TCTH: período inicial, englobando a fase de condicionamento, e a fase de citorredução, causadora de grande dano tissular. O segundo período, logo após o transplante, corresponde à presença de intensa pancitopenia, seguida de reparação dos tecidos danificados. O terceiro período, no qual o implante das células transplantadas ocorre, apresenta a possibilidade do surgimento de complicações infecciosas.^219^

Em estudo restrospectivo, observou-se que, entre os pacientes com linfoma submetidos ao TCTH autólogo admitidos em desnutrição, houve maior morbimortalidade se comparados aos eutróficos.^220^

Todos os pacientes submetidos aos TCTH apresentam risco nutricional, principalmente pelos efeitos adversos causados do tratamento prévio. Além disso, metade dos pacientes submetidos ao TCTH apresenta estado nutricional alterado até 1 ano após o transplante, sendo a perda de peso e as complicações nutricionais mais presentes nos paciente submetidos ao TCTH alogênico.^221^

Outro estudo também observou perda de peso ainda no pós-transplante autólogo e alogênico. Os autores verificaram que a perda de peso se manteve até 75 dias após o transplante de 118 pacientes estudados, mesmo naqueles que não concluíram o estudo.^95^

Em levantamento realizado em instituição oncológica do Estado de São Paulo, no ano de 2016, ocorreram 123 transplantes autólogos e alogênicos, sendo observado que a média de peso no pré-condicionamento foi de 75,6kg e a média de peso no pós-condicionamento foi de 73,8kg. Do total de pacientes, 83 perderam peso durante o transplante, com média de perda de peso de 3,2kg.

Alguns estudos avaliaram o impacto do estado nutricional em pacientes submetidos ao TCTH. Em estudo realizado para a ASBMT, em que se avaliou o impacto do estado nutricional em 1.087 pacientes que foram tratados com altas doses de melfalano submetidos ao TCTH autólogo com diagnóstico de mieloma múltiplo, os pacientes foram classificados, com base no IMC, como normal, com excesso de peso, obesos ou com obesidade grave. Não houve efeito global do IMC sobre a sobrevida livre de progressão, sobrevida global, progressão da doença ou mortalidade sem recidiva.^19,222^

O estado nutricional no pré-TCTH está relacionado à evolução clínica no pós-TCTH, interferindo indiretamente no tempo de enxertia. Devido à toxicidade intensa a que esses pacientes são submetidos, o risco para desnutrição na fase pré e pós-transplante deve ser monitorado, assim como suas complicações relacionadas ao estado nutricional e a consequente diminuição da ingesta alimentar.^214^

Pacientes com diagnóstico nutricional de desnutrição e obesidade apresentam maior risco de morte no pós-TCTH imediato, assim como pacientes com depleção de massa muscular importante têm maiores complicações clínicas e tempo de hospitalização mais longo.^88^

A desnutrição presente, muitas vezes caracterizada pela perda de peso, piora o prognóstico do paciente e sua sobrevida.^92,133^ Estudo avaliou o tempo de hospitalização de pacientes de acordo com o estado nutricional. Os pacientes classificados como bem nutridos, conforme ASG-PPP, tiveram média de internação de 16,9±6,3 dias, enquanto os pacientes classificados com risco de desnutrição ou desnutrição moderada e grave apresentaram média de internação de 23,9±9,9 dias, mostrando diferença significativa entre os grupos (p=0,002).^92^

Independentemente da manutenção na ingesta alimentar e estado nutricional atual, pacientes que serão submetidos ao TCTH necessitam de acompanhamento nutricional individualizado, com a finalidade de oferecer e orientar sobre a TN adequada para cada fase do transplante, minimizando involução do estado nutricional e consequentes complicações nutricionais futuras. Pacientes submetidos ao transplante apresentam perda ponderal não intencional, independentemente do tipo de TCTH a que sejam submetidos.^223^

## DIETA ORAL

### Suplementos nutricionais

Os suplementos nutricionais representam uma das alternativas utilizadas quando os pacientes não consomem a energia e os nutrientes necessários para cobrir suas demandas nutricionais^224^ e para oferecer nutrientes com finalidade terapêutica.

Suplementos nutricionais orais são definidos como misturas que contêm nutrientes para ingestão via oral e podem ser adquiridos como formulados prontos, manipulados com alimentos *in natura* e industrializados; também podem ser adquiridos com base na combinação de ambas as formas, de modo a ofertar energia e nutrientes de forma concentrada.^47,224^ Na tabela 11 são apresentados alguns formulados disponíveis no mercado.

**Tabela 11:** Características dos suplementos nutricionais disponíveis no Brasil

Descrição das formulações	Densidade energética (Kcal/mL)	Osmolalidade (mOsm/L)	Proteína (g)/100Kcal	Características adicionais	Nomes comercias
Formulações padrão					
Padrão em pó	1,0	350-700	3,7-7,5	Sabores: baunilha, chocolate, morango, banana. Contêm sacarose e lactose	Ensure^®^, Nutren^®^ Active, Sustare^®^, Sustain^®^, Sustacal^®^, Sustenlac^®^ e Sustevit^®^
Líquida, hipercalórica, com sabor (200-250mL)	1,25-1,5	355-749	4-10	Sabores: baunilha, chocolate, morango, banana, uva, abacaxi e maçã. Contêm sacarose e lactose. Podem conter beta-hidroxi-beta-metilbutirato de cálcio	EnergyZip^®^, Nutridrink Protein^®^, Ensure Protein^®^, Ensure Plus^®^, Ensure Plus Advance^®^, Fresubin^®^ Energy Drink, Fresubin^®^ Protein Energy Drink e Fresubin^®^ Jucy Drink
Líquida, hipercalórica, com sabor e volume reduzidos (125-150mL)	1,5-2,4	385-730	3,7-10	Sabores: baunilha, chocolate, morango, *cappuccino*, lima limão, pêssego com gengibre e frutas tropicas. Podem conter sacarose e/ou lactose. Podem conter EPA	Nutridrink Compact^®^, Forticare^®^ e Fresubin^®^ Lipid Drink
Sem sabor	1,0-1,5	400	4-11	Podem conter sacarose e/ou lactose	NUTREN^®^ Sênior
Líquida ou pastosa, hipercalórica	2,0	359-720	4,4-12,5	Podem conter sacarose. Usadas em dietas com consistência pastosa.	Nutren^®^ 2.0^®^ e Fresubin^®^ 2kcal Crème
Formulações sem sacarose/lactose					
Padrão em pó	1,0	350-470	4,9-6	Baunilha	Glucerna SR^®^ e Novasource^®^ GC
Sem sabor	1,5	365-390	5-6,2	Sem sabor	Nutridrink MAX^®^ e IMMAX^®^
Líquida (200-250 mL)	1,0	334-470	4,3-6	Baunilha, morango echocolate	Glucerna SR^®^, Diamax^®^ e Diasip^®^, Novasource GC
Líquida hipercalórica	1,5	614	5	Baunilha	Glucerna 1,5kcal^®^

EPA: ácido eicosapentaenoico (ômega-3). Consulta realizada em sites dos fabricantes em junho de 2019.

O uso prolongado e sem variedade de suplementos pode piorar a adesão e reduzir a tolerância a esta estratégia. Cuidado especial deve ser tomado com formulados de alta concentração osmolar, em quadros diarreicos, e com nutrientes que podem piorar os sintomas ou funcionar como irritantes da mucosa, como as fibras, mono e dissacarídeos.^225^ Opções com maior densidade energética são interessantes, por prover aporte calórico maior, mas geralmente apresentam essa limitação. Algumas estratégias, como usar suplementos sem sabores, intercalar as opções ou mesmo misturar essas formulações aos alimentos podem melhorar a adesão,^224^ assim como recorrer a suplementos manipulados com alimentos, que oferecem maior diversidade de sabores e consistências.

### Suplementos modulares

Podem ser opção interessante para melhorar a ingestão de um nutriente de maneira isolada, como adequar a ingestão proteica.^224^ São interessantes no manejo dietético, porque podem ser usados em preparações com alimentos mais aceitos e usuais para o paciente.

### Módulo de carboidrato

Em geral, são formulados à base de maltodextrina, sacarose ou polímeros de sacarose. Encontrados em pó e utilizados para aumentar a densidade energética, sem alterar significativamente o sabor da preparação final.

### Módulo de lipídio

À base de triglicérides de cadeia média, é usado por ser gordura de melhor absorção, para oferecer ácidos graxos essenciais ou por ambos. Encontrado principalmente na fórmula líquida, também é utilizado para aumentar a densidade energética da preparação.

### Módulo de proteína

Geralmente à base de proteínas do soro do leite, podendo conter aminoácidos essenciais, aminoácidos ramificados, hidrolisado proteico, caseinato de cálcio etc. Utilizado para aumentar a densidade proteica de preparações sem alteração de textura ou volume. Encontrados principalmente em pó.

### Suplementos do aminoácidos glutamina e do beta-hidroxi-beta-metilbutirato

O beta-hidroxi-beta-metilbutirato (HMB) é um metabólito da leucina, que tem sido usado em estudos sobre sarcopenia, com resultados mais expressivos em pacientes jovens que em idosos, como alternativa para recuperação de síntese proteica.^47^ Pacientes em tratamento para TCTH, fazendo uso de antraciclinas e corticoides, que contribuem para acentuar o catabolismo proteico, podem ter a fadiga muscular intensificada.

O papel da glutamina (aminoácido condicionalmente essencial) e seus benefícios em pacientes em TCTH não estão bem estabelecidos. Tem sido indicada por via oral, para sintomas como mucosite e odinofagia associada; todavia, os estudos não trazem resultados conclusivos pela variação metodológica.^47,226^

### Suplementos probióticos

Segundo a OMS, probióticos são organismos vivos que, em quantidades adequadas, promovem a saúde e benefícios aos seus hospedeiros.^227^ Nas publicações da área, não há evidência de ação farmacológica do probiótico no hospedeiro, mas o efeito simbiótico fica evidenciado em estudos experimentais, quando se comparam parâmetros como peso dos órgãos, débito cardíaco e resposta imunológica de animais experimentais aos germe-*free*.^227^

Sabe-se que pacientes submetidos ao TCTH apresentam intensa disbiose, frequentemente associada com intensa diarreia, DECH e infecção. A alteração no percentual das bactérias benéficas intensifica-se ainda mais com o uso de antibióticos, medicamento frequentemente utilizado no paciente transplantado devido à intensa imunossupressão a que o paciente é submetido. Contudo, em relação à suplementação de probióticos em pacientes submetidos ao TCTH, poucos mas promissores trabalhos têm mostrado que a suplementação de probióticos, além de restabelecer a disbiose intestinal, é capaz de prevenir algumas complicações pós-TCTH, como a DECH.^228^ Alguns achados discutem que o suplemento de *Lactobacillus* pode prevenir a proliferação de *Enterococcus* no intestino.^228^ Efeitos adversos temidos, como a bacteremia e a translocação bacteriana, não foram encontrados no estudo de Ladas et al.,^228^ que administrou *Lactobacillus plantarum* em crianças em tratamento com TCTH.^228^

Os principais microrganismos comercializados no Brasil são: *Lactobacillus acidophilus, Lactobacillus casei shirota, Lactobacillus casei* variedade *rhamnosus, Lactobacillus casei* variedade *defensis, Lactobacillus delbrueckii* subespécie *bulgaricus, Lactobacillus paracasei, Lactococcus lactis, Bifidobacterium bifidum, Bifidobacterium lactis, Bifidobacterium longum, Bifidobacterium animalis, Enterococcus faecium* e *Streptococcus salivarius* subespécie *thermophillus*. A quantidade mínima para ser considerado probiótico é entre 10^8^ e 10^10^ Unidades Formadoras de Colônia (UFC) por porção e pode ser comercializado em cápsulas, em pó, liofilizados ou em alguns iogurtes.^229^ Não há consenso na literatura sobre qual dose usar e nem em qual momento do TCTH deve-se iniciar a suplementação; embora sua utilização pareça promissora, são necessários mais estudos clínicos para estabelecer a segurança e a eficácia no paciente submetido ao TCTH.

### Suplementos fitoterápicos

A abordagem integrativa, que concilia saberes, práticas e recursos naturais a tratamentos convencionais, tem sido bem aceita por profissionais da área da saúde, possibilitando explorar novas alternativas para aliviar sintomas, estimular o apetite e contribuir para atenuar a ansiedade.^47^

Sempre se faz necessário reforçar a orientação ao paciente e a seus familiares, para que eles procurem esclarecer à equipe sobre o uso de fitoterápicos ou quaisquer outras substâncias naturais que estiverem utilizando.^230^ Algumas infusões, ervas e compostos podem conter substâncias que interferem no metabolismo de algumas drogas, influenciando em sua ação.^230^ Por exemplo, a *Hypericum perforatum* (erva-de-são-joão), popularmente recomendada para quadros depressivos, pode interferir no metabolismo do mesilato de imatinibe (Glivec^®^).^230^ A *Camellia sinensis* (chá verde ou matcha), conhecida por características antioxidantes e termogênicas, interfere no metabolismo do bortezomibe (Velcade^®^).^230^

### Suplementos nutricionais adicionado aos alimentos

O uso de suplementos com alimentos, combinados de maneira a ofertar energia e nutrientes específicos, com diversidade de temperatura, consistência e sabores, pode ser opção para atender a demandas nutricionais no tratamento. São alternativas que envolvem preparações alimentares com diferentes apresentações e podem estimular a ingestão alimentar e demandas nutricionais em períodos de hiporexia. Podem ser formulados de acordo com a necessidade

### Dieta para pacientes com neutropenia

A condição de neutropenia é definida como a contagem de neutrófilos abaixo de 500/µL ou menor que 1.000/µL com previsão de queda para 500/µL em 2 dias. Nessas situações, algumas medidas profiláticas são adotadas para controlar riscos de infecção.^231^ Contudo, não há consenso na literatura sobre os benefícios do uso da dieta para neutropênicos. Seu uso foi instituído na década de 1980, para prevenir infecções no trato digestório e sua característica principal era a restrição de alimentos que pudessem funcionar como vetores de microrganismos patogênicos.^47,221^ Até 2006, a literatura trazia estudos com dietas que restringiam vegetais crus, frutas frescas e secas, castanhas, iogurtes, ovos e carnes malpassadas. Estudos que compararam dietas com estas restrições a dietas normais, com alimentos higienizados corretamente, mostraram que tais restrições não protegiam os pacientes neutropênicos e, em alguns estudos, o número de complicações foi maior nos que seguiam a dieta mais restrita.^232^

Segundo o consenso da ESPEN, em função das dificuldades para se compararem estudos com metodologia variada e conceitos divergentes, inclusive em relação à condição de neutropenia, que justifica essa conduta, seu emprego não tem encontrado suporte na literatura científica.^47^ As orientações vêm seguindo em outra direção: em vez de dietas restritivas, o foco tem sido a qualidade dos procedimentos de higienização, ou seja, permitir dietas mais flexíveis, tomando os cuidados usuais na aquisição, armazenagem e preparo dos alimentos.^49,232^

Sobre a sanitização de frutas e hortaliças, é seguro o uso de soluções à base de cloro, como os hipocloritos destinados a esse fim, com 200ppm de cloro ativo, o que garante ótimo poder antimicrobiano livre da ação corrosiva.^233^ Em relação aos resíduos, a quantidade ingerida é muito inferior ao tolerado, mesmo considerando a ingestão de itens crus higienizados várias vezes ao longo do dia. Após a higienização do alimento, pode-se proceder com enxague em água tratada.^233^

As recomendações sobre os cuidados adotados neste aspecto são os utilizados para a população em geral e estão na tabela 12.

**Tabela 12:** Procedimentos sugeridos em relação à alimentação e ao preparo de alimentos

Alimento	Higienização/preparo	Solução sanitizante
Frutas e hortaliças cruas	Lavagem em água corrente e remoção de sujidades aparentes	Deixar em imersão por 15 minutos: 1 colher de sopa de solução de hipoclorito para 1L de água*
Ovos e carnes (aves e peixes)	Sempre após cocção 74ºC	Atingir 74°C no centro do alimento (suco e parte interna dos alimentos devem estar claros e não rosados ou avermelhados, ovos cozidos até a gema ficar firme, e peixes opacos e desmanchar facilmente)
Leite	Usar o pasteurizado ou UHT	Até 3 dias em geladeira após aberto
Queijos	Não consumir os de mofo branco (*brie* e *camembert*) e azul (gorgonzola)	Manter o queijo em recipiente fechado na geladeira e consumir em até 5 dias após aberto (inclusive requeijões)
Iogurtes	Não consumir iogurtes com adição de probióticos	Manter sob refrigeração e consumir em até 48 horas após aberto
Sucos e mel	Consumir os pasteurizados	Até 3 dias em geladeira após aberto
Frios (presunto e *blanquet* etc.)	Preferir os embalados a vácuo	Até 3 dias em geladeira após aberto

Fonte: Adaptado de Silva Junior EA, editor. Manual de controle higiênico sanitário em serviços de alimentação. São Paulo: Varela; 2007. p. 239-66.^(233)^

* Consumo máximo diário de cloro considerado seguro: 5.100.000µcg. Média ingerida em uma refeição com vegetais sanitizados: 7.000µcg.

A água é outro item importante que merece ser discutido, considerando os aspectos relacionados a seu tratamento, formas de comercialização e distribuição. A definição de água tratada é a da rede do sistema de abastecimento filtrada ou fervida por, no mínimo, 2 minutos ou filtrada e clorada.^233^ Existem programas nacionais vinculados ao Ministério da Saúde, como o Sistema de Informação de Vigilância da Qualidade da Água para Consumo Humano (SISÁGUA) que monitoram a qualidade da água para consumo humano e devem ser consultados periodicamente pelos profissionais de saúde, para esclarecimento das recomendações.^234^ Em cidades onde não há rede de abastecimento, recomenda-se a adição uma gota de hipoclorito de sódio para cada litro de água consumida, ou ferver a água em panela limpa.^235,236^

Em relação às águas minerais envasadas, estudo mostra que contaminação por fungos pode ocorrer no processo de envase e/ou armazenagem, não representando, então, alternativa necessariamente segura.^235^

Além disso, deve-se atentar para a higienização da caixa d’água doméstica a cada 6 meses, bem como ter conhecimento da procedência, do armazenamento e do transporte de vasilhames de água. Estes são procedimentos que devem ser esclarecidos e contribuem para a orientação dos pacientes sobre a melhor opção para uso de água com qualidade química e microbiológica.^234^

Na tabela 13 são apresentadas as principais recomendações para pacientes com neutropenia.

**Tabela 13:** Recomendações gerais para pacientes com neutropenia

Aquisição dos alimentos	Manipulação dos alimentos e preparo das refeições	Refeições feitas fora de casa
Verifique o prazo de validade dos produtos	Lave as mãos antes de manipular os alimentos, antes de iniciar as refeições e após usar o banheiro*	Verifique as condições de higiene do local onde fará as refeições. Evitar locais onde a comida fica exposta e em balcões ou onde há circulação de pessoas sem proteção
Compre leite e derivados, sucos de frutas e mel pasteurizados	Separe tábuas diferentes para preparar carne de boi, peixe ou frango. Prefira tábuas de vidro	Use molhos, condimentos, sal e açúcar apenas em sachês de porções individuais
Compre frutas e hortaliças íntegras e evite frutas e vegetais danificados	Mantenha a temperatura do refrigerador abaixo de 5ºC	Observe se os funcionários que manipulam os alimentos usam tocas
Não deguste alimentos em supermercados e pontos de venda	Descongele carnes no microndas. Gire o prato pelo menos duas vezes para descongelar de maneira uniforme	Verifique se no estabelecimento o sanitário é limpo e dispõe de sabão para higiene das mãos
Evite consumir alimentos expostos em balcões	Nunca deixe alimentos perecíveis por mais de 2 horas fora da geladeira	Evite consumir bebidas com gelo em que desconhece a procedência
Siga a ordem certa das compras: primeiro os alimentos não perecíveis, depois os perecíveis congelados e, em seguida, os perecíveis que são comercializados sob refrigeração	Use somente água tratada no preparo dos alimentos e higienize a superfície antes de começar a prepará-los	
Observe se há água no chão de balcões frigoríficos, *freezers* ou refrigeradores: é um indício de que ficaram desligados por um período	Despreze as sobras após permanecerem 3 dias em geladeira	
No transporte de alimentos, evite colocá-los em local quente	Cozinhe completamente os alimentos. Atenção a porções maiores e cortes mais espessos de carne. Quando aquecer alimento preparado, verifique se atingiu a mesma temperatura na parte interna do alimento	
Verifique se o ambiente comercial é limpo e organizado	Despreze todos os alimentos que passaram do prazo de validade (considerar também a data de abertura)	

Fonte: Agência Nacional de Vigilância Sanitária (ANVISA). Guia de alimentos e vigilância sanitária [Internet]. Brasília (DF): ANVISA; 2004 [citado 2017 Mar 18]. Disponível em: http://portal.anvisa.gov.br/resultado-de-busca?p_p_id=101&p_p_lifecycle=0&p_p_state=maximized&p_p_mode=view&p_p_col_id=column-1&p_p_col_count=1&_101_struts_action=%2Fasset_publisher%2Fview_content&_101_assetEntryId=395967&_101_type=document;^(236)^

Fox N, Freifeld AG. The neutropenic diet reviewed: moving toward a safe food handling approach. Oncology (Williston Park). 2012;26(6):572-5.^(237)^

* Associado ao uso do álcool gel.

### Nutrição parenteral

A TN no TCTH tem como objetivos manter ou recuperar o estado nutricional, evitar ou reduzir as deficiências nutricionais decorrentes da quimioterapia e/ou radioterapia, minimizar as consequências do regime de condicionamento e fornecer substrato de forma adequada, para a recuperação do sistema hematopoiético e imunológico.

Os pacientes submetidos ao TCTH podem ser considerados em risco nutricional devido à redução do aporte energético, ao prejuízo na absorção de nutrientes e ao aumento das demandas metabólicas, que podem comprometer o estado nutricional desses pacientes ao longo de todo o processo.^12,216,238,239^ Dessa forma, esses pacientes estão propensos a desenvolverem uma série de distúrbios metabólicos de gravidade variável − a maioria durante o pós-transplante imediato. Dentre as principais causas, estão os efeitos adversos do regime de condicionamento, dos medicamentos imunossupressores e da TNP.^240^

Os efeitos adversos da quimioterapia e radioterapia afetam, em especial, o trato gastrintestinal e o sistema imunológico.^238^ Desse modo, além dos sintomas de náuseas e vômitos, frequentemente ocorre o aparecimento de mucosite grave, associada a intensa odinofagia, dor abdominal, diarreia e infecções.^147^ Esse conjunto de fatores afeta ainda mais a tolerância oral, colaborando para o agravamento da perda ponderal, que pode persistir por até 40 dias após a admissão para o transplante.^241^ Consequentemente, a desnutrição pode se estabelecer rapidamente no curso da internação para o TCTH.

Em relação ao tipo de TN a ser utilizada, os dados mostram menor tendência de complicações durante o uso de TNE em relação à TNP no TCTH, particularmente para as complicações infecciosas e na propensão para formas mais graves da DECH intestinal.^242^ Contudo, o estudo de pacientes que necessitaram de tratamento intensivo em unidades de terapia intensiva não revelou associação de pior desfecho no grupo que recebeu TNP.^243,244^

A TNP mostra-se mais segura e eficaz na manutenção e na recuperação do estado nutricional em relação aos pacientes submetidos à TNE, e que apresentam intolerância gastrintestinal e mucosite grave. Análises de custo-efetividade baseadas em indicadores de desfecho definitivo têm influenciado a prática clínica de retardar a introdução da TNP para pacientes eutróficos submetidos ao TCTH autólogo em comparação com os submetidos ao TCTH alogênico, já que a duração da intolerância alimentar no primeiro manifesta-se por período menor.^40,245,246^

### Indicações

A TNP deve ser iniciada na fase pré-transplante em todo paciente elegível para o tratamento de sua doença de base e que apresente perda de peso significativa com repercussão clínica. Pacientes com magreza (IMC inferior a 18,5kg/m^2^) são candidatos à TNP precoce quando for identificada redução da ingesta oral inferior a 50% nos 3 primeiros dias após a infusão do enxerto. Na maioria das vezes, observa-se que os pacientes elegíveis para o TCTH chegam com estado nutricional satisfatório e devem iniciar a TNP quando apresentarem toxicidade gastrintestinal associada à mucosite graus III ou IV, com ingestão alimentar insuficiente nos últimos 10 dias e sem perspectiva de melhora, nos quais foi descartada a opção da via enteral.^147,232^

A TNP por acesso venoso central permite a oferta adequada de líquidos e eletrólitos, além da administração de macronutrientes, vitaminas e oligoelementos.^247^ Para atingir as necessidades nutricionais, os pacientes desnutridos necessitam de prescrição progressiva da TNP, a fim de prevenir a síndrome de realimentação. A introdução da TNP deve ser realizada de modo gradual após a análise dos seguintes critérios: disponibilidade de acesso venoso central, estado nutricional ou performance status, presença de disfunções orgânicas e distúrbios metabólicos.

A transição da TNP para alimentação via oral deve ser realizada quando houver melhora dos sintomas gastrintestinais. Os alimentos devem ser introduzidos conforme a tolerância do paciente, iniciando-se com líquidos e alimentos pastosos, evoluindo até a alimentação habitual. A suspensão da NPT deve ocorrer gradativamente, diminuindo-se a oferta calórico-proteica a 50%, conforme a aceitação alimentar. A NPT pode ser suspensa quando a ingestão por via oral ultrapassar 50% das recomendações nutricionais.^46^

Apesar de a TNP ser recurso valioso no manejo dos pacientes submetidos ao TCTH, algumas circunstâncias podem limitar seu uso, como infecção do acesso venoso, distúrbios graves no metabolismo de lipídios e glicose, sobrecarga de fluidos e disfunção hepática progressiva. Os centros de transplante que desenvolveram protocolos priorizando inicialmente a intervenção via TNE demonstraram resultados favoráveis no conjunto dos pacientes.^246^

### Vias de acesso

O desenvolvimento de cateteres venosos profundos, com suas variantes semi-implantáveis e totalmente implantáveis, possibilitou maior segurança e melhor cuidado local para a execução da TNP por tempo prolongado no TCTH. De modo geral, o paciente que será submetido ao transplante autólogo recebe um cateter profundo de curta permanência, multilúmen, sendo dedicada uma das vias para eventual TNP. Já os pacientes a serem submetidos ao transplante alogênico recebem cateter venoso profundo de longa permanência, semi-implantável, que favorecerá o manuseio do paciente no período pós-transplante.

A determinação do acesso venoso utilizado para indicação da administração da NPT deve considerar dois fatores fundamentais: o tempo de utilização de TNP e a osmolaridade da solução. A tabela 14 apresenta os critérios para determinação do acesso venoso.

**Tabela 14:** Indicação de nutrição parenteral periférica e nutrição parenteral central

Nutrição parenteral periférica	Nutrição parenteral central
Baixas concentrações de nutrientes Período de terapia nutricional inferior a 2 semanas Complemento à nutrição oral e/ou enteral	Altas concentrações de nutrientes Sítio venoso central seguro Tempo prolongado de terapia nutricional
**Observação**	**Observação**
Extremidade do cateter venoso fora da veia cava Limite máximo de 900mOsm/L da solução Menor complexidade e menor custo (em relação à colocação do cateter) Novas opções de cateteres venosos por veias periféricas	Permite a administração de soluções hiperosmolares Dedicar uma das vias do cateter venoso central para a infusão da terapia nutricional parenteral Cálculo da osmolaridade da terapia nutricional parenteral: {[gAas/L×10] + [gGlic/L×5] + [mEq_(Na+K)/L×2] + [mEq_Ca/L ×1,4] + [mEq_Mg/L×1]} = Osm total

Fonte: Pittiruti M, Hamilton H, Biffi R, MacFie J, Pertkiewicz M; ESPEN. ESPEN Guidelines on Parenteral Nutrition: central venous catheters (access, care, diagnosis and therapy of complications). Clin Nutr. 2009;28(4):365-77.^(248)^

### Formulação e prescrição

A base da oferta proteica na TNP são as formulações de aminoácidos essenciais e não essenciais. A formulação padrão a 10% para adultos contém aproximadamente 4kcal/g. A oferta proteica para o paciente crítico é crucial na disponibilidade e na otimização da demanda metabólica para síntese proteica e reparação tissular, sendo o compartimento muscular a principal fonte endógena para o *turnover* proteico. As soluções de glicose ainda fornecem 4kcal/g e devem representar, em média, 70% da fonte calórica não proteica. As emulsões lipídicas são o componente mais variável da formulação, decorrente das diferentes fontes e fornecendo concentrações de 10% a 20% de triglicerídios de cadeia média, longa, rico em monoinsaturados ou da série ômega 3. Em média, a fonte lipídica a 20% fornece 2kcal/mL e representa 30% da oferta calórica da solução. As recomendações de macronutrientes são apresentadas na tabela 15.

**Tabela 15:** Prescrição recomendada de macronutrientes para pacientes adultos em terapia nutricional parenteral (peso ideal)

Macronutrientes	Pacientes críticos	Pacientes estáveis
Proteínas	1,2-2,0g/kg/dia	1,2-1,5g/kg/dia
Glicose	<3mg/kg/minuto	<5mg/kg/minuto
Lipídios	0,5-1,0g/kg/dia	0,7-1,3g/kg/dia
Calorias totais	25-30Kcal/kg/dia	30-35Kcal/kg/dia
Líquidos	Volume necessário para fornecer os macronutrientes	30-35mL/kg/dia

Fonte: Peterson S, Chen Y. Systemic approach to parenteral nutrition in the ICU. Curr Drug Saf. 2010;5(1):33-40.^(249)^

Os pacientes que apresentam quadros moderados de mucosite e diarreia devem utilizar a meta proteica definida para pacientes clinicamente estáveis, isto é, de 1,2 a 1,5g/kg/dia. Na presença de quadros graves de mucosite, ou por alterações relacionadas a infecções graves, deve-se buscar meta proteica superior. Para aproveitamento maior do aporte proteico, o valor calórico não proteico para cada grama de nitrogênio deve ser de 100:1 a 130:1. Embora as propriedades fisiológicas da glutamina tenham suscitado grande interesse em seu emprego na TNE e na TNP, não existem estudos robustos que a recomendem dentro da solução de TNP.^232^ De fato, poderia haver efeito negativo sobre o risco de recaída tardia da doença (linfomas).^250^

O controle da glicemia é de suma importância durante a TNP, pois minimiza o risco de complicações e favorece a recuperação do estado nutricional.^216^ A hiperglicemia está relacionada a um risco aumentado de infecções, distúrbios metabólicos, disfunções orgânicas e mortalidade,^251^ sendo efeito comum do uso de inibidores de calcineurina e de corticosteroides, necessitando de monitorização glicêmica e uso de insulina. Assim, é necessário desenhar um protocolo de controle glicêmico que se adeque ao tipo de TN empregada e permita a prevenção de hipoglicemia, variabilidade glicêmica e hiperglicemia significativa.^252,253^ Não existem recomendações específicas para a faixa de glicemia ideal, sendo o consenso para pacientes hospitalizados de que se situe entre 110 e 180mg/dL.

Em pacientes submetidos ao TCTH alogênico para tumores hematológicos, foram observadas menores taxas de DECH fatal naqueles em uso de fórmula parenteral contendo teor elevado de ácidos graxos de cadeia longa (80% das calorias não proteicas x carboidratos), sendo necessário observar que se trata de estratégia de nutrição cetogênica de uso limitado a curtos períodos.^254^ Por outro lado, com a exposição dos pacientes transplantados aos efeitos hiperlipemiantes das drogas imunossupressoras, há necessidade de restrição ao uso dos triglicerídios de cadeia longa na composição da TNP.^255^

Ainda hoje, existem poucos estudos referentes ao uso de lipídios da série ômega 3 durante o TCTH.^255^ A literatura reconhece que seu uso parenteral proporciona atenuação da atividade inflamatória em tempo curto (dias); mas, a dose ideal necessária, o momento de sua administração e as questões de segurança pelas alterações sobre a função plaquetária durante níveis críticos de plaquetopenia resultam na falta de recomendações para seu uso no período inicial do transplante.

Eletrólitos, minerais, vitaminas e elementos-traço devem ser ajustados às necessidades individuais. Fatores como DECH, antibióticos, estresse metabólico, drogas imunossupressoras, diarreia e vômitos podem alterar as necessidades dos micronutrientes. Dessa forma, as concentrações séricas dos eletrólitos devem ser revistas diariamente para possíveis ajustes.^216^

Com relação aos micronutrientes, não existem estudos definitivos que particularizem as recomendações para os pacientes submetidos ao TCTH, devendo-se ter em mente o contexto no qual ocorrem as principais modificações de demanda metabólica, como estresse oxidativo, disfunção hepática ou renal.^256^ Infelizmente, não dispomos de apresentações comerciais dos oligoelementos isoladamente, obrigando a interrupção da prescrição de todos os oligoelementos quando do surgimento destas disfunções orgânicas.^260-262^ A deficiência biológica de um micronutriente precede sua manifestação clínica.^263^ Têm sido relatados casos de encefalopatia de Wernicke por deficiência aguda de tiamina durante a introdução da TNP após tempo prolongado de jejum, depleção de vitamina C em decorrência do estresse oxidativo e deficiência de zinco associada à diarreia em grande volume.^256^ As recomendações diárias de eletrólitos, vitaminas e elementos-traço estão apresentadas nas tabelas 16 a 18 [Table t30002] [Table t31002], respectivamente.

**Tabela 16:** Prescrição diária recomendada de eletrólitos para pacientes adultos em terapia nutricional parenteral

Eletrólitos	Recomendações
Cálcio (gluconato)	10-15mEq
Magnésio (sulfato)	8-20mEq
Fosfato (glicerofosfato)	20-40mmol
Sódio (cloreto, acetato ou fosfato)	1-2mEq/kg
Potássio (cloreto, acetato ou fosfato)	1-2mEq/kg
Cloreto	Conforme necessário para manter o balanço acidobásico
Acetato	Conforme necessário para manter o balanço acidobásico

Fonte: Adaptado de Peterson S, Chen Y. Systemic approach to parenteral nutrition in the ICU. Curr Drug Saf. 2010;5(1):33-40.^(249)^

**Tabela 17 t30002:** Prescrição diária recomendada de vitaminas para pacientes adultos em terapia nutricional parenteral

Vitamina	Recomendações (FDA, 2000)
A	3.300UI (1mg)
D	200-600UI (5 a 15µg)
E	10UI (10mg)
B1	6mg
B2	3,6mg
B3	40mg
B5	15mg
B6	6mg
B12	5µg
C	200mg
Biotina	60µg
Ácido fólico	640µg
K	150µg

Fonte: Adaptado de Peterson S, Chen Y. Systemic approach to parenteral nutrition in the ICU. Curr Drug Saf. 2010;5(1):33-40.^(249)^

FDA: *Food and Drug Administration*.

**Tabela 18 t31002:** Prescrição diária recomendada de elementos-traço para pacientes adultos em terapia nutricional parenteral

Elementos-traço	Recomendações
Zinco	3-6,5mg
Cobre	0,3-0,5mg
Cromo	10-15µg
Manganês	60-100µg
Selênio	60-100µg
Iodo	130µg (NP prolongada)
Ferro	1,1mg (NP prolongada)

Fonte: Singer P, Berger MM, Van den Berghe G, Biolo G, Calder P, Forbes A, et al. ESPEN Guidelines on Parenteral Nutrition: intensive care. Clin Nutr. 2009;28(4):387-400.^(263)^

NP: nutrição parenteral.

No âmbito da adequação dos macro e micronutrientes na prescrição da TNP, devemos considerar a existência de dois sistemas: a TNP individualizada e a TNP industrializada, previamente descrita como pronta para uso (PPU). Embora a individualizada permita adequação mais próxima das necessidades de cada paciente, a maioria dos centros utilizada a industrializada, sem prejuízo para os pacientes.^264^

A adição destes componentes da TNP no momento de sua instalação favorece sua biodisponibilidade, mas compromete a segurança quando gera desequilíbrios físico-químicos na solução ou por aumentar o risco de infecção durante sua manipulação.^264^ Com relação aos produtos existentes no mercado, sua composição, de modo geral, distancia-se também das recomendações da tabela 15.

As soluções de TNP manipuladas seguem princípios farmacológicos e são finalizadas pelo farmacêutico, favorecendo a oferta mais adequada de nutrientes para o paciente transplantado, devendo ser seguidas normas de segurança para sua produção (adequadas terapeuticamente e no âmbito farmacêutico, livres de substâncias pirogênicas, estéreis, nas doses e composições corretas, identificadas, estocadas e administradas dentro de validade definida). Contudo, quando o cálculo das necessidades nutricionais se enquadra em uma formulação PPU, esta torna-se custo-efetiva, facilitando o processo envolvido.^264^

O emprego da CI é de grande valia para a prescrição da TNP, favorecendo o melhor entendimento sobre a demanda metabólica e a individualização das necessidades calórico-proteicas. A partir da combinação das recomendações específicas para pacientes graves com a produção em escala de novas formulações PPU, busca-se atingir sua utilização até mesmo para pacientes pediátricos.^265^

### Complicações

A TNP está relacionada a efeitos metabólicos adversos, oriundos de estimativas inadequadas das necessidades nutricionais, da magnitude do estado catabólico e por problemas com o acesso venoso central ou periférico, podendo ainda reduzir a taxa de resposta tumoral à quimioterapia. Há um consenso relativo de que é preferível adotar-se oferta calórico-proteica conservadora, da ordem de 25kcal/kg/dia e até 1,5g/kg/dia, de modo a não induzir sobrecarga metabólica ou hídrica, com alterações da função hepática ou renal. O desenvolvimento da síndrome da obstrução sinusoidal é a complicação não infecciosa mais grave da etapa inicial do TCTH, resultado da toxicidade do condicionamento tanto do TCTH autólogo quanto alogênico, e, usualmente, manifesta-se nas três primeiras semanas do tratamento. O contexto clínico de ganho de peso, hiperbilirrubinemia, seguido de oligúria e encefalopatia hepática, determina a necessidade de interrupção da TNP na forma mais grave. Outras complicações metabólicas relacionam-se com hiperglicemia, maior tempo de uso de diuréticos e retardo da recuperação do apetite. Recomenda-se a adição de insulina regular na solução de TNP, levando-se em conta relação segura de gramas de glicose por unidade de insulina (usualmente inicia-se com 20:1 a 10:1). Nas soluções de TNP por via periférica, a adição de hidrocortisona e de heparina previne as complicações inflamatórias e a retirada precoce do acesso. Caso ocorra elevação das enzimas hepáticas fora do contexto da síndrome da obstrução sinusoidal, recomenda-se alterar a infusão da TNP para ciclos de 12 horas a 20 horas, reduzir o aporte calórico não proteico (menos 10% a 15% do aporte anterior), avaliar a deficiência de micronutrientes (colina, carnitina e taurina), estimular a ingesta oral, e ajustar a dose do urso-desoxicolato para 20 a 30mg/kg/dia. Considerar o uso de metronidazol para reduzir a endotoxemia portal. Nos quadros colestáticos, deve-se limitar o uso de cobre e manganês, em função de neurotoxicidade.

Cabe à equipe de enfermagem a vigilância quanto aos procedimentos de administração da TNP, sendo requerido, por parte do profissional, o gerenciamento do tempo de infusão da TNP, adequando-o aos horários de infusão de outras drogas. É recomendável realizar o aprazamento do polivitamínico em uma primeira metade do dia, e os oligoelementos na segunda metade. Os *deficits* eletrolíticos devem ser corrigidos por meio de soluções concentradas, para minimizar a sobrecarga hídrica (caso do acesso venoso central), guardando-se os limites máximos de segurança de infusão para cada eletrólito.

O acesso venoso representa a fonte de complicação mais comum relacionada à TNP, em especial a de origem infecciosa. Outras complicações são trombose local, tromboembolismo venoso, desposicionamento, fraturas de cateter e vazamentos por dano mecânico.^12^

A TNP é um procedimento inerente ao TCTH, sendo intervenção que se insere no campo da qualidade e da segurança nos inúmeros aspectos relativos a ele. Alterações associadas à administração da TNP e privação de nutrientes por via enteral são complexas e parcialmente conhecidas, com múltiplas áreas para estudos, que levam a uma maior segurança desta terapêutica. Como perspectivas, são necessários estudos que abordem a utilização sequencial ou combinada da TNE e TNP no desfecho clínico e taxas de complicações.^47^ Especificamente no caso da glutamina e dos ácidos graxos ômega 3, são necessários trabalhos que envolvam a avaliação de seus efeitos na mucosite, diarreia, infecções, DECH e taxa de recaída.

No entanto, é importante que os estudos apresentem a separação dos pacientes por estado nutricional, tipo de tumor (sólido x hematológico) e tipo de transplante (autólogo x alogênico), a fim de diminuir os vieses de interpretação que advêm da avaliação conjunta dessas características em um mesmo trabalho.

### Nutrição enteral

A TNE consiste no conjunto de procedimentos terapêuticos para manutenção ou recuperação do estado nutricional do indivíduo, por meio da nutrição enteral, seja por via oral ou de sonda de acesso ao sistema digestório. A ASPEN e ESPEN publicaram diretrizes sobre suporte nutricional de pacientes de TCTH, recomendando TN artificial para pacientes desnutridos, ou que apresentem ingestão alimentar ou absorção intestinal diminuída durante um período prolongado.^40,47,266^

No contexto do TCTH, a melhor TN deve ser estabelecida para evitar perda de peso e alteração do estado nutricional, tendo em vista o aumento das necessidades nutricionais, as toxicidades gastrintestinais que interferem na tolerância da alimentação e os conflitos psicossociais envolvidos nesse procedimento.

Esses fatores favorecem a piora do estado nutricional, da qualidade de vida, da atividade física e da massa corporal magra por até 100 dias pós-TCTH.^63,93,267,268^ Além disso, pacientes que mantêm perda de peso por até 3 meses após o TCTH alogênico apresentam alto risco de mortalidade não associada à recorrência de doença e pior sobrevida.^269^ Torna-se claro que a intervenção nutricional deve fazer parte da terapêutica do TCTH, pois contribui para o sucesso do tratamento.

No que tange à escolha da TN, a TNP ainda é a primeira opção em muitos centros de TCTH, pela facilidade de acesso venoso central para sua administração e por ser vista pelas equipes de saúde, familiares e pacientes como menos traumática e desconfortável.^24,270^ Entretanto, a TNE é mais fisiológica e mantém o trofismo intestinal, fundamental para evitar translocação bacteriana e subsequente infecção sistêmica.^242^

Contrariando a escolha habitual da TNP, estudos demonstram que esta pode promover atrofia intestinal e alterações na imunidade gastrintestinal, em função da diminuição da celularidade do sistema GALT (tecido linfático associado ao intestino) e da perfusão vascular, reduzindo significativamente o número de linfócitos na lâmina própria. Também ocorrem alterações importantes na liberação da IgA intestinal e na produção de antimicrobianos pelas células de Paneth (conglomerados linfonodulares ileais), prejudicando a barreira epitelial e as clivagens químicas, alterando o microbioma (disbiose) e aumentando a suscetibilidade infecciosa.^271,272^

A perda da integridade da mucosa intestinal diminui a absorção de nutrientes, facilita a colonização por bactérias patogênicas e promove infecções por translocação. Além disso, a enteropatia exsudativa, a proteólise e a perda de nitrogênio, dadas em consequência desse processo, contribuem para complicações, como sepse e DECH. Essas alterações podem ser prevenidas pelo uso da NE, o que favorece sua escolha.^75,271^

Estudo realizado por Seguy et al.,^273^ demonstrou melhor sobrevida em 100 dias para pacientes que utilizaram TNE em comparação com aqueles submetidos à TNP. Em outro estudo, realizado pelos mesmos autores, o uso de TNE resultou em menores incidências de DECH, por limitar a atrofia intestinal.^43^ Em outra comparação entre as duas terapêuticas, os pacientes que fizeram uso de TNP apresentaram maior risco de infecção.^242^

Na mesma direção desses achados, diversos outros ensaios clínicos consideraram a TNE tão eficaz quanto a TNP, mas com menores taxas de complicações, além de estar associada com melhor taxa de sobrevida, menor incidência de DECH aguda e recuperação mais rápida de neutrófilos. A TNP só foi recomendada em casos de mucosite grave ou insuficiência gastrintestinal.^47,232^

Assim, a TNE deve ser estimulada durante o TCTH, contrariando preconceitos dos pacientes, dos familiares e dos profissionais envolvidos. Para tal, deve-se incentivar a implantação de protocolos de assistência nutricional^246^ que valorizem a TNE como opção viável e factível, ainda que seja necessário o desenvolvimento de habilidades para o manejo das toxicidades gastrintestinais decorrentes dessa modalidade terapêutica.

### Indicação de terapia nutricional enteral

A TNE está indicada para paciente com aceitação <60% das necessidades nutricionais por via oral, previamente ao TCTH ou tardiamente ao transplante, bem como para pacientes desnutridos com aceitação nutricional (dieta + TN oral) <60% das necessidades nutricionais previamente ao TCTH ou tardiamente ao transplante (sem presença de mucosite e plaquetopenia).^246^ Ponderar o uso de TNE, se houver indicação com recusa da TN oral pré-TCTH.^47^

Pacientes que apresentem histórico de alimentação inadequada/irregular e má aderência às orientações nutricionais observadas na avaliação pré-TCTH devem ser avaliados individualmente quanto à indicação de TNE, buscando, em conjunto com os profissionais das áreas específicas, identificar se há questões psicossociais envolvidas que interfiram no tratamento.

Pode-se considerar a indicação para pacientes em TCTH mieloablativo pela provável maior toxicidade e para idosos em uso de melfalano em altas doses em TCTH autólogo, devido ao maior risco de desenvolver mucosite grave, apesar de não ter evidências para indicação formal de TNE nessa população.^242,274^

A passagem do cateter de alimentação enteral deve ser feita precocemente, por conta das toxicidades gastrintestinais.^275^ Sugere-se a inserção do cateter de alimentação enteral até D+3, quando as toxicidades gastrintestinais frequentemente não são acentuadas.

A equipe de saúde, o paciente e seus familiares devem ser abordados, no que diz respeito à possibilidade de indicação de TNE na avaliação pré-TCTH, sanando dúvidas e mitos acerca da TN e esclarecendo seus benefícios.^30,242^ A equipe de saúde deve ser encorajada ao uso de TNE como benefício ao tratamento e ao paciente, entendendo que a TN no TCTH deve ter o enfoque de prevenir^72,275^ a deterioração do estado nutricional e os riscos envolvidos ao paciente com relação à nutrição.

Em pacientes com possibilidade de nutrição por via oral, uma vez iniciada a TNE, a alimentação via oral pode ser continuada e deve ser estimulada para desmame rápido e adequado da TNE. Há indicação de TNE em pacientes em desmame de TNP que apresentem aceitação alimentar ≥60% das necessidades por 3 dias consecutivos.^246^

### Posição do cateter nasoenteral/sonda

A posição gástrica é preferida pela melhor tolerância às fórmulas enterais, aceitando sobrecargas osmóticas e maior capacidade de armazenamento que o intestino. Caso o paciente apresente vômitos com deslocamento da sonda, a sonda deve ser alocada em posição entérica. Se houver necessidade de repassagem do cateter nasoenteral por mais de duas vezes, a TNP deve ser avaliada quanto aos riscos e benefícios dessa terapia.

O uso de gastrostomias está reservado para pacientes que apresentem desnutrição com comprometimento da ingestão oral da alimentação pela doença de base,^47^ devendo-se passar, previamente, ao transplante, como parte da terapêutica da doença.

### Formulações de dieta enteral

Sugere-se o uso de TNE industrializada pelo menor risco de contaminação e pelo melhor aporte de macro e micronutrientes para os pacientes em TCTH. A fórmula enteral indicada é aquela que atinge as necessidades nutricionais do paciente com boa tolerância. Preconiza-se o uso de TNE polimérica, hipercalórica e hiperproteica.^30,40,246^ Recomenda-se infusão contínua, com uso de bomba, para melhor controle da dieta. É fundamental que a equipe estabeleça critérios para atingir a necessidade nutricional do paciente em menor tempo possível.^276^

Como forma de complementar a nutrição via oral, Bay et al.,^30^ recomendam a infusão da TNE no período noturno, em torno de 10 horas de infusão, iniciando-se com 20 mL por hora e evolução do volume entre 10mL a 20mL por noite, tendo como objetivo administrar entre 1.000 a 1.500kcal por dia.^30^

O monitoramento da TNE deve ocorrer visando à tolerância do paciente em relação à terapia iniciada, ao equilíbrio metabólico e à detecção de toxicidade.^30,277^ Esses monitoramentos, independentemente da TN adotada, via oral, TNE ou TNP, devem ser associados à vigilância do estado nutricional, do equilíbrio de fluidos e das funções renal e hepática durante todo o procedimento do TCTH.^30^

### Contraindicações da terapia nutricional enteral

As principais contraindicações para manter ou indicar a TNE são mucosite, hemorragias digestivas, colite neutropênica, vômitos e diarreia incoercíveis (graus III e IV) e refratários à terapia medicamentosa, obstrução do trato grastrintestinal, íleo paralítico, DECH intestinal grave (grau III e IV) refratária à terapia medicamentosa e sinusopatia. Salienta-se o risco de mucosite, sem que esta já esteja instalada, não sendo contraindica a TNE.^40,47,94,246,275^

Na tabela 19 são apresentadas as principais complicações relacionadas à TNE.

**Tabela 19:** Principais complicações relacionadas à terapia nutricional enteral (TNE)

Complicações	Descrição	Medida preventiva
Mecânicas	Deslocamento ou remoção acidental do cateter nasoenteral Obstrução do cateter nasoenteral	Fixar adequadamente o cateter nasoenteral, marcando o local de saída com tinta indelével para monitorar o posicionamento Lavar sempre o cateter nasoenteral com água filtrada após a administração de dieta enteral, a cada pausa de dieta, antes e após a administração de cada medicação Verificar o posicionamento do cateter nasoenteral quando houver tosse, vômito e agutação do paciente Na presença de vômito, pausar a dieta ofertada e pausar a alimentação por 15 minutos, acompanhando posteriormente se ocorrem novos episódios de vômitos Utilizar seringa de 20mL com água morna para desobstruir o cateter nasoenteral
Gastrintestinais	Náuseas e vômitos Diarreia Distenção abdominal Constipação Desconforto abdominal Esofagite de refluxo	Manter boas práticas de preparo e conservação das dietas enterais (em caso de dúvidas, questionar o nutricionista quanto ao armazenamento prévio, à instalação e à validade do produto) Controlar o gotejamento ou o volume infundido por bomba dr infusão contínua (BIC) Manter cuidados de higiene e temperatura na administração das dietas Manter decúbito do paciente elevado (30 a 45°) durante a alimentação e 30 minutos após
Pulmonares	Colonização bacteriana Aspiração Septicemia Pneumonia Pneumotorax	Monitorar exames laboratoriais e radiológicos para avaliar a posição do cateter nasoenteral Manter o paciente em decúbito elevado antes e após a administração das dietas enterais Preferir posição jejunal do cateter nasoenteral em pacientes com risco de broncoaspiração
Otorrinolaringológicas	Lesão Necrose ou abscesso nasal Sinusite Rouquidão Otite	Utilizar cateter nasoenteral de material flexível Em TNE prolongada, substituir o cateter a cada 3 meses Manter fixação e higienização adequada do cateter e da narina do paciente Ao trocar o cateter nasoenteral, mudar o lado da inserção da narina do paciente

Fonte: August DA, Huhmann MB; American Society for Parenteral and Enteral Nutrition (A.S.P.E.N.) Board of Directors. A.S.P.E.N. clinical guidelines: nutrition support therapy during adult anticancer treatment and in hematopoietic cell transplantation. JPEN J Parenter Enteral Nutr. 2009;33(5):472-500.^(40)^ Stroud M, Duncan H, Nightingale J; British Society of Gastroenterology. Guidelines for enteral feeding in adult hospital patients. Gut. 2003;52(90007 Suppl 7):vii1-12.^(277)^ Waitzberg D, Dias MC, Isosaki M. Manual de boas práticas em terapia nutricional enteral e parenteral do HCFMUSP (Hospital das Clínicas da Faculdade de Medicida da Universidade de São Paulo). 2nd ed. São Paulo: Atheneu; 2015. 431 pp.^(278)^ Bankhead R, Boullata J, Brantley S, Corkins M, Guenter P, Krenitsky J, et al. Special Report Enteral Nutrition Practice Recommendations. 2009;33(2):122-67.^(279)^ Harris AE, Styczynski J, Bodge M, Mohty M, Savani BN, Ljungman P. Pretransplant vaccinations in allogeneic stem cell transplantation donors and recipients: an often-missed opportunity for immunoprotection? Bone Marrow Transplant. 2015;50(7):899-903. Review.^(280)^

### Toxicidades gastrintestinais

Durante o TCTH, diversas toxicidades gastrintestinais são observadas como resultado da ação de agentes quimioterápicos da terapia de condicionamento, da irradiação do corpo inteiro e dos antimicrobianos. Muitos são os esquemas de condicionamentos e variado é o comprometimento do trato gastrintestinal que pode ser graduado pela *Common Terminology Criteria for Adverse Events* (CTAE), sendo de fundamental importância seu acompanhamento durante o uso de TNE.^281^

Em linfoma não Hodgkin, a comparação das toxicidades em três esquemas de condicionamento resultou em maiores toxicidades no esquema BEAM, com comprometimento da mucosa (acima do grau II), diarreia e febre; enquanto no BEAC (carmustina, etoposide, citarabina e ciclofosfamida) os pacientes apresentaram mais anorexia, vômitos e sangramentos; e no CBV (ciclofosfamida, carmustina e etoposide), houve menores incidências de toxicidades.^282^ Em outra análise, colite neutropênica foi mais frequente em pacientes com linfoma não Hodgkin tratados com BEAM.^283^

Náusea, vômito, inapetência, diarreia e mucosite não contraindicam o uso da TNE, mas são sintomas desfavoráveis à nutrição adequada do paciente em TCTH e devem sem avaliados e acompanhados de perto para melhor adequação da TN.

A implantação de protocolos de assistência a náuseas e vômitos deve ser estimulado nos serviços. Sintoma de vômito que cause deslocamento da sonda não contraindica a TNE, mas indica a necessidade de posicionamento da sonda de alimentação enteral pós-piloro.

Casos de diarreia devem ser avaliados quanto à possibilidade de infecções como *C. difficile*, e ter sua frequência e seu volume averiguados diariamente. O manejo deve ser feito pela equipe médica e de nutrição. Se a diarreia for volumosa, sem melhora em três dias, tendo sido descartadas possibilidades infecciosas e DECH de trato gastrintestinal, o volume de dieta e a formulação devem ser revistos. É fundamental o paciente ser esclarecido a diarreia não é causada pela TNE.

Entretanto, estudo que avaliou sintomas gastrintestinais durante o TCTH autólogo e alogênico com diferentes esquemas de condicionamento verificou que a maioria dos pacientes que consumiu <60% de suas necessidades calóricas até o momento da enxertia apresentava menos diarreia. Talvez esse fato decorra da ingestão de nutrientes que contribuem para a recuperação do epitélio intestinal, impactando na melhor ingestão oral.^284^

Uma possibilidade terapêutica para a diarreia é o uso de probióticos, que têm destaque na prevenção e no tratamento da diarreia associada à antibioticoterapia.^285,286^ Entretanto, não foi estabelecida segurança nem evidência para indicá-los via TNE nos pacientes em TCTH. Apenas uma cepa apresentou segurança do uso para crianças e adolescentes durante a neutropenia do TCTH, mas o uso em adultos e idosos e as dosagens do probiótico ainda não foram estabelecidas.^228^

Quanto à escolha de formulação das dietas enterais no período de diarreia, o uso de formulações elementares ou semielementares é controverso e deve ser avaliado individualmente, tendo em vista que tais formulações possuem maior osmolaridade, e a diarreia apresentada durante o período de TCTH é, na maioria das vezes, osmótica.

Considerando as toxicidades gastrintestinais e o uso de TNE em pacientes submetidos a TCTH mieloablativo, estudo recente, com 28 pacientes em cada braço, mostrou que 92% dos pacientes em uso de TNE e 95% dos pacientes em uso de TNP apresentaram náuseas e vômitos. Apresentaram diarreia nos grupos com TNE e TNP, respectivamente, 44% e 54% dos pacientes. A TNE foi descontinuada em apenas 28% dos pacientes por alteração da imagem corporal, vômitos incoercíveis e deslocamento ou obstrução do cateter para alimentação enteral, quando já tinha desenvolvimento de mucosite, impossibilitando recolocar o cateter de alimentação.^242^ Tal observação demonstra a viabilidade do uso rotineiro de TNE em pacientes submetidos ao TCTH.

As sociedades americana, francesa e europeia concordam que a primeira opção de TN, quando o trato gastrintestinal está funcionante, deve ser a enteral, quando comparada à parenteral, seja pelo risco de complicações infecciosas ou por implicações metabólicas. O manejo das toxicidades gastrintestinais deve ser adotado como prática diária das equipes de saúde.^30,40,47^ As toxicidades gastrintestinais, como mucosite, não são limitativas para o uso da TNE, desde que a sonda seja previamente passada.^30^

### Infecção relacionada ao uso de cateter de alimentação enteral e terapia nutricional enteral

Comparativamente, TNE possui menor correlação com infecção que TNP.^242^ O risco de infecção relacionada ao uso de TNE advém da inserção do cateter para alimentação enteral, da manipulação inadequada da alimentação enteral, bem como do cateter de alimentação enteral, do armazenamento incorreto da alimentação a ser ofertada, e do reuso incorreto de equipos e frascos para alimentação.

### Inserção do cateter de alimentação enteral

Há três tipos de acesso mais comuns: nasogástrico, nasojejunal e gastrostomia endoscópica percutânea. O risco de infecção se correlaciona com a passagem do cateter (nasogástrica e nasojejunal), pelo contato com a mão do profissional de saúde que guia o cateter e, na gastrostomia, pelo procedimento de colocação do cateter e infecção do sito de inserção.

### Manipulação da alimentação enteral

A unidade de saúde deve seguir as recomendações das Boas Práticas de Produção de Nutrição Enteral, conforme Resolução da Diretoria Colegiada 63, de 2000, da ANVISA.^266^

O nutricionista é responsável pela supervisão da preparação da nutrição enteral. A preparação da TNE envolve a avaliação da prescrição dietética, a manipulação, o controle de qualidade, a conservação e o transporte da nutrição enteral, exigindo a responsabilidade e a supervisão direta do nutricionista. A manipulação da TNE deve ser realizada com técnica asséptica, seguindo procedimentos escritos e validados.

A TNE deve ser rotulada com identificação clara do nome do paciente, composição e demais informações legais e específicas, para a segurança de sua utilização e garantia de seu rastreamento. O nutricionista é responsável pela manutenção da qualidade da TNE até sua entrega ao profissional responsável pela administração e deve orientar e treinar os funcionários que realizam seu transporte.

Para a TNE em sistema fechado, devem ser seguidas as recomendações do fabricante quanto à conservação e ao transporte. O uso de um sistema fechado é geralmente preferido, uma vez que a alimentação é esterilizada, não há manipulação local e a quantidade acessos ao cateter é reduzida, diminuindo a chance de infecção.^287^

A alimentação enteral produz os mesmos organismos que causam intoxicação alimentar, com sintomas semelhantes, como desconforto abdominal e/ou distensão, náuseas, vômitos e diarreia, o que, no paciente imunocomprometido, pode levar à bacteriemia, septicemia e até morte.^277,288,289^

### Armazenamento e administração

O enfermeiro é o responsável pela conservação após o recebimento da TNE e por sua administração.^266^

A fim de reduzir riscos biológicos, a equipe de enfermagem deve:

- Proceder à inspeção visual da TNE antes de sua administração. Se verificada alguma anormalidade na nutrição enteral, a alimentação não deve ser administrada, o nutricionista responsável deve ser contatado, e a TNE deve ser devolvida.
- Confirmar a localização da sonda e sua permeabilidade, antes de iniciar a administração da nutrição enteral.
- Proceder à correta lavagem das mãos, antes de prosseguir na operacionalização da administração da nutrição enteral.
- Administrar a nutrição enteral, cumprindo rigorosamente o prazo estabelecido.
- Caso haja necessidade de pausa da alimentação, a TNE de sistema fechado deve obedecer às instruções dos fabricantes, com relação ao tempo de estabilidade após aberta. A TNE manipulada (sistema aberto) não deve permanecer por mais de 4 horas para ser infundida.^287^

A TNE é inviolável até o final de sua administração, não podendo ser transferida para outro tipo de recipiente. Sua administração deve ser executada de forma a garantir ao paciente terapia segura e que permita a máxima efetividade, em relação aos custos, utilizando materiais e técnicas padronizadas.

Existem várias maneiras pelas quais uma alimentação enteral pode ser contaminada no hospital, casa ou casa de saúde, como:^290^

- Má higiene das mãos: a falta de lavagem das mãos pode levar à contaminação da alimentação enteral, do cateter e do equipo e frasco de nutrição enteral.^290^
- Limpeza inadequada dos equipamentos reutilizáveis durante e após o uso: o equipo e a bomba de infusão devem ser mantidos limpos enquanto estiverem em uso e limpos completamente após a conclusão da alimentação.^287^
- Instalação inadequada da alimentação enteral: manuseio do cateter e do frasco sem técnica adequada.^287^
- Manutenção deficiente do cateter: é necessária lavagem regular do cateter com água filtrada para reduzir o acúmulo de resíduos de alimentação e medicação no cateter, o que pode causar bloqueio e se tornar fonte de contaminação. Os cateteres para alimentação enteral devem ser lavados com 20mL a 30 mL de água, a cada 4 a 6 horas, durante a alimentação contínua, nas pausas da alimentação e antes e após infusão de medicações.
- Utilização de equipo de forma inadequada: todo o equipo para alimentação enteral é concebido para este fim específico. O ideal é que o equipo para alimentação enteral não se conecte em qualquer outro acesso. É possível a administração de medicamentos pelo cateter de alimentação enteral; contudo, a equipe de farmácia do hospital deve orientar quanto à adequada manipulação e à adminstração de tais substâncias.

A educação e o treinamento em prevenção e controle de infecção são vitais.^288,291^ A prática dos enfermeiros é fundamental para prevenir a contaminação bacteriana na alimentação enteral.^290^ O treinamento do pessoal de enfermagem quanto aos procedimentos corretos de manuseio e protocolos de alimentação enteral pode reduzir o nível e a incidência de contaminação bacteriana na TNE, bem como melhorar a oferta da TNE e impactar o desfecho clínico do paciente de TCTH.^287,288,291,292^

### Reuso de materiais

Na maioria dos hospitais e casas de saúde, é recomendado uso único do equipo, descartando-o após uso. É da responsabilidade do enfermeiro garantir o descarte de qualquer equipamento utilizado. Deve ser considerado um mecanismo de segurança, que não deve ser negligenciado. Tais erros podem não parecer tão graves como erro na administração de drogas, por exemplo, mas o efeito de tais más práticas pode ser grave para os pacientes.^289,290^ A reutilização do equipo pode afetar sua integridade e aumentar a probabilidade de contaminação bacteriana.^290^

Outro produto que deve ser utilizado por uma única vez é a seringa, usada para lavagem das sondas. Caso haja a necessidade de reutilização das seringas, elas devem ser lavadas logo após o uso com água e sabão. O êmbolo deve ser removido para permitir a limpeza completa. Outros métodos de limpeza aprovados incluem máquina de lavar louça, imersão em água fervente (3 minutos), solução de esterilização a frio e esterilizador a vapor para microondas. Uma vez seca, armazene em um recipiente limpo e seco. Somente remonte a seringa antes de usar. Deve ser estritamente usada para um único paciente. As seringas são validadas para 30 utilizações. Para a maioria dos pacientes, a seringa deve ser trocada uma vez por semana (equivalente a quatro doses máximas por dia). No entanto, se usar a seringa mais de quatro vezes por dia, a seringa deve ser trocada mais regularmente.

### Desmame

A suspensão da TNE é dependente da aceitação alimentar por via oral, e o consumo oral deve atingir 60% ou mais das necessidades nutricionais por três dias consecutivos.^246^ Após a cessação das toxicidades gastrintestinais como mucosite, e o restabelecimento da alimentação por via oral, a ingestão oral deve ser estimulada e a terapia nutricional oral deve ser iniciada para completar a oferta e o consumo de nutrientes por via oral. Quando a soma do consumo por via oral, alimentação e suplementação oral for igual ou superior a 60% das necessidades nutricionais individuais, mantendo-se por três dias consecutivos, a TNE deve ser suspensa.^40,47,246^

### A importância do uso de indicadores de terapia nutricional

Os indicadores de TN proporcionam a gestão de qualidade na assistência nutricional prestada ao paciente. Esses indicadores permitem, por meio da análise constante dos seus resultados, avaliar as ações desempenhadas, possibilitando realizar a análise crítica, implantar medidas corretivas para melhoria dos resultados e da assistência prestada ao paciente.^293^

Ao serem implantados programas de garantia da qualidade em TN, alguns benefícios são reconhecidos, como grande eficiência nas rotinas diárias, redução de custos, prática de ações em saúde respaldada por literatura científica, maior capacidade de análise de processos, e melhores resultados clínicos e de qualidade de vida para o paciente.^293,294^

A seleção dos indicadores ocorre por conta da necessidade e das possibilidades de serem realizados no serviço.^278^ Relacionam-se aos apontamentos descritos no tópico de TNE os três indicadores apresentados nas tabelas 20 a 22 [Table t34002] [Table t35002], na forma de ficha técnica.

Nas figuras 1 e 2 são apresentados os fluxogramas para tomada de decisão.^39,47,119,242,246,295-297^

**Tabela 20:** Frequência de conformidade de indicação da terapia nutricional enteral (TNE)

Objetivo	Verificar se a TNE está indicada de acordo com as diretrizes preestabelecidas em protocolos para pacientes em TNE
**Descrição**	Mensurar a ocorrência de indicações de TNE, segundo diretrizes preestabelecidas em pacientes
**Justificativa**	Conhecer a frequência de indicação de TNE conforme diretrizes preestabelecidas e tomar medidas para que estas sejam seguidas a todos os pacientes que necessitam dessa terapia
**Fórmula**	Número de pacientes em TNE indicada conforme diretriz × 100 Número total de pacientes em TNE
**Frequência**	Mensal
**Meta**	<10%
**Responsável pela informação**	Nutricionista e Equipe Multidisciplinar de Terapia Nutricional

Fonte: Isosaki M, Gandolfo A, Jorge A, Evazian D, Castanheira F, Bittar O. Indicadores de Nutrição Hospitalar. São Paulo: Atheneu; 2015.^(294)^

**Tabela 21 t34002:** Frequência de pacientes em terapia nutricional enteral (TNE) que atingiram 100% das necessidades nutricionais em até 3 dias

	Monitorar o tempo que pacientes levam para atingir a adequação das necessidades calórico e/ou proteicas
Objetivo	
**Descrição**	Avaliar o número de pacientes atingiram a meta calórica e/ou proteica em até 3 dias do início da TNE
**Justificativa**	Monitorar o tempo até atingir a meta calórica e proteica do paciente
**Fórmula**	Número de pacientes em TNE que atingiram 100% da adequação das necessidades calóricas e/ou proteicas* × 100 Número total de pacientes em TNE
**Frequência**	Mensal
**Meta**	≥80% ou de acordo com as necessidades da unidade
**Responsável pela informação**	Nutricionista e Equipe Multidisciplinar de Terapia Nutricional

Fonte: Waitzberg DL, editor. Indicadores de qualidade nutricional: aplicação e resultados. São Paulo: Atheneu; 2010;^(293)^ Isosaki M, Gandolfo A, Jorge A, Evazian D, Castanheira F, Bittar O. Indicadores de Nutrição Hospitalar. São Paulo: Atheneu; 2015.^(294)^ * Em até 3 dias.

**Tabela 22 t35002:** Frequência de pacientes sob terapia nutricional enteral (TNE) que recuperaram ingestão oral

**Objetivo**	Medida da frequência de recuperação de ingestão oral em pacientes sob TNE
**Descrição**	Frequência de recuperação de ingestão oral exclusiva em pacientes em TNE, na quantidade de ingestão >60% das necessidades calóricas estimadas
**Justificativa**	Conhecer a frequência de pacientes em recuperação oral após terapia nutricional
**Fórmula**	Número de pacientes que recuperaram a alimentação oral* × 100 Número total de pacientes em TNE
**Frequência**	Mensal
**Meta**	>80%
**Responsável pela informação**	Nutricionista

Fonte: Isosaki M, Gandolfo A, Jorge A, Evazian D, Castanheira F, Bittar O. Indicadores de Nutrição Hospitalar. São Paulo: Atheneu; 2015.^(294)^ * ≥60% da necessidade calórica estimada.

**Figura 1:** Fluxograma para tomada de decisão em admissão para o transplante de células-tronco hematopoéticas ASG: Avaliação Subjetiva Global; ASG-PPP: Avaliação Subjetiva Global Produzida pelo Paciente; IDR: Ingestão Dietética Recomendada TNO: terapia nutricional oral; TNE: terapia nutricional enteral; DECH: doença do enxerto contra o hospedeiro; TNP: terapia nutricional parenteral.

**Figura 2:** Fluxograma para tomada de decisão em avaliação nutricional pré-transplante de células-tronco hematopoéticas (TCTH) ASG: Avaliação Subjetiva Global; ASG-PPP: Avaliação Subjetiva Global Produzida pelo Próprio Paciente; BIA: balão intra-aórtico; USG: ultrassonografia; TNO: terapia nutricional oral; TNE: terapia nutricional enteral.
